# AdipoR1/AdipoR2 dual agonist recovers nonalcoholic steatohepatitis and related fibrosis via endoplasmic reticulum-mitochondria axis

**DOI:** 10.1038/s41467-020-19668-y

**Published:** 2020-11-16

**Authors:** Hongjiao Xu, Qian Zhao, Nazi Song, Zhibin Yan, Runfeng Lin, Shuohan Wu, Lili Jiang, Sihua Hong, Junqiu Xie, Huihao Zhou, Rui Wang, Xianxing Jiang

**Affiliations:** 1 School of Pharmaceutical Sciences, Sun Yat-Sen University, 132 East Outer Ring Road, Guangzhou, 510006 China; 2 Key Laboratory of Preclinical Study for New Drugs of Gansu Province, School of Basic Medical Sciences, Lanzhou University, Lanzhou, 730000 China

**Keywords:** Structure-based drug design, Pharmacology, Non-alcoholic steatohepatitis, Drug development

## Abstract

Chronic nonalcoholic steatohepatitis (NASH) is a metabolic disorder that often leads to liver fibrosis, a condition with limited therapy options. Adiponectin is an adipocytokine that regulates glucose and lipid metabolism via binding to its receptors AdipoR1 and AdipoR2, and AdipoRs signaling is reported to enhance fatty acid oxidation and glucose uptake. Here, we synthesize and report an adiponectin-based agonist JT003, which potently improves insulin resistance in high fat diet induced NASH mice and suppresses hepatic stellate cells (HSCs) activation in CCl_4_ induced liver fibrosis. Mechanistic studies indicate that JT003 simultaneously stimulates AdipoR1- and AdipoR2- mediated signaling pathways as well as the *PI3K-Akt* pathway. Moreover, JT003 treatment significantly improves ER-mitochondrial axis function, which contributes to the reduced HSCs activation. Thus, the AdipoR1/AdipoR2 dual agonist improves both NASH and fibrosis in mice models, which provides the pharmacological and biological foundation for developing AdipoRs-based therapeutic agents on liver fibrosis.

## Abstract (web summary)

Nonalcoholic steatohepatitis (NASH) and associated liver fibrosis have limited therapy options. Here the authors report a novel adiponectin-based dual agonist for adiponectin receptors 1 and 2 with a longer half-life, and show that it ameliorates NASH and liver fibrosis in mouse models.

## Introduction

Nonalcoholic steatohepatitis (NASH), accompanied by different severity of inflammation and fibrosis, will process to end-stage liver disease like cirrhosis and hepatocellular carcinoma (HCC)^1^. It is also a serious risk factor for cardiovascular disease, portal hypertension, type 2 diabetes mellitus (T2DM) and severe kidney diseases, which confers a risk of poor outcome and significantly high mortality^1,2^.

NASH pathogenesis linked inflammasome directly or indirectly evokes the development of liver fibrosis^3^. Hepatocytes insulin resistance increases the uptake and synthesis of free fatty acids (FFAs) in liver, sensitizes the hepatocytes to oxidative damage, endoplasmic reticulum stress (ER stress) and mitochondrial dysfunction, which would activate hepatic stellate cells (HSCs) and result in liver fibrosis^4,5^. Various convergent pathways or factors are now considered to be implicated in NASH-associated fibrosis: oxidative stress, inflammasome activation, ER stress and mitochondrial dysfunction^3,5,6^.

In response to the etiology and pathogenesis of NASH, several medical treatments with different targets are being tested in clinical trials, including peroxisome proliferator-activated receptors (PPARs) agonists like saroglitazar and pioglitazone^7^, farnesoid X receptor(FXR) agonist obeticholic acid (OCA)^8^ and glucagon-like peptide-1 receptor (GLP1R) agonists and its analogs^9^. However, unacceptable side effects have been observed for those candidates, such as the cardiovascular, fracture and bladder cancer risk for PPAR agonists, the severe pruritus for FXR agonist obeticholic acid (OCA) as well as severe gastrointestinal disorders in NASH patients. Instead of fibrosis, GLP-1R agonists were being actively pursued for the treatment of NASH^9^. Other therapeutics like anti-LOXL2 antibodies^10^, caspase protease inhibitors^11^ and acetyl-CoA carboxylase inhibitors^12^ either exhibit serious side effects or have limited pharmaceutical effectiveness in NASH clinical trials. To date, there is no approved pharmacological therapy for liver fibrosis, highlighting the urgent requirements to develop effective therapeutic strategies for this disease. We believe this represents a considerable challenge.

In the past two decades, the discovery of protein adiponectin^13^ and its receptors has led to a great understanding of the development of metabolic disorders^14^. As the insulin sensitizer, the primary physiological action of adiponectin is to ameliorate insulin resistance. Adiponectin contains a highly conserved complement factor C1q-like globular domain (gAd), which is able to bind to and activate the membrane receptors AdipoR1 and AdipoR2^15^. AdipoR1 and AdipoR2 are both abundantly expressed in liver and skeletal muscle, and they share a 66.7% sequence similarity in full length protein and 86% similarity at ligand binding site (extracellular loop 1, ECL1)^16,17^. Upon binding to AdipoR1 and AdipiR2, adiponectin mediates fatty acid oxidation^14,18^ and glucose metabolism^19^, mainly via AMP-activated protein kinase (*AMPK*)^20,21^ and peroxisome proliferator-activated receptor α (*PPARα*)^22^ signal pathways. Besides of orchestrating glucose and lipid metabolism, *AMPK* directly mediates the mitochondrial biogenesis and homeostasis, which could remit the energy stress occurs in NASH^23^. Activation of hepatic *PPARα* by adiponectin/AdipoR2 signal was also reported to repress the pro-inflammatory genes expression^24^. Furthermore, AdipoR1 signal could activate hepatocytes *PI3K-Akt* pathway^25^, which was also reported to play the dominate role in regulating insulin resistance, cell proliferation and apotosis^26,27^. These findings imply that the AdipoRs system is a potential drug target for treating NASH and related fibrosis.

In the current study, we design and synthetize a series of peptides based on gAd and gAd-derived peptides described previously^28,29^. The simultaneously binding of our peptides with AdipoR1 and AdipoR2 are confirmed by cell-surface binding assays using fluorescence labeled peptides. In general, the result of cell-based assays has been proved that these dual agonist peptides exhibit more promising anti-lipogenesis and anti-fibrogenesis effects than the single AdipoR1 peptide previously described, and the peptide JT003 shows the best activity. The potential therapeutic effects of JT003 have been further demonstrated in high fat diet induced NASH and CCl_4_ induced liver fibrosis mouse models. We confirm that JT003 could ameliorate the progress of NASH and related fibrosis via *AMPK* and *PPARα* signal pathways. Besides, activation of *PI3K-Akt* pathway by JT003 treatment also contributes to the amelioration of insulin resistance. The inhibition of *PERK*, *eIF2α* activities and the up regulation of *PGC1α* expression demonstrate that JT003 regulates endoplasmic reticulum (ER)-mitochondrial function in both NASH and fibrotic mice. Taken together, our studies show that AdipoR dual agonist JT003 could provide compelling pharmaceutical effect and rational pharmacological mechanism in recovering NASH and liver fibrosis.

## Results

### Design of the gAd-based AdipoR agonists and the study

**Fig. 1:**
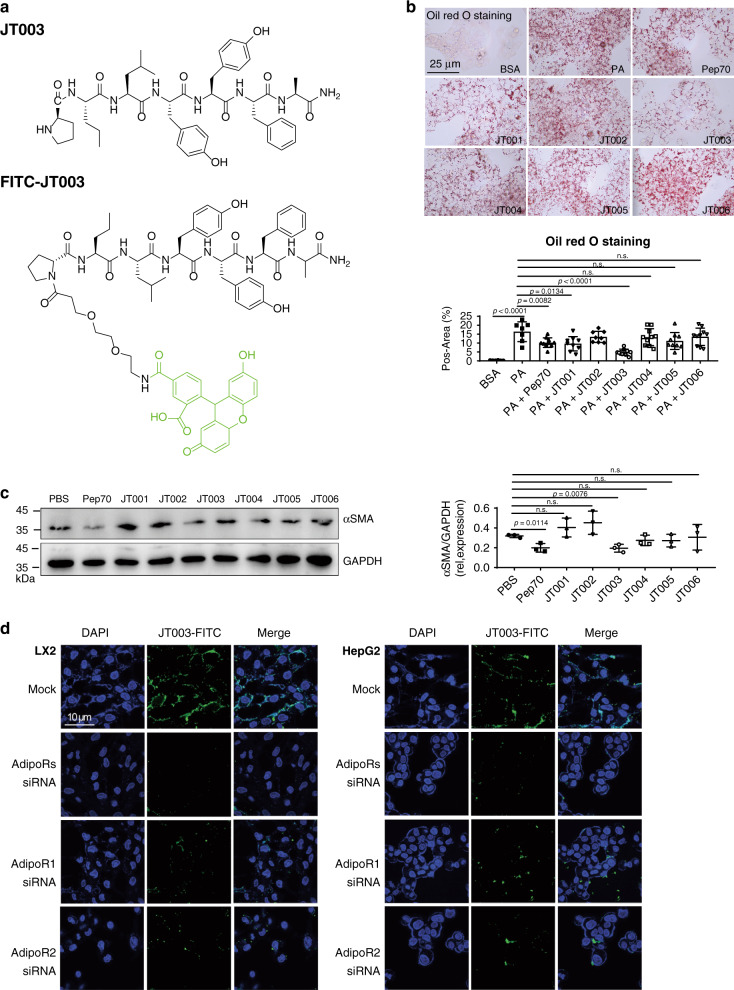
Peptides design and activity study. **a** Chemical structures of JT003 and FITC-JT003. **b** Oil Red o staining of PA induced lipid accumulation in HepG2 cell line after treated with 200 μM peptides. Triplicates were performed. **c** Western blotting for *αSMA* expression in LX2 after peptides treated. Three separated experiments were performed. *αSMA* expression was normalized to that of GAPDH. Positive areas were analyzed with ImageJ. **d** HepG2 and LX2 cells were incubated with FITC-JT003 at 37 °C for 2 or 4 h. The nucleus was stained with Hochest33342. The green channel represents as FITC. For each experiment, triplicates were performed. For in vitro experiment, the concentration of JT003 is 200 μM. Here and later, for each assay, three separated experiments were performed. And for each experiment (*n* = 4 cell samples/group or *n* = 6 mice/group). For the in vivo experiment, the dose of JT003 is 500 μg kg^−1^. All the above data are presented as mean values ± SEM using unpaired Student’s *t* test. Source data are provided as a Source Data file.

Previous peptide mapping has identified the gAd sequence between Lys149 and Val166 as the adiponectin active site^30,31^, and led to the development of ADP355, the first adiponectin receptor peptide-based agonist acting on both AdipoR1 and AdipoR2^31^. In addition, a following study showed that a shorter heptapeptide derived from the active site of adiponectin, P70, is sufficient to activate AdipoR1 efficiently and demonstrated anti-fibrotic activity^29^. On the top of this native sequence, appropriate chemical modifications were introduced for preferable pharmacological properties of the analogs (Supplementary Fig. 1 and Fig. 1a). Tyr159 was replaced with Ser to improve the solubility of the peptides (i.e., JT001, JT002, JT004 and JT006). The acidic amino acid Asp was applied to substitute Ala161 to obtain negative charged peptides (i.e., JT001, JT005 and JT006). Additionally, unnatural amino acid Nva was incorporated to improve the stability of the peptides and prolong their half-life in serum (i.e., JT003, JT004, JT005 and JT006). The results indicated that none of them exhibited significant cytotoxicity to LX2 cells even at the highest concentration tested (1024 µM) (Supplementary Fig. 2a). 2–4096 µM of peptides were incubated with mice hemocyte for 1 h to evaluate the hemolytic activity, and no hemolysis was observed (Supplementary Fig. 2b). No toxicity was exhibited to both LX2 cells and hemocyte, ensured the feasibility of the following activity studies in vitro and in vivo.

To evaluate the anti-lipid accumulation and anti-fibrogenic effects of these analogs, a PA induced HepG2 cell model and a human HSCs derived LX2 cell model were employed. ADP355, which shares sequence similarity with JT003 and has exhibited anti-fibrotic activity^28,31–33^ was used as a control in these and subsequent experiments. Lipid droplets accumulation caused by PA could be reduced by the native gAd_155-161_ peptide and the analogs we designed as well as previously reported peptide ADP355^28,29^, of which JT003 exhibited the best potential (Fig. 1b, Supplementary Fig. 3). Western blotting assay confirmed that JT002, JT003 and JT004 could significantly decrease the expression level of *αSMA*, a marker of fibroblast, in LX2 cells, and, again, JT003 showed the best performance in inhibiting fibroblast formation (Fig. 1c, Supplementary Fig. 4). The stability of JT003 in mouse serum was tested with RP-HPLC. As shown in Supplementary Fig. 5, after incubation in serum for 12 h, JT003 still had a retention rate of about 22%, indicating that JT003 is a long stabling peptide suitable for further in vivo test. Thus, JT003 was selected for further study.

To investigate the binding specificity of JT003 to AdipoR1 and AdipoR2, a fluorescent probe was synthesized by conjugating FITC to JT003 (Fig. 1a). After incubation with FITC-JT003, AdipoR1 and AdipoR2 expressed HepG2 and LX2 cells were tightly labeled with fluorescent probe on the cytomembrane (Fig. 1d). The suppression of AdipoR1 and/or AdipoR2 expression by specific siRNA blocked the binding of fluorescein to both cells, providing us the direct evidence that JT003 could specifically bind to AdipoR1 and/or AdipoR2 on the cell membrane. On the contrary, JT006, which showed the least *αSMA* signal (Fig. 1c) and little prevention of palmitic acid induced lipid droplets (Fig. 1b), could not bind to the AdipoRs specifically (Supplementary Fig. 6). To better understand how JT003 can bind to AdipoR1 and AdipoR2, the peptide was docked into the crystal structures of AdipoR1 (PDB code: 5LXG) and AdipoR2 (PDB code: 5LXG)^17^ using MOE. Supplementary Fig. 7 showed the key residues involved in the binding sites of each receptors. The computational and the above cell-based results strongly suggested that JT003 is an AdipoR1 and AdipoR2 agonist with great potential in ameliorating NASH and related fibrosis.

### JT003 ameliorates obesity and insulin resistance in NASH

**Fig. 2:**
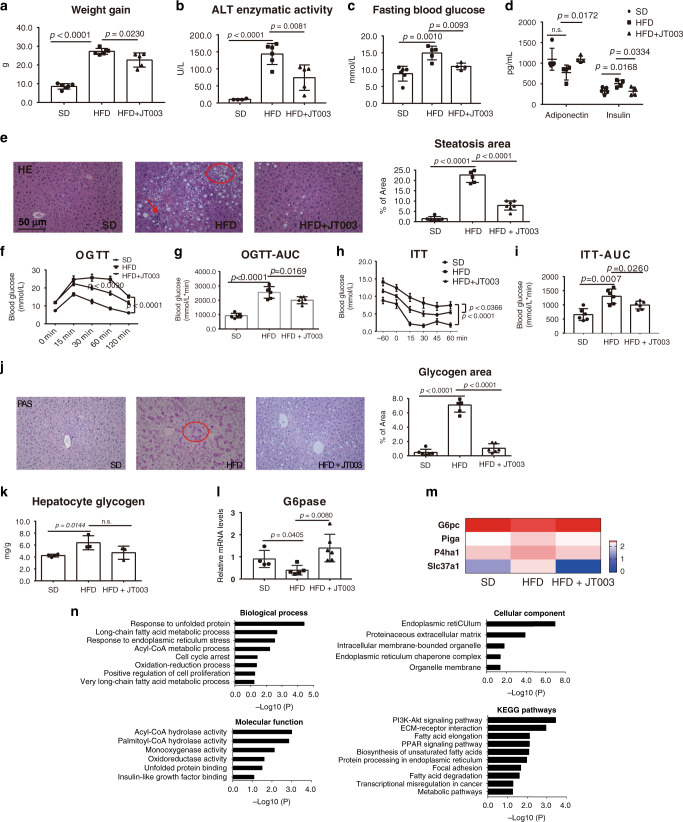
JT003 ameliorates obesity and insulin resistance of HFD-induced NASH. **a, b** Weight gain and serum ALT activity of mice treated with SD, HFD or HFD plus JT003 therapy. **c**, **d** Fasting blood glucose levels were recorded, fasting insulin and fasting adiponectin were measured by enzyme-linked immunosorbent assay (ELISA) in serum of mice treated with SD, HFD or HFD plus JT003 therapy. **e** Representative images of HE staining of liver sections on mice of indicated groups (red arrow represents the inflammation area; red circle represents the ballooning area) and steatosis area. Positive areas were analyzed with ImageJ. **f–i** Oral glucose tolerance test (OGTT) and insulin tolerance test (ITT) were performed at the 14th or 15th week of food administered, respectively. The corresponding area under curves (AUC) of blood glucose levels were calculated. **j** Representative images of periodic acid-schiff (PAS) staining on mice of indicated groups (red circle represents the glycogen area) and statistical glycogen area. The glycogen content (**k**) and the mRNA level of *G6Pase* (**l**) were measured in the liver samples of mice treated with SD, HFD or HFD plus JT003 therapy. (**m**) Genes related to glucose metabolism significantly up-regulated and down-regulated in RNAseq analysis (*q* < 0.001). **n** GO and KEGG pathway analysis. Scale bar in pane c applies to all images. Here and later, for each assay, three separated experiments were performed. And for each experiment (*n* = 4 cell samples/group or *n* = 6 mice/group). For the in vivo experiment, the dose of JT003 is 500 μg kg^−1^. All the above data are presented as mean values +/− SEM using unpaired Student’s *t* test. Source data are provided as a Source Data file.

The impaired glucose and lipid metabolism was reported to contribute to insulin resistance in NASH individuals^34^. We next examined the anti-NASH progress effect of JT003 in vivo (Supplementary Fig. 8a). The *C57BL/6J* mice (*n* = 6 per group) fed with high fat diet (HFD) for 16 weeks presented anticipated features of NASH with obesity, elevated ALT and insulin resistance compared to mice fed standard diet (SD). Treatment with JT003 significantly reduced the weight gain, smoothed the liver and decreased the liver sizes (Fig. 2a, Supplementary Fig. 8b). The ALT level were also reduced in HFD fed mice after JT003 treatment (Fig. 2b). Besides, in HFD combine with methionine and choline deficient (MCD) diet NASH mouse model, JT003 inhibited lipid accumulation significantly with a 20 times lower dosage than the previous reported peptide ADP355 (Supplementary Figs. 9 and 10). JT003 also decreased the HFD-induced increasing of fasting blood glucose and fasting serum insulin levels. And the decrease in circulating adiponectin in the HFD group was reverted remarkably after JT003 treating (Fig. 2c, d). HE staining showed that both steatosis and inflammation area were decreased by the treatment of JT003 (Fig. 2e). Moreover, oral glucose tolerance test and subcutaneous insulin tolerance test demonstrated that HFD-induced insulin resistance was obviously ameliorated by administration of JT003 (Fig. 2f–i).

The impaired hepatic glycogen metabolism was reported to contribute to the insulin resistance^35^. In our research, periodic acid-schiff (PAS) staining and hepatocyte glycogen analysis both displayed that glycogen over accumulated in HFD fed mice, which was largely eliminated by JT003 treating (Fig. 2j, k). *G6Pase* expression level in JT003 exposed mice liver was significantly elevated compared to HFD mice, suggesting that JT003 increases glycogenolysis (Fig. 2l). RNAseq gene expression analysis was performed to look for the pathways in livers of HFD fed mice that were affected by JT003 treatment, and the gene enrichment analysis identified that gene sets related to glycose metabolism (Fig. 2m).

Taken together, our observation indicated that HFD-induced insulin resistance was inhibited by the AdipoRs dual agonist JT003, which improved the energy availability in NASH mice. Furthermore, the GO analysis revealed that NASH mice treated with JT003 affects a list of genes associated with metabolic process, endoplasmic reticulum stress and mitochondrial functions, and the KEGG pathways analysis stated that *PI3K-Akt* signal might be involved during JT003 therapy (Fig. 2n).

### JT003 recovers NASH via *AMPK*, *PPARα*, *PI3K-Akt* pathways

**Fig. 3:**
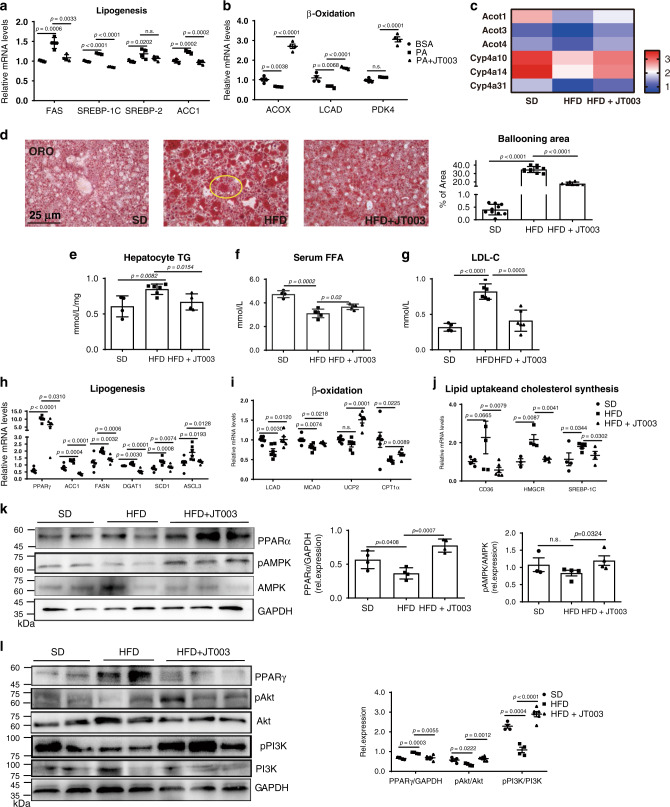
JT003 improves lipid metabolism via *AMPK*, *PPARα* and *PI3K-Akt-PPARγ* pathways. **a**, **b** Quantitative RT-PCR assay was performed to determine mRNA expression of genes related to lipogenesis, lipid β-oxidation in HepG2 cells of the indicated groups. PA treated cells were considered as model group, BSA treated cells were negative control group. **c** Genes related to *AMPK* and *PI3K-Akt* signal pathways significantly up-regulated and down-regulated in RNAseq analysis on mice treated with SD, HFD or HFD plus JT003 therapy (*q* < 0.001). **d** Representative images of oil red o staining of liver sections on mice treated with SD, HFD or HFD plus JT003 therapy (yellow circle represents the lipid droplets). Positive areas were analyzed with ImageJ. **e**–**g** Hepatic TG, serun FFA and LDL-C content of mice in the indicated groups were measured. **h–j** mRNA level of genes related to lipogenesis, lipid β-oxidation, lipid uptake and cholesterol synthesis in liver samples of the indicated groups. **k** The expression level of key protein *PPARα*, *AMPK/pAMPK* in the liver of the indicated groups. Here and later, unless otherwise indicated, the quantifications of protein expression level were performed using three independent western blotting experiments. Proteins expression were normalized to that of *GAPDH*. **l** The expression level of key proteins *PI3K/pPI3K*, *Akt/pAkt* and *PPARγ* in *PI3K-Akt* signaling. For in vitro experiment, the concentration of JT003 is 200 μM. For the in vivo experiment, the dose of JT003 is 500 μg kg^−1^. Here and later, for each assay, three separated experiments were performed. And for each experiment (*n* = 4 cell samples/group or *n* = 6 mice/group). For the in vivo experiment, the dose of JT003 is 500 μg kg^−1^. All the above data are presented as mean values ± SEM using unpaired Student’s t test. Source data are provided as a Source Data file.

It was reported that AdipoRs could mediate the lipid metabolism, possibly through activating *AMPK* and *PPARα* signal pathways^20^. We then observed that if JT003 could activate the intended targets. In PA induced HepG2 NASH cell models, lipogenesis (*FAS*, *SREBP-1C*, *SREBP-2* and *ACC1*) and fatty acid β-oxidation (*ACOX*, *LCAD* and *PDK4*) were meliorated notably with JT003 exposed (Fig. 3a, b). In HFD induced NSAH mice, RNAseq gene expression analysis showed gene sets related to lipid metabolism were altered in response to JT003 treatment (Fig. 3c). Oil red o (ORO) staining showed remarkably decreased lipid droplets in the liver of JT003 treated mice (Fig. 3d). Hepatocytes TG and serum LDL-C contents were obviously reduced by JT003. Moreover, the decrease of the circulating lipid in the HFD group was reverted in JT003 treated mice (Fig. 3e–g). Meanwhile, the expression of genes related to fatty acid synthesis (*PPARγ*, *ACC1*, *FASN*, *DGAT1*, *FAS*, *SCD1*, *ASCL3*), fatty acid uptake (*CD36*) and cholesterol synthesis (*HMGCR*, *SREBP-1C*) were significantly inhibited by JT003 treatment whereas the expression of genes responsible for fatty acid β-oxidation (*LCAD*, *MCAD*, *UCP2*, *CPT1α*) were much higher in JT003 treated mice (Fig. 3h–j). Western blotting results revealed that with JT003 treating, *AMPK* and *PPARα* signaling were activated apparently in NASH mice (Fig. 3k). Previous studies reported that *PI3K-Akt* cascade also mediate lipid metabolism^36^. Thus, we also monitored the status of *PI3K* and *Akt* phosphorylation in liver tissue. Our results indicated that *PI3K* and *Akt* phosphorylation were increased with JT003 treatment, and the expression of downstream factor *PPARγ* which stimulates lipid uptake and adipogenesis was in turn inhibited (Fig. 3l).

All of these results provide the strong supporting evidence that JT003 improves lipid metabolism in NASH mice through increasing of *AMPK* and *PPARα* as well as the *PI3K-Akt-PPARγ* signal activity.

### JT003 recovers NASH by ameliorating ER-mitochondrial axis

**Fig. 4:**
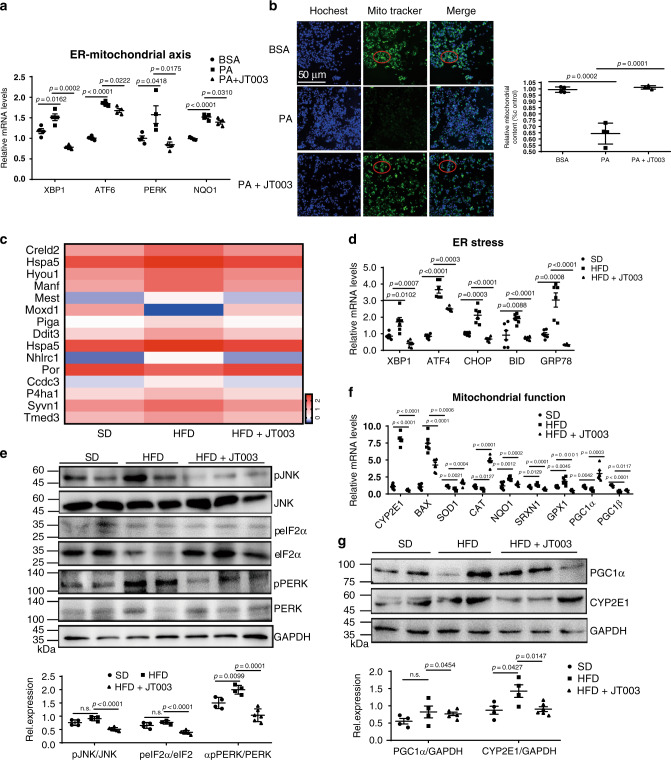
JT003 recovers NASH by ameliorating ER-Mitochondrial axis function. **a** Quantitative RT-PCR assay was performed to determine mRNA expression of genes related to ER-mitochondrial axis in HepG2 cells of the indicated groups. **b** HepG2 cells of the indicated groups were stained with Mito Tracker followed by assessment with mitochondrial content assay. **c** Genes related to ER-mitochondrial axis which were significantly up-regulated or down-regulated in RNAseq analysis (*q* < 0.001). **d**, **f** mRNA transcription of the genes related to ER stress and mitochondrial function in the liver samples of mice treated with SD, HFD or HFD plus JT003 therapy. **e** The phosphorylation of *PERK*, *eIF2α* and *JNK* in response to ER-stress. **g** The expression level of *PGC1α* and *CYP2E1* in the indicated groups. Here and later, for each assay, three separated experiments were performed. And for each experiment (*n* = 4 cell samples/group or *n* = 6 mice/group). For the in vivo experiment, the dose of JT003 is 500 μg kg^−1^. All the above data are presented as mean values ± SEM using unpaired Student’s t test. Source data are provided as a Source Data file.

ER and mitochondrial activity are essential in the mediation of NASH process^37^, besides, AdipoRs influence mitochondrial function via *AMPK* signaling^38,39^. In PA induced HepG2 cells, inflammatory factors and ER-mitochondrial axis dysfunction were meliorated notably with JT003 exposure (Supplementary Fig. 11a and Fig. 4a). Besides, mitochondrial content assay indicated that mitochondrial biogenesis was improved by JT003 (Fig. 4b). In present study, RNAseq gene expression analysis shown gene sets involved in ER Stress and mitochondrial function in response to JT003 treatment (Fig. 4c). Additionally, we investigated the ER-mitochondrial axis in JT003 treated NASH mice. As shown in Supplementary Fig. 11b and Fig. 4d, the overexpression of genes related to inflammation (*LY6G*, *F4/80*, *IL-6*, *IL-1β*, *iNOS*, *CD14*) and ER stress (*XBP1*, *ATF4*, *CHOP*, *BID* and *GRP78*), which was induced by HFD treatment, was suppressed significantly by JT003 administration. The phosphorylation of *PERK* and its downstream *eIF2α* and *JNK* which are the key roles of ER-stress pathways, were decreased notably after JT003 treatment (Fig. 4e). *PERK* was reported to be enriched at the mitochondrial-associated ER membranes, and it physically and functionally links ER and mitochondrial^40^. Since ER stress was remarkably released, we further investigated JT003 effect on mitochondrial function in NASH. The upregulation of genes related to mitochondrial dysfunction (*CYP2E1*, *BAX*, *NQO1*, *SRXN1* and *GPX1*) in NASH were suppressed remarkably by JT003. Consistently, the expression of antioxidant enzymes (SOD and CAT) and mitochondrial biogenesis regulators (*PGC1α* and *PGC1β*) were accelerated prominently with JT003 treatment (Fig. 4f, g). These results showed that ER-mitochondrial axis in NASH mice indeed contributed to the recovery of NASH process.

### JT003 inhibits the progress of CCl_4_-induced liver fibrosis

**Fig. 5:**
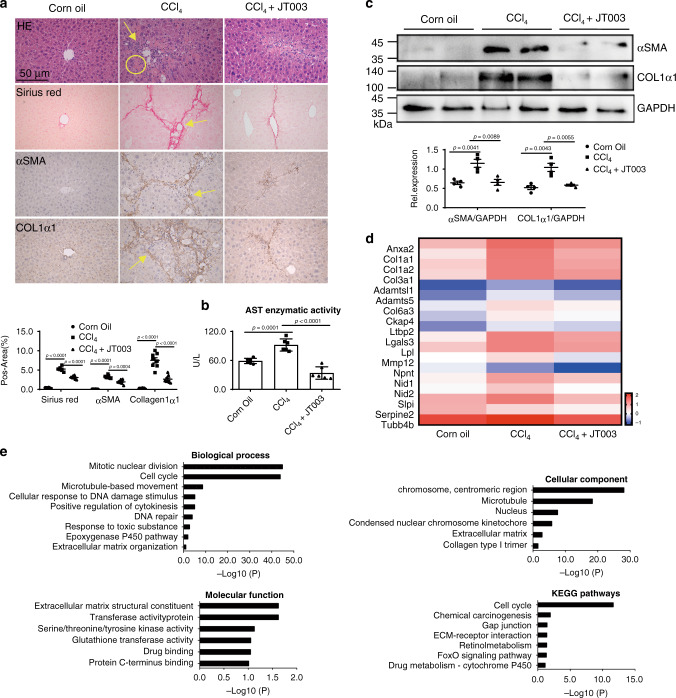
JT003 inhibits the progress of CCl_4_-induced liver fibrosis. **a**, **b** Representative images of HE staining, sirius red staining and IHC for *αSMA* and *COL1α1* of liver sections and ALT activity on mice treated with Corn Oil, CCl_4_ or CCl_4_ plus JT003 therapy. **c** Western blotting assay of *αSMA* and *COL1α1* measured in liver samples of mice treated with Corn Oil, CCl_4_ or CCl_4_ plus JT003 therapy. GAPDH serves as the loading control. **d** The fibrogenesis-related genes that significantly up-regulated or down-regulated in RNAseq analysis (q < 0.001). **e** GO and KEGG pathway analysis. For in vitro experiment, the concentration of JT003 is 200 μM. For the in vivo experiment, the dose of JT003 is 500 μg kg^−1^. Here and later, for each assay, three separated experiments were performed. And for each experiment (*n* = 4 cell samples/group or *n* = 6 mice/group). For the in vivo experiment, the dose of JT003 is 500 μg kg^−1^. All the above data are presented as mean values ± SEM using unpaired Student’s t test. Source data are provided as a Source Data file.

Lipid metabolism disorders change the gene expression in liver cells, the injured steatotic hepatocytes induce fibrosis genesis^41^. Then, we examined whether JT003 could ameliorate hepatic fibrosis in mouse models. Experiment was designed as shown in Supplementary Fig. 12a. Dissected assays indicted the liver adhesion were reduced by JT003 treatment, but not ADP355 (Supplementary Fig. 12b). There is no significant change in bodyweight (Supplementary Fig. 12c). HE and sirius red staining assays revealed that the inflammation, necrosis and fibrosis area in CCl_4_ induced liver fibrosis were significantly decreased by JT003 with the administration dosages of 0.5 mg kg^−1^ and 2 mg kg^−1^. In contrast, these effects were not notably raised employing the same dosages of ADP355 (Supplementary Fig. 13 and Fig. 5a). Immumohistochemical staining for *αSMA* and *COL1α1* confirmed the improvement of liver fibrosis by JT003 (Fig. 5a). Consistently, western blot assay confirmed the significant downregulation of *αSMA* and *COL1α1* (Fig. 5c). In serum biochemical analysis, ALT (Fig. 5b), AST and HYP (Supplementary Fig. 14) activities were all notably diminished after JT003 treatment, which further manifested the improvement of liver function. RNAseq gene expression analysis shown gene sets involved in fibrogenesis were screened in response to JT003 management (Fig. 5d). The GO analysis revealed that liver fibrosis melioration by JT003 affects a list of genes associated with hepatic fibrosis progress and the KEGG pathways analysis illustrated fibrosis progress pathways (Fig. 5e). From all the aspects above, JT003 treatments were clearly demonstrated to attenuate the development and progression of CCl_4_ induced liver fibrosis.

### JT003 suppressed the activation of HSC in fibrosis model

**Fig. 6:**
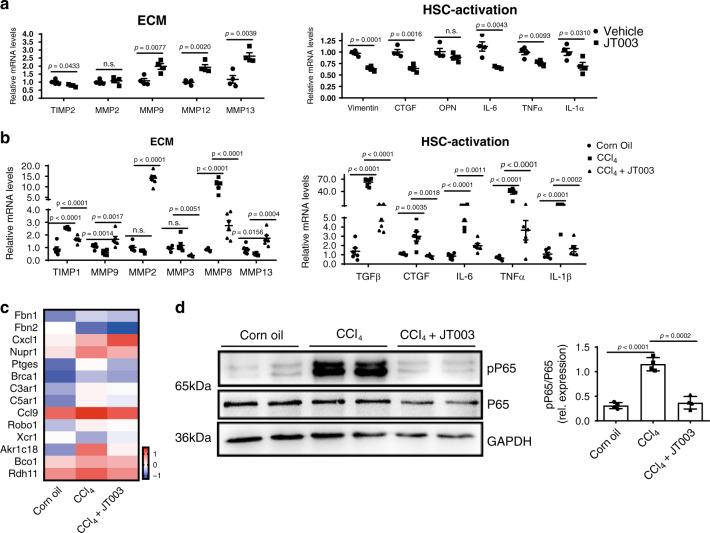
JT003 suppressed the activation of HSCs in CCl_4_-induced liver fibrosis. **a** Quantitative RT-PCR assay was performed to determine mRNA transcription of the genes related to ECM accumulation and HSC activation in the indicated groups. **b** mRNA transcription of the genes related to ECM accumulation and HSC activation in the liver of mice treated with Corn Oil, CCl_4_ or CCl_4_ plus JT003 therapy. **c** The HSC activation related genes which were significantly up-regulated and down-regulated in RNAseq analysis (*q* < 0.001). **d** Western blotting assay of total *NFκB (P65)* and *phosphorylated NFκB (pP65)* measured in liver samples of mice in the indicated groups. For in vitro experiment, the concentration of JT003 is 200 μM. For the in vivo experiment, the dose of JT003 is 500 μg kg^−1^. Here and later, for each assay, three separated experiments were performed. And for each experiment (*n* = 4 cell samples/group or *n* = 6 mice/group). For the in vivo experiment, the dose of JT003 is 500 μg kg^−1^. All the above data are presented as mean values ± SEM using unpaired Student’s *t* test. Source data are provided as a Source Data file.

Transdifferentiation of HSCs from quiescent into proliferative, fibrogenic myofibroblasts is widely accepted as the central driver of liver fibrosis. HSC-ECM interactions regulate cell differentiation and proliferation, generating increased fibrosis^42^. We then checked whether the JT003 amelioration of liver fibrosis depended on the inhibition of HSCs activation. As shown in Fig. 6a, in LX2 fibrosis cells, expression of genes involved in ECM accumulation (*TIMP1*) and degradation (*MMP9*, *MMP12*, *MMP13*) were notably altered via JT003 exposure. And HSC activation factors (*Vimentin*, *CTGF*, *OPN*, *IL-6*, *TNFα* and *IL-1α*) were significantly suppressed by JT003. In fibrosis mice, consistently, the expression of genes related to ECM accumulation (*TIMP1*, *MMP3* and *MMP8*) were decreased and genes involved in ECM elimination (*MMP2*, *MMP9* and *MMP13*) were notably upregulated after JT003 treatment. *TGFβ*, *CTGF*, *IL-6*, *TNFα* and *IL-1β* which contributed to HSCs activation were all suppressed by the administration of JT003 (Fig. 6b). RNAseq gene expression analysis exhibited that several other genes related to HSCs activation positively responded to JT003 treatment (Fig. 6c). Western blotting assays for *NFκB*, another major HSCs activation factor, was significantly downregulated by JT003 (Fig. 6d). Thus, we proposed that the inhibition of NASH related fibrosis by AdipoR1/2 agonist JT003 may result from suppression of HSCs activation.

### JT003 attenuates ER stress and mitochondrial dysfunction

**Fig. 7:**
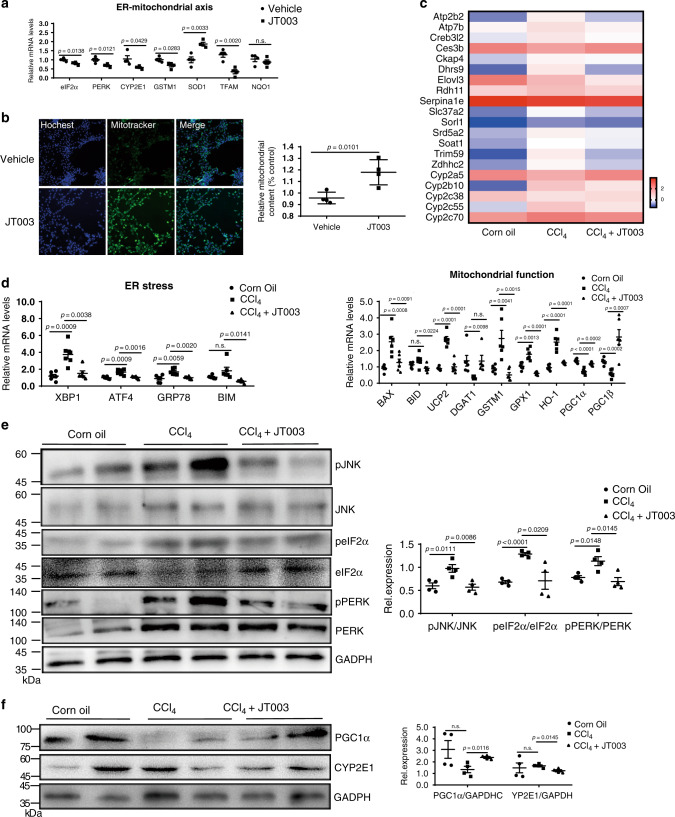
JT003 recovers CCl_4_-induced liver fibrosis via ER-mitochondrial axis. **a** mRNA transcription of the genes related to ER-mitochondrial axis in LX2 cells. **b** LX2 cells were stained with Mito Tracker followed by assessment with mitochondrial content assay. **c** ER-mitochondrial axis related genes that significantly up-regulated and down-regulated in RNAseq analysis (*q* < 0.001). **d** mRNA transcription of the genes related to ER-stress and mitochondrial function in the liver of mice in the indicated groups. **e**, **f** The expression level of key proteins *pJNK/JNK*, *peIF2α/eIF2α*, *pPERK/PERK*, *PGC1α* and *CYP2E1* in response to ER stress and mitochondrial dysfunction. For in vitro experiment, the concentration of JT003 is 200 μM. For the in vivo experiment, the dose of JT003 is 500 μg kg^−1^. Here and later, for each assay, three separated experiments were performed. And for each experiment (*n* = 4 cell samples/group or *n* = 6 mice/group). For the in vivo experiment, the dose of JT003 is 500 μg kg^−1^. All the above data are presented as mean values ± SEM using unpaired Student’s t test. Source data are provided as a Source Data file.

As aforementioned, JT003 could regulate ER-mitochondrial axis to recover HFD induced steatohepatitis. ER stress and mitochondrial dysfunction were clarified to involve in HSC activation^42^. Thus, we investigated whether the ER-mitochondrial axis was also involved in JT003 recovering of liver fibrosis. In activated human HSCs cell line LX2, inflammation related genes (*TNFα*, *IL-1α*, *IL-6* and *iNOS*) were suppressed with JT003 treatment (Supplementary Fig. 15a), and the expression of ER-mitochondrial function-related markers were also improved by JT003 (Fig. 7a). Moreover, with exposed to JT003, HSCs mitochondrial content was remarkably elevated by about 20%, indicating the function recover of mitochondrial (Fig. 7b).

For the in vivo assay, IHC staining of *CD68*, the marker of macrophage infiltration, demonstrated that JT003 administration markedly reduced the inflammation (Supplementary Fig. 15b), suggesting that CCl_4_ induced intense inflammation in liver was decreased. RNAseq gene expression analysis exhibited that several genes related to ER-mitochondrial axis were regulated in response to JT003 treatment in fibrosis mice (Fig. 7c). Additionally, JT003 remarkably suppressed the ER-Stress related (*XBP1*, *ATF4*, *GRP78* and *BIM*) and regulated mitochondrial function related genes (*BAX*, *BID*, *UCP2*, *DGAT1*, *GSTM1*, *GPX1*, *HO-1*, *PGC1α* and *PGC1β*) at both mRNA and protein expression levels in CCl_4_ induced fibrosis mice (Fig. 7d). Besides, the decreased phosphorylation levels of *JNK*, *eIF2α* and *PERK* in ER stress signal pathways and DNA fragmentation further confirmed the recovering effects of JT003 on ER stress and the following cellular apoptosis (Fig. 7e and Supplementary Fig. 16). Consistently, as revealed by RT-PCR assay, apoptosis-related *FAS* which was reduced and the anti-apoptotic *BCL2* was significantly improved by JT003 (Supplementary Fig. 16). The regulation of *CYP2E1* and *PGC1α* verified the improvement effects of JT003 on mitochondrial function (Fig. 7f). Combining all the results, we proposed that recovering the ER-mitochondrial functions by JT003 facilitated the recovering of NASH related fibrosis.

### Targeting AdipoR1/AdipoR2 and PK test

**Fig. 8:**
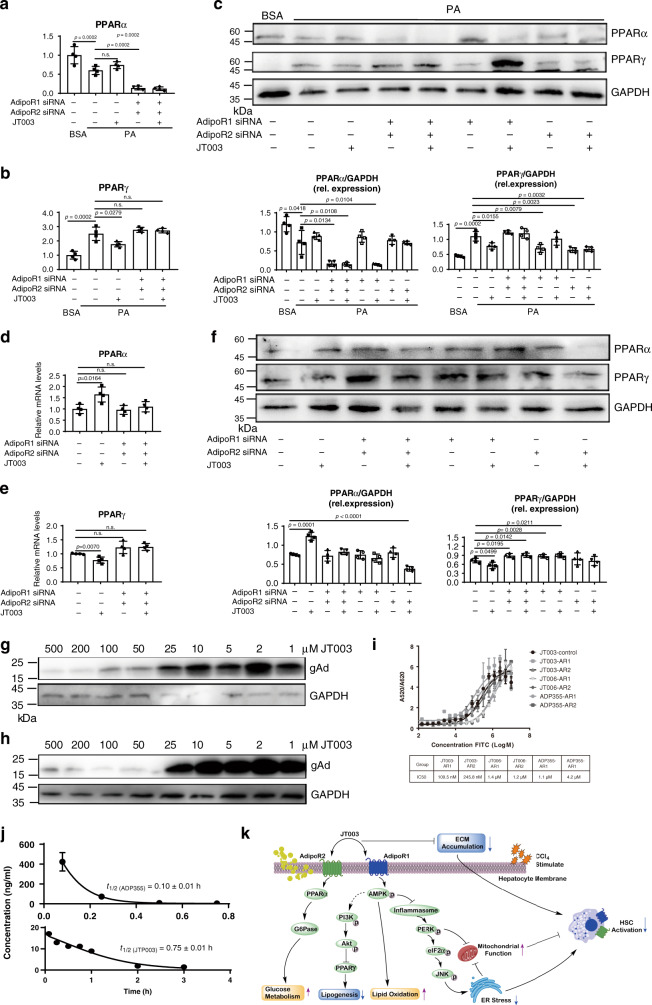
Suppression of AdipoR1/AdipoR2 blocks the downstream pathways activated by JT003. **a–f** The mRNA and protein expression level of *PPARα* and *PPARγ* in HepG2 (**a**, **b**, **c**) and LX2 cells (**d**, **e**, **f**) of the indicated groups with AdipoR1 and/or AdipoR2 siRNA treatment. **g**, **h** Competitive binding of JT003 to globular adiponectin (gAd) AdipoR1 overexpression cells and AdipoR2 overexpression cells. **i** The K_d_ value of JT003, JT006 and ADP355 bind to AdipoR1 and AdipoR2 (*n* = 2/group). **j** Plasma concentration versus time profiles of ADP355 and JT003 in SD rats following a single intravenous dose of 0.5 mg kg^−1^ and 1.0 mg kg^−1^, respectively (*n* = 3/group). **k** Scheme of AdipoR1/AdipoR2 dual agonist JT003 in recovering NASH and related fibrosis. JT003 binds to AdipoR1 and AdipoR2, improves glucose and lipid metabolism, suppresses HSCs activation and modulates the ER-mitochondria axis function in the development of NASH and related fibrosis. Here and later, for each assay, three separated experiments were performed. And for each experiment (*n* = 4 cell samples/group). For the in vivo experiment, the dose of JT003 is 500 μg kg^−1^. All the above data are presented as mean values ± SEM using unpaired Student’s *t* test. Source data are provided as a Source Data file.

To clarify whether the therapeutic effects of JT003 relied on activating AdipoR1 and AdipoR2, RNA interference experiments were performed in NASH and fibrosis cell models. As showed in Fig. 8a–c, in PA induced HepG2 cells, specifically suppressed AdipoR1 and/or AdipoR2 by specific siRNAs blocked the JT003 effects on mediating *PPARα* and *PPARγ* expression. In LX2 cells, suppression of AdipoR1 and AdipoR2 by specific siRNAs reduced the increase of the *PPARα* level induced by JT003, and these low expression state could not be recovered by JT003, indicating that JT003 upregulated *PPARα* expression via both AdipoR1 and AdipoR2. Besides, *PPARγ* expression level was increased following AdipoR1 and/or AdipoR2 knockdown, and these alterations could not be recovered by JT003, suggesting JT003 acted as a dual agonist of both AdipoR1 and AdipoR2 (Fig. 8d–f). With the competition binding assay, we found that more JT003 cause less globular adiponectin binding, indicated that JT003 could bind to AdipoRs specifically (Fig. 8g, h). Additionally, K_d_ value was determined via homogeneous time resolved fluorescence (HTRF) experiment (Fig. 8i). Compare to JT006 and ADP355, JT003 can bind to AdipoR1 and AdipoR2 in a significantly lower K_d_ value.

Besides, the pharmacokinetic study demonstrated that JT003 has more favorable pharmacokinetic properties than ADP355. After intravenous administration, both JT003 and ADP355 underwent biphasic elimination from the circulation, while the latter concentrations were measurable only for 45 min (Fig. 8k). Compared with ADP355, JT003 possesses longer half-life, larger volume of distribution and higher clearance in SD rats (Supplementary Table 1), which may be attributed to its lipophilic nature. This is also reflected by longer MRT value of JT003. These results clearly showed that JT003 could bind to both AdipoR1 and AdipoR2 with a longer half-life, which indicated that JT003 is a designed, more efficiency dual agonist of AdipoRs. In addition, the half-life of compound is directly related to the pharmacological activities in vivo. Gratifyingly, the enhanced PK profile is a significant contributing factor to the improved in vivo efficacy of JT003 vs ADP355 in accordance with our in vivo results.

## Discussion

As an insulin sensitizer, reduced adiponectin level in vivo was linked to insulin resistance^18^ and inflammatory response^43^. However, adiponectin itself is a relatively larger cytokine which is difficult for drug manufacturers, and the receptors conformational complexity restricts its clinical application either. Thus, adiponectin-based peptides were alternatively studied by different groups. While the beneficial effects of adiponectin signaling on mediating metabolic have been widely accepted^44^, our study provided the evidence of the potential therapeutical effects of an adiponectin-based peptide agonist on NASH and, particularly, the related fibrosis.

The essential roles of adiponectin and its receptors in regulating insulin resistance and lipid metabolism have been well discussed^20,27,45^, and our observation demonstrated that the adiponectin-based peptide agonist JT003 could reduce liver steatosis in mouse models of NAFLD and NASH. After activated by JT003, *AMPK* and *PPARα* expression in liver was increased, indicating that the uptake, β-oxidation and utilization of mitochondrial fatty acid were stimulated^46^. The activation of *PI3K-Akt* signals reduced the expression of *PPARγ*, which exhibits the inhibition of lipogenesis. NASH and related liver fibrosis are largely caused by these metabolic disorders, our studies that the recovering effects of JT003 on the HFD induced NASH and CCl_4_ induced liver fibrosis involved the amelioration on insulin resistance, *PI3K-Akt* signals, HSCs activation as well as ER-mitochondrial axis function (Fig. 8k).

Previous studies implied that the genetic loss of adiponectin increased the activation of HSCs and the liver fibrosis risk^47,48^. In addition, ADP355 has been shown to be effective against liver inflammation and fibrosis in CCl_4_- or thioacetamide-induced mice models, respectively^28,33^. Recently, the active peptide was optimized by docking and the resulting peptide P70 was reported to harbour anti-fibrotic properties^29^. In the present study, collagen content in fibrosis liver as well as fibrosis marker *αSMA* were reduced notably with JT003 treatment, in line with the previous reports^28,29,33^. In addition, mice recovered from liver dysfunction were demonstrated to lower serum ALT/AST and HYP levels. Taken together, we showed that treatment with an adiponectin-based peptide remarkably diminished hepatic fibrosis as well as recovery of liver function in CCl_4_ induced liver fibrosis.

The beneficial effects of JT003 on HSCs activation and lipid metabolism may be partly because of its ability to target ER stress. Treatment of NASH with JT003 reduced the inflammation factors expression remarkably in liver. The ER stress downstream signals, which is linked to steatohepatitis, hepatocyte death and fibrogenesis, was remarkably weakened with the therapy of JT003 after CCl_4_ treated. In this paper, the key genes and the phosphorylation of *PERK* and downstream factors which involve in the ER-associated degradation pathway was notably reduced by JT003 in both NASH and liver fibrosis models, suggesting the ER-mitochondrial axis homeostasis disruption induced apoptosis and DNA damage were lessened significantly. Furthermore, the mitochondrial biogenesis was improved, and mitochondrial function associated genes and proteins which evokes initiation of apoptotic signaling was also reduced after JT003 treating both in vivo and in vitro. These data supported by studies in JT003 treated mice point to the possibility of ER-mitochondrial axis are essential for inhibition of NAFLD progress.

Macrophages are thought to close to collagen production and myofibroblasts formation, as well as HSCs activation, which indisputably play an important role in liver fibrosis^49^. Blockade of macrophages infiltration suppresses activation of HSCs^50^. Our results demonstrated that macrophages activation was suppressed in CCl_4_ induced liver fibrosis mice or activated HSCs with JT003 treatment, with a mechanism we have not figured out. TUNEL assay and mRNA expression detection suggested notably inhibition of apoptosis in fibrotic liver. HSCs activation plays a central role in the aberrant ECM accumulation during liver fibrosis progress^51^. An important finding in our study is the confirmation of the prohibition effect of JT003 on ECM accumulation. A number of cell types and external stimuli converge upon the activation of HSCs. Among fibrogenic mediators, *TGFβ*, *CTGF* and *IL-6* derived from biliary epithelial cells and/or liver progenitor cells were notably reduced by JT003 treatment. *TNFα* and *IL-1β* related apoptosis were also reduced. Mostly, JT003 treatment lessened *NFκB* phosphorylation, indicating the decreasing of harmful cellular stimuli. Overall, the major cause of liver fibrosis, HSCs activation, was notably inhibited with JT003 treatment. Additionally, with AdipoR1 and AdipoR2 expression knockdown in HSCs, the effects of JT003 on *PPARα* and *PPARγ* expression were interdicted. Since HSCs activation is a complex progress, many pathways are involved in it, such as *TGFβ1-Smad2/3* signaling, *Akt-mTOR* pathways and *STAT3* cascades. As a key adipocytokine in liver, adiponectin may also involve in these biological processes. Thus, the mechanism of AdipoR dual agonist JT003 melioration on HSCs activation need to further investigate.

Our results indicate that AdipoRs dual agonist JT003 with a longer half-life could ameliorate NASH and related liver fibrosis via *AMPK*, *PPARα* and *PI3K-Akt* signal pathways. JT003 could notably reverse HSCs activation, a central cause of liver fibrosis, and the improvement of the ER-mitochondrial axis function plays a mechanistic role in the treatment. These findings suggested that the AdipoRs agonists could be a class of valuable candidates for developing effective therapeutics on treating NASH and related fibrosis.

## Methods

### Peptides synthesis

According to the standard method of solid-phase peptide synthesis (SPPS), polypeptide gAd_155 – 161_ and a series of analog (JT001 – JT006) sequences with free N-terminals were synthesized using MBHA amide resin (GL Biochem, Shanghai, China). Purification and atomic accumulation process were reverse phase high performance liquid chromatography mass spectrometric (RP-HPLC-MS, Agilent Technologies, USA). The analysis results are shown in Supplementary Figs. 11 and 12.

### In vitro cytotoxicity assay

The in vitro cytotoxicity of candidate peptides was tested using CCK8 cell proliferation assay kit (Beyotime Biotechnology, Shanghai, China). Cells were seeded in flat bottom 96-well plates (5000 cells per well for LX2) with 100 μL medium per well. Candidate peptides were added to each well to get desired final concentration (16, 32, 64, 128, 256, 512, 1,024 μM). After incubation for 48 h at 37 °C in a humidified atmosphere of 5% CO_2_, 10 μL CCK8 reagent was added to each well and the plate was incubated at 37 °C for another 4 h. At the end of the incubation, the absorbance was read at 490 nm on Flex Station 3 plate reader (Molecular Devices, USA).

### Hemolytic activity assay

The hemolytic activity of candidate peptides was assessed with mouse red blood cells^52^. Candidate peptides were incubated with mouse red blood cells (4% hematocrit in PBS) in indicated concentrations (2, 4, 8, 16, 32, 64, 128, 256, 512, 1,024, 2,048, 4,096 μM) at 37 °C for 1 h. Centrifugation for 10 min at 1000 xg to isolate the supernatants, the absorbance was determined at 490 nm. PBS was used as the positive control and 0.2% Triton X-100 which could cause 100% hemolysis was used as the negative control.

### Serum stability assay

The stability of candidate peptides was investigated following procedure^53^. 5 mM peptides were incubated with mouse serum for 0, 0.5, 1, 1.5, 2, 2.5, 3, 3.5, 4,12 or 24 h at 37 °C. Ice-cold acetonitrile was added to terminate the reaction. Vortex and stand for 1 min, deionized water with 1% TFA was added to the mixture to further terminate the reaction. Mixtures were then centrifuged (13,000*g*, 10 min) to obtain the supernatant. RP-HPLC-MS was applied to analysis the content of candidate peptides. The serum stability of candidate peptides was represented as the retention rate of each peptide.

### Cell confocal imaging

For in vitro receptor binding assay, 4E4 HepG2 cells or 2E4 LX2 cells were seeded on 35 mm glass bottom dish with 150 μL complete medium. Then cells were incubated with FITC labeled peptide for 4 h. The nuclei were stained with Hoechst 33342 (ThermoFisher, Scientific, USA). After washing with PBS, cells were visualized in a laser confocal microscope (Olympus FV3000, Japan). Cells with suppressed AdipoR1 and AdipoR2 expression with each specific siRNA were used as negative control.

### RNA interference

AdipoR1 and AdipoR2 siRNA as well as mock siRNA were chemically synthesized (GenePharma, China) and transfected into 60–70% confluent HepG2 and LX2 cells using Lipofectamine 3000 (ThermoFisher Scientific, USA). Dilution of siRNA and Lipofectamine 3000 reagents as well as the transfection of cells were performed following the manufacturer’s protocol. For HepG2 cell model, 5E5 cells were plated in 6-well plates and transfected with 5 μL of 20 μM stock siRNA. At 48 h following transfection, the cells were processed for 0.25 mM PA and/or 200 μM JT003 treated for another 24 h. BSA was used as the negative control. For LX2 cell model, 2E5 cells were plated in 6-well plates and transfected with 5 μL of 20 μM stock siRNA. At 48 h following transfection, the cells were processed for JT003 or PBS treated for another 48 h. Next, cells were harvested for either western blot or qPCR assay.

### HTRF (homogeneous time resolved fluorescence)

The expression plasmid of AdipoR1 and AdipoR2 were synthesized and codon optimized by Genescript (Nanjing, China) for their expression in HEK293 cell line. The concentration of FITC-JT003 was 0, 0.1, 0.25, 0.5, 1, 2.5 or 5 μM respectively. Unlabeled peptide JT003 was used as the control to measure the non-specific signal. 10E4 AdipoR1 and AdipoR2 overexpression cells, anti-His-Tb (ThermoFisher Scientific, USA) and JT003-FITC were added into a 384-well plate, with a total volume of 25μl. The mixtures were incubated at room temperature for 30 min in the dark. Fluorescent values at 520 nm and 620 nm were measured for K_d_ calculation.

### Competition binding assay

Different concentration of JT003 were incubated with AdipoR1 or AdipoR2 overexpression HEK293 cell line for 4 h. After that, 50 μg ml^−1^ gAd_155–161_ were added for another 4 h incubation. The binding of gAd_155–161_ to the cells were measured via western blot assay.

### Cell model

Hepatoma carcinoma cell line HepG2 and human hepatic activated stellate cell line LX2 used in this study were purchased from Type Culture Collection of the Chinese Academy of Sciences (Shanghai, China). Cells were cultured in Dulbecco’s modified Eagle’s medium (DMEM) with 10% fetal bovine serum (FBS), supplemented with 1% penicillin–streptomycin (ThermoFisher Scientific, USA) at 37 °C in a humidified atmosphere of 5% CO_2_. In PA (Sigma, USA) induced lipid accumulation cell model, HepG2 cells were seeded into a 6-well plate at 5E5 cells per well. 0.25 mM PA and/or 200 μM candidate peptides were co-incubated with cells for 24 h. Then Oil Red O staining was performed to evaluate the accumulation of lipid droplets. BSA (Sigma, USA) was used in this experiment as the negative control. For LX2 cells, 2E5 cells were seeded, then the adherent cells were exposed to 200 μM candidate peptides for another 48 h. Next, the cell protein was obtained and western blotting was performed. Candidate peptides were diluted in DMEM in a stock concentration of 1 M.

### Computational docking

Molecular Operating Environment (MOE) version 2013.8 was used to perform the automated docking studies and ECL-1 was selected as the activity domain. The initial structures of JT003 were pre-calculated a three-dimensional grid in Discovery Studio (DS) version 3.5. Then the structures were energetically minimized with Powell method via default parameters. 20 possible three-dimensional conformations were generated during this phase. Afterward, automated docking calculations were carried out using MOE via default parameters, and the binding free energies of the agonists within the receptors were evaluated. For each possible three-dimensional conformation of JT003, 20 promising docking poses were generated and all docked poses were clustered using a tolerance of 2 Å in positional root-mean-square-deviation (RMSD), and were sorted by binding docking energies. The most favorable free energy of binding was considered as the resultant complex structure.

### Animal models and tissue collection

Male C57BL/6J mice of 6–8 weeks of age (20–24 g) were used in these experiments including the NAFLD model and liver fibrosis model. The mice were purchased from Guangdong Medical Laboratory Animal Center and housed in standard isolator cages and maintained on a room temperature of 25 °C and a 12 h:12 h light/dark cycle. At the end of the experiments, mice were fasted 8 h prior to sacrifice. Liver tissue and/or epididymal adipose tissue were flash frozen in liquid nitrogen (with or without RNAlater (TransGen Biotech, China)) and stored at −80 °C for future use, or fixed in 10% paraformaldehyde for histological assays. Blood samples were obtained from the posterior venous plexus of the eyes of anesthetized mice, Blood was separated by centrifugation at 1000*g* for 15 min Serum was then stored at −80 °C. The ethical approval has been obtained from Animal Care and Use Committee of the Sun Yat-sen University. All animals were maintained in pathogen-free conditions and cared for in accordance with policies and certification of the Association for Assessment and Accreditation of Laboratory Animal Care International (AAALAC International).

The NAFLD model was established in male mice through feeding a HFD continuously for 16 weeks (*n* = 6; protein: 18.1%; fat: 61.6%; carbohydrates: 20.3%; D12492, Research Diets, USA). Mice administered an SD diet serves as control group (*n* = 6; protein: 18.3%; fat: 10.2%; carbohydrates: 71.5%; D12450B, Research Diets, USA). The NASH model was conducted with feeding a HFD for 16 weeks combine with a MCD diet (Methionine/Choline Deficient Diet, Medicience, China) in the last 4 weeks. Mice administered a MCS diet (Methionine/Choline Sufficient Diet, Medicience, China) serves as control group. NAFLD or NASH mice treated with JT003 for 4 weeks were used to evaluate the pharmacological effects (*n* = 6; 500 μg kg^−1^; daily intraperitoneal injection). Food and water were provided ad libitum. The body weight of mice was examined every 3 days. The diet was renewed every 7 days. JT003 was diluted in PBS. Supplementary Fig. 5 showed the schematic of experiment procedure.

Male mice liver fibrosis was induced by intraperitoneal injection of 20% CCl_4_ (Sigma, USA) diluted in corn oil (Aladdin, China) every 3 days for 6 weeks (*n* = 6; 5 ml kg^−1^ body weight). Mice injected with equivalent volume of corn oil serves as control group (*n* = 6). Liver fibrosis mice treated with JT003 for 3 weeks were used to evaluated the pharmacological effects (*n* = 6; 500 μg kg^−1^; daily intraperitoneal injection). Supplementary Fig 19 showed the schematic of experiment procedure.

### OGTT and ITT

Fasting blood glucose was measured using OneTouch UltraVue Blood Glucose Meter and test strips (Johnson&Johnson, USA) from tail nicks. After HFD fed for 14 weeks and JT003 treated for 2 weeks, oral glucose tolerance tests were performed on 8 h fasted mice. 0.5 g ml^−1^ d-glucose solution (Sigma-Aldrich, USA) were oral injected to mice (2 g kg^−1^ of bodyweight), blood glucose level was recorded at baseline, 15, 30, 60, 120 min

For insulin tolerance test, one week after OGTT assay, 6 h fasted mice were treated with JT003, and the baseline blood glucose level was measured. An hour later, 0.25 U kg^−1^ insulin (Novolin R, Denmark) were intraperitoneal injection to mice, blood glucose level was measured at 15, 30, 45, 60 min

### Serum biochemical assays

The levels of ALT, AST, TC and TG in serum were determined using certain commercial assay kits (Nanjing Jiancheng Bioengineering Institute, China). Fasting adiponectin and insulin were quantified in plasma samples using corresponding commercially available Elisa kits (Cloud-Clone Crop, USA).

### Histological examination

The levels of TG and HYP in liver tissue were detected with certain commercial assay kit (Nanjing Jiancheng Bioengineering Institute, China). Formalin-fixed liver tissues were embedded in paraffin, sectioned into 5 μm slices. After deparaffinization and rehydration of the section slices, they were stained with Haematoxylin and Eosin or Periodic Acid-Schiff. For the detection of liver collagen content, Sirius Red staining for collagen was performed. OCT-embedded frozen liver tissues were sectioned into 12 μm slices, and Oil Red O staining was performed. The cryostat-cut slices were stained with freshly prepared Oil Red O working solution for 7 min Two independent experimenters who were blinded to the treatment evaluated the staining slides and analyzed the score for steatosis, glucogen accumulation and collagen proliferation area.

### Immunohistochemistry

Paraffin-embedded liver slices were deparaffinized and rehydrated, antigen retrieval was performed under the high pressure and temperature in 0.01 M sodium citrate buffer (pH = 6.0). To quench the endogenous peroxidase activity, the slices were then incubated with 1% hydrogen peroxide in methanol for 10 min in room temperature. Then slices were incubated with corresponding primary antibodies. After washing, HRP-conjugated secondary antibodies specific to the species of the primary antibodies were used. After the section were developed with DAB, hematoxylin staining was performed for cell counting.

### Apoptosis assay

TUNEL assay was performed using 5 μm liver sections and the apoptotic cells were identified using a Cell Death Detection kit (Beyotime Biotechnology, Shanghai, China). And then the slices were counterstained with DAPI (Life science, ThermoFisher Scientific, USA). Six random fields from 6 slides per mice were examined, and the TUNEL-positive red area within the slides were counted by two individuals who were blinded to the treatment.

### RNA isolation and gene expression assay

Liver total RNA was extracted by using Trizol reagent following the manufacture’s instruction (TransGen Biotech, China). The concentration of RNA was measured by Nanodrop 2000 (ThermoFisher Scientific, USA). cDNA was transcribed from 10ug total RNA with First Strand cDNA synthesis Super Mix (TransGen Biotech, China). The cDNA was diluted 1/50 and 1.0 uL were used as a template for qPCR. qPCR was performed with SYBR Green qPCR Super Mix (TransGen Biotech, China) in the Light Cycler 480 Real-Time PCR System (Roche, Swiss). Reference gene β-actin was used for data normalization. mRNA expression was analyzed with specific primers listed in Supplementary Table 2.

### Protein prepared and western blot analysis

Total protein was isolated from cells or liver samples using RIPA lysis buffer (50.0 mM Tris-HCl (pH = 8.0), 150.0 mM NaCl, 0.02% NaN_3_, 0.1% SDS, 1% NP-40, 0.5% deoxysodium cholate, 1 mM EDTA, 1 μM RIPA). The protein concentration was quantified with BCA kit (Pierce, ThermoFisher Scientific, USA). Thirty micrograms protein were separated by 10 or 12% sodium dodecyl sulfate-polyacrylamide gel electrophoresis depending on the band of target protein, then transferred onto equilibrated polyvinylidene difluoride membrane (PVDF, Immobilon®-PSQ transfer membranes, Merck Millipore, Germany). Membranes were incubated with primary antibodies overnight at 4 °C. Specific antibodies were listed in Supplementary Table 3. After incubation with the secondary antibody, proteins were detected by enhanced chemiluminescence (Glarity^TM^ Western ECL Substrate, Bio-Rad, USA) and the signals were recorded and quantified with Tanon-Image Software (Shanghai, China). Reference antibody GAPDH were used for data normalization.

### Mitochondrial content assay

60–70% confluent LX2 cells and PA induced HepG2 cells were washed with PBS twice and incubated with 100 nM MitoTracker Green FM (YeSan, China) at 37 °C for 30 min For fluorescence intensity assay, cells were harvested by using trypsin/EDTA (ThermoFisher Scientific, USA) and resuspended in PBS. The excitation and emission wavelengths were 490 and 516 nm, respectively, and the intensity values corrected for total protein level (mg ml^−1^). For mitochondrial visualized assay, after incubated with MitoTracker Green FM, the adherent cells were washed with PBS and a single in-focus optical section was acquired with confocal microscopy (Olympus, Japan). The nucleus was stained with Hochest33342 (ThermoFisher Scientific, USA). For every section *n* = 50 cells, images were analyzed and quantified by ImageJ.

### Pharmacokinetic assay

Male SpragueDawley (SD) rats were randomly divided into two groups (*n* = 3 per group) and received a single intravenous dose of JT003 (0.5 mg/kg) or ADP355 (1.0 mg kg^−1^) respectively. Blood samples were collected at 5, 15, 30, 45 min and 1, 2, 3 h following the injection, and then were immediately centrifuged (4000*g*, 10 min, 4 °C) to obtain plasma samples. Plasma concentrations of the two peptides were measured by LC-MS/MS (ThermoFisher Scientific, USA). Pharmacokinetic parameters of JT003 and ADP355 were calculated using non-compartmental analysis in Phoenix WinNonlin (version 8.0, Pharsight, USA). The concentration values less than the LLOQ were set to zero for the analysis.

### Statistical analysis

The positive area of all stained liver slices was quantified by ImageJ (https://imagej.nih.gov/ij/). All data in this study were shown as the mean ± SEM (Standard Error). Student’s two-tailed t test was used to compare the mean of two groups of samples. All statistical analyses were performed with GraphPad Prism software (https://www.graphpad.com/). Significance was considered when *p* < 0.05. In RNAseq gene expression analysis, false discovery rate <0.01, *q* value <0.001. The confidence level was 95%.

### Reporting summary

Further information on research design is available in the Nature Research Reporting Summary linked to this article.

## Supplementary information

Supplementary Information

Peer Review File

Reporting Summary

## Data Availability

Abbreviations were listed in Supplementary Table 4. All relevant data are available from the corresponding author upon reasonable request. All the data supporting the findings of this study are available within this article, supplementary information files, and Source Data file (10.6084/m9.figshare.12988835.v2). RNA-sequencing data that support the findings of this study have been deposited in the Gene Expression Omnibus (GEO) under accession code GSE158951. Source data are provided with this paper.

## Source data

Source Data

## References

[1] Wesolowski SR, Kasmi KC, Jonscher KR, Friedman JE (2017). Developmental origins of NAFLD: a womb with a clue. Nat. Rev. Gastroenterol. Hepatol..

[2] Musso G, Cassader M, Gambino R (2016). Non-alcoholic steatohepatitis: emerging molecular targets and therapeutic strategies. Nat. Rev. Drug Discov..

[3] Friedman SL (2013). Liver fibrosis in 2012: Convergent pathways that cause hepatic fibrosis in NASH. Nat. Rev. Gastroenterol. Hepatol..

[4] Tilg H, Moschen AR (2010). Evolution of inflammation in nonalcoholic fatty liver disease: the multiple parallel hits hypothesis. Hepatology.

[5] Nassir F, Ibdah JA (2014). Role of mitochondria in nonalcoholic fatty liver disease. Int. J. Mol. Sci..

[6] Baiceanu A, Mesdom P, Lagouge M, Foufelle F (2016). Endoplasmic reticulum proteostasis in hepatic steatosis. Nat. Rev. Endocrinol..

[7] Bennett WL (2011). Comparative effectiveness and safety of medications for type 2 diabetes: an update including new drugs and 2-drug combinations. Ann. Intern. Med..

[8] Neuschwander-Tetri BA (2015). Farnesoid X nuclear receptor ligand obeticholic acid for non-cirrhotic, non-alcoholic steatohepatitis (FLINT): a multicentre, randomised, placebo-controlled trial. Lancet.

[9] Armstrong MJ (2016). Liraglutide safety and efficacy in patients with non-alcoholic steatohepatitis (LEAN): a multicentre, double-blind, randomised, placebo-controlled phase 2 study. Lancet.

[10] Filozof C, Goldstein BJ, Williams RN, Sanyal A (2015). Non-alcoholic steatohepatitis: limited available treatment options but promising drugs in development and recent progress towards a regulatory approval pathway. Drugs.

[11] Ratziu V (2012). A phase 2, randomized, double-blind, placebo-controlled study of GS-9450 in subjects with nonalcoholic steatohepatitis. Hepatology.

[12] Loomba R (2018). GS-0976 reduces hepatic steatosis and fibrosis markers in patients with nonalcoholic fatty liver disease. Gastroenterology.

[13] Scherer PE, Williams S, Fogliano M, Baldini G, Lodish HF (1995). A novel serum protein similar to C1q, produced exclusively in adipocytes. J. Biol. Chem..

[14] Fruebis J (2001). Proteolytic cleavage product of 30-kDa adipocyte complement-related protein increases fatty acid oxidation in muscle and causes weight loss in mice. Proc. Natl Acad. Sci. USA.

[15] Yamauchi T (2003). Cloning of adiponectin receptors that mediate antidiabetic metabolic effects. Nature.

[16] Tanabe H (2015). Crystal structures of the human adiponectin receptors. Nature.

[17] Vasiliauskaité-Brooks I (2017). Structural insights into adiponectin receptors suggest ceramidase activity. Nature.

[18] Yamauchi T (2001). The fat-derived hormone adiponectin reverses insulin resistance associated with both lipoatrophy and obesity. Nat. Med..

[19] Berg AH, Combs TP, Du X, Brownlee M, Scherer PE (2001). The adipocyte-secreted protein Acrp30 enhances hepatic insulin action. Nat. Med..

[20] Yamauchi T (2007). Targeted disruption of AdipoR1 and AdipoR2 causes abrogation of adiponectin binding and metabolic actions. Nat. Med..

[21] Iwabu M (2010). Adiponectin and AdipoR1 regulate PGC-1alpha and mitochondria by Ca(2 + ) and AMPK/SIRT1. Nature.

[22] Tomita K (2008). Hepatic AdipoR2 signaling plays a protective role against progression of nonalcoholic steatohepatitis in mice. Hepatology.

[23] Herzig S, Shaw RJ (2017). AMPK: guardian of metabolism and mitochondrial homeostasis. Nat. Rev. Mol. Cell Biol..

[24] Pawlak M, Lefebvre P, Staels B (2015). Molecular mechanism of PPARalpha action and its impact on lipid metabolism, inflammation and fibrosis in non-alcoholic fatty liver disease. J. Hepatol..

[25] Chou IP, Lin YY, Ding ST, Chen CY (2014). Adiponectin receptor 1 enhances fatty acid metabolism and cell survival in palmitate-treated HepG2 cells through the PI3 K/AKT pathway. Eur. J. Nutr..

[26] Matsuda S, Kobayashi M, Kitagishi Y (2013). Roles for PI3K/AKT/PTEN pathway in cell signaling of nonalcoholic fatty liver disease. ISRN Endocrinol..

[27] Okada-Iwabu M (2013). A small-molecule AdipoR agonist for type 2 diabetes and short life in obesity. Nature.

[28] Wang H (2016). Adiponectin-derived active peptide ADP355 exerts anti-inflammatory and anti-fibrotic activities in thioacetamide-induced liver injury. Sci. Rep..

[29] Ma L (2017). A potent peptide as adiponectin receptor 1 agonist to against fibrosis. J. Enzym. Inhib. Med. Chem..

[30] Min X (2012). Crystal structure of a single-chain trimer of human adiponectin globular domain. FEBS Lett..

[31] Otvos L (2011). Design and development of a peptide-based adiponectin receptor agonist for cancer treatment. BMC Biotechnol.

[32] Otvos L (2014). Development of second generation peptides modulating cellular adiponectin receptor responses. Front Chem..

[33] Kumar P (2014). Adiponectin agonist ADP355 attenuates CCl4-induced liver fibrosis in mice. PLoS ONE.

[34] Titchenell PM, Lazar MA, Birnbaum MJ (2017). Unraveling the regulation of hepatic metabolism by insulin. Trends Endocrinol. Metab..

[35] Rines AK, Sharabi K, Tavares CDJ, Puigserver P (2016). Targeting hepatic glucose metabolism in the treatment of type 2 diabetes. Nat. Rev. Drug Discov..

[36] Pollak M (2012). The insulin and insulin-like growth factor receptor family in neoplasia: an update. Nat. Rev. Cancer.

[37] Filadi R, Theurey P, Pizzo P (2017). The endoplasmic reticulum-mitochondria coupling in health and disease: molecules, functions and significance. Cell Calcium.

[38] Dasarathy S (2014). Is the adiponectin-AMPK-mitochondrial axis involved in progression of nonalcoholic fatty liver disease?. Hepatology.

[39] Handa P (2014). Reduced adiponectin signaling due to weight gain results in nonalcoholic steatohepatitis through impaired mitochondrial biogenesis. Hepatology.

[40] Verfaillie T (2012). PERK is required at the ER-mitochondrial contact sites to convey apoptosis after ROS-based ER stress. Cell Death Differ..

[41] Koyama Y, Brenner DA (2017). Liver inflammation and fibrosis. J. Clin. Invest..

[42] Tsuchida T, Friedman SL (2017). Mechanisms of hepatic stellate cell activation. Nat. Rev. Gastroenterol. Hepatol..

[43] Ohashi K (2010). Adiponectin promotes macrophage polarization toward an anti-inflammatory phenotype. J. Biol. Chem..

[44] Yamauchi T, Kadowaki T (2013). Adiponectin receptor as a key player in healthy longevity and obesity-related diseases. Cell Metab..

[45] Wijesekara N (2010). Adiponectin-induced ERK and Akt phosphorylation protects against pancreatic beta cell apoptosis and increases insulin gene expression and secretion. J. Biol. Chem..

[46] Kersten S (2014). Integrated physiology and systems biology of PPARalpha. Mol. Metab..

[47] Saxena NK, Anania FA (2015). Adipocytokines and hepatic fibrosis. Trends Endocrinol. Metab..

[48] Kamada Y (2003). Enhanced carbon tetrachloride-induced liver fibrosis in mice lacking adiponectin. Gastroenterology.

[49] Zhao Q (2018). Rapeseed protein-derived antioxidant peptide RAP ameliorates nonalcoholic steatohepatitis and related metabolic disorders in mice. Mol. Pharm..

[50] Imamura M, Ogawa T, Sasaguri Y, Chayama K, Ueno H (2005). Suppression of macrophage infiltration inhibits activation of hepatic stellate cells and liver fibrogenesis in rats. Gastroenterology.

[51] Leiva O (2017). The role of the extracellular matrix in primary myelofibrosis. Blood Cancer J..

[52] Xie J (2015). Antimicrobial activities and action mechanism studies of transportan 10 and its analogues against multidrug-resistant bacteria. J. Pept. Sci..

[53] Xie J (2017). Novel antimicrobial peptide CPF-C1 analogs with superior stabilities and activities against multidrug-resistant bacteria. Chem. Biol. Drug Des..

