# Author Correction: AdipoR1/AdipoR2 dual agonist recovers nonalcoholic steatohepatitis and related fibrosis via endoplasmic reticulum-mitochondria axis

**DOI:** 10.1038/s41467-021-22414-7

**Published:** 2021-03-26

**Authors:** Hongjiao Xu, Qian Zhao, Nazi Song, Zhibin Yan, Runfeng Lin, Shuohan Wu, Lili Jiang, Sihua Hong, Junqiu Xie, Huihao Zhou, Rui Wang, Xianxing Jiang

**Affiliations:** 1 School of Pharmaceutical Sciences, Sun Yat-Sen University, 132 East Outer Ring Road, Guangzhou, 510006 China; 2 Key Laboratory of Preclinical Study for New Drugs of Gansu Province, School of Basic Medical Sciences, Lanzhou University, Lanzhou, 730000 China

**Keywords:** Structure-based drug design, Pharmacology, Non-alcoholic steatohepatitis, Drug development

Correction to: *Nature Communications* 10.1038/s41467-020-19668-y, published online 16 November 2020.

The original version of this Article omitted references to previous work mapping the adiponectin active site and describing the development of the adiponectin receptor agonist ADP355 (Otvos et al. *BMC Biotechnol.* **11**, 90 (2011). 10.1186/1472-6750-11-90) used as a control in this Article and reporting anti-fibrotic activity of ADP355 (Otvos et al. *Front. Chem*. **2**, 93 (2014). 10.3389/fchem.2014.00093 and Kumar et al. *PLoS ONE* **9**, e110405 (2014). 10.1371/journal.pone.0110405). ADP355 was added as a control compound during the peer review process, and corresponding modifications to reference and discuss the original research on ADP355 in the context of this Article in the main text were inadvertently omitted.

These references have been added as new references 31, 32, 33. To discuss these references in the context of this Article, the following four sections have been corrected:

1. The first paragraph of the “Results” section, which originally read “Previous peptide mapping has identified the gAd sequence between Lys149 and Val166 as the adiponectin active site^30^. The following study showed that a shorter heptapeptide derived from the active site of adiponectin is sufficient to activate AdipoR1 efficiently^29^”. The corrected version replaces this section with “Previous peptide mapping has identified the gAd sequence between Lys149 and Val166 as the adiponectin active site^30,31^, and led to the development of ADP355, the first adiponectin receptor peptide-based agonist acting on both AdipoR1 and AdipoR2^31^. In addition, a following study showed that a shorter heptapeptide derived from the active site of adiponectin, P70, is sufficient to activate AdipoR1 efficiently and demonstrated anti-fibrotic activity^29^”.

2. The second paragraph of the “Results” section, which originally read “To evaluate the anti-lipid accumulation and anti-fibrogenic effects of these analogs, PA induced HepG2 cell model and human HSCs derived LX2 cell model were employed”. The corrected version replaces this section with “To evaluate the anti-lipid accumulation and anti-fibrogenic effects of these analogs, a PA induced HepG2 cell model and a human HSCs derived LX2 cell model were employed. ADP355, which shares sequence similarity with JT003 and has exhibited anti-fibrotic activity^28,31–33^ was used as a control in these and subsequent experiments”.

3. The second paragraph of the “Discussion” section, which originally read “The essential roles of adiponectin and its receptors in regulating insulin resistance and lipid metabolism have been well discussed^20,27,42^, and our observation further confirmed that the adiponectin-based peptide agonist could regulating the related metabolic disorders”. The corrected version replaces this section with “The essential roles of adiponectin and its receptors in regulating insulin resistance and lipid metabolism have been well discussed^20,27,45^, and our observation demonstrated that the adiponectin-based peptide agonist JT003 could reduce liver steatosis in mouse models of NAFLD and NASH”.

4. The second paragraph of the “Discussion” section, which originally read “Previous studies implied that the lack of adiponectin increased the activation of HSCs and the liver fibrosis risk^44,45^. In the present study, collagen content in fibrosis liver as well as fibrosis marker αSMA were reduced notably with JT003 treatment”. The corrected version replaces this section with “Previous studies implied that the genetic loss of adiponectin increased the activation of HSCs and the liver fibrosis risk^47,48^. In addition, ADP355 has been shown to be effective against liver inflammation and fibrosis in CCl_4_- or thioacetamide-induced mice models, respectively^28,33^. Recently, the active peptide was optimized by docking and the resulting peptide P70 was reported to harbour anti-fibrotic properties^29^. In the present study, collagen content in fibrosis liver as well as fibrosis marker *αSMA* were reduced notably with JT003 treatment, in line with the previous reports^28,29,33^”.

These sections have been corrected in both the PDF and HTML versions of the Article.

