# Supplementary material for: AdipoR1/AdipoR2 dual agonist recovers nonalcoholic steatohepatitis and related fibrosis via endoplasmic reticulum-mitochondria axis
Source: Nat Commun. 2020 Nov 16;11:5807. doi: 10.1038/s41467-020-19668-y (PMC7669869; doi:10.1038/s41467-020-19668-y)
Supplement: Supplementary file 1 — Supplementary Information [file 41467_2020_19668_MOESM1_ESM.pdf]

Supplementary Information

AdipoR1/AdipoR2 dual agonist recovers nonalcoholic steatohepatitis and related fibrosis via endoplasmic reticulum-mitochondria axis

Xu et al.

Supplementary materials include:

Supplementary Figures 1 to 18

Supplementary Tables 1 to 4

Supplementary Figures

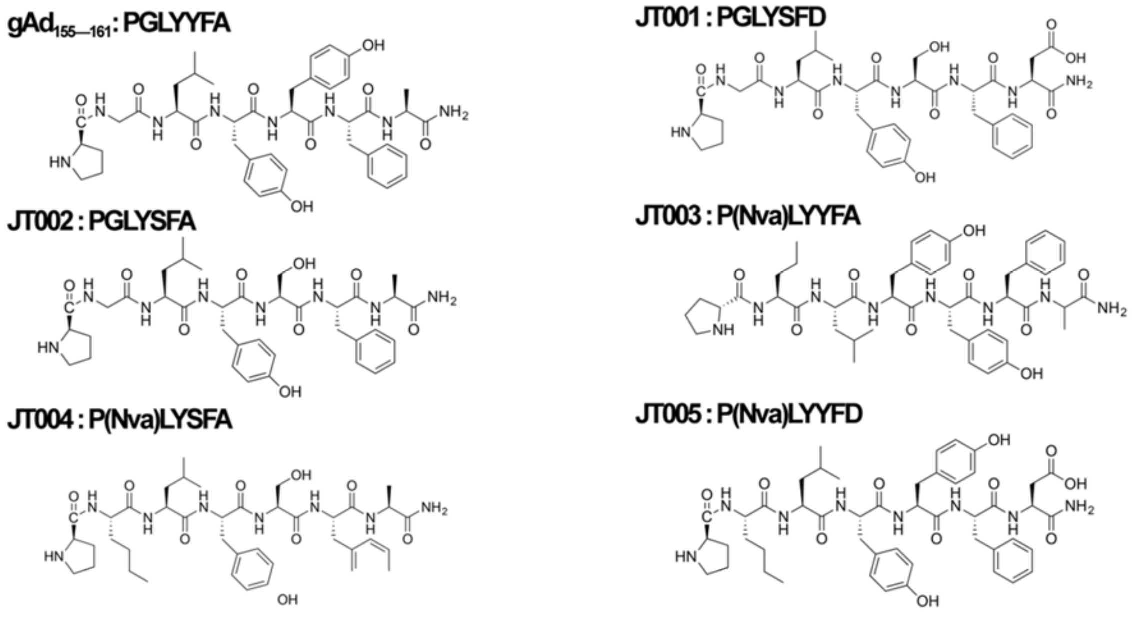

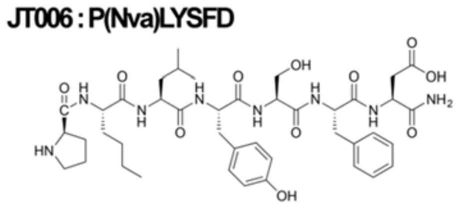

AdipoR Agonist	Sequence	Molecular Weight	Hydrophobic Ratio	Total Net Charge	Retention Time
$gAd_{155-161}$	PGLYYFA	829.4241	42%	0	11.324
JT001	PGLYSFD	797.3830	28%	-1	10.212
JT002	PGLYSFA	753.3935	42%	0	10.405
JT003	P{Nva}LYYFA	871.4719	57%	0	12.213
JT004	P{Nva}LYSFA	795.4404	57%	0	11.107
JT005	P{Nva}LYYFD	915.4618	42%	-1	11.888
JT006	P{Nva}LYSFD	839.4306	42%	-1	10.829

Supplementary Figure 1. Sequence information. *Sequence information and physiochemical characteristics of adiponectin- based peptides.*

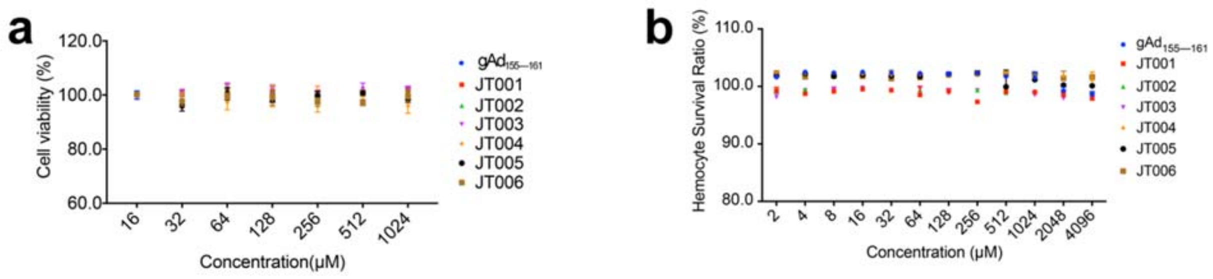

Supplementary Figure 2. Cytotoxicity assay and haemolytic activity assay. *(a) In vitro cytotoxicity CCK8 assay on LX2 cell lines. Data are presented as mean values +/- SEM (n=5/group, unpaired student's t test). (b) The hemolytic activity of the analogues against mouse*

hemocytes. Data were shown as the mean ± SEM. Data are presented as mean values +/- SEM (n=2/group, unpaired student's t test). Source data are provided as a Source Data file.

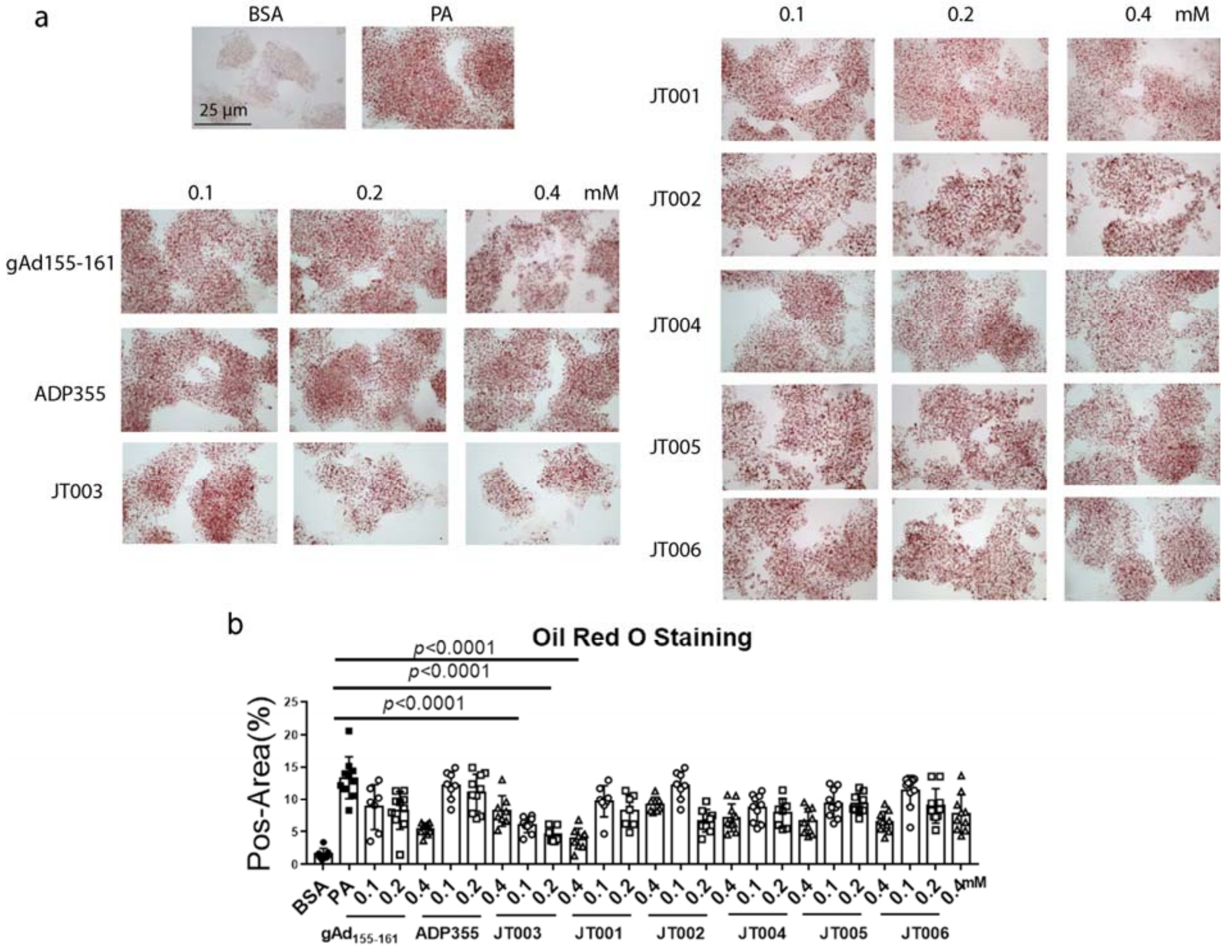

Supplementary Figure 3. Oil red o staining. *(a) Oil red o staining of PA induced lipid accumulation in HepG2 cell line after treated with different concentration of peptides. Triplicates were performed. (b) Positive areas were analyzed with ImageJ. Here and later, unless otherwise indicated, data were shown as the mean ± SEM, statistical significance of the differences between each group was determined by Student's two-tailed t-test. n=4 biologically independent samples. Source data are provided as a Source Data file.*

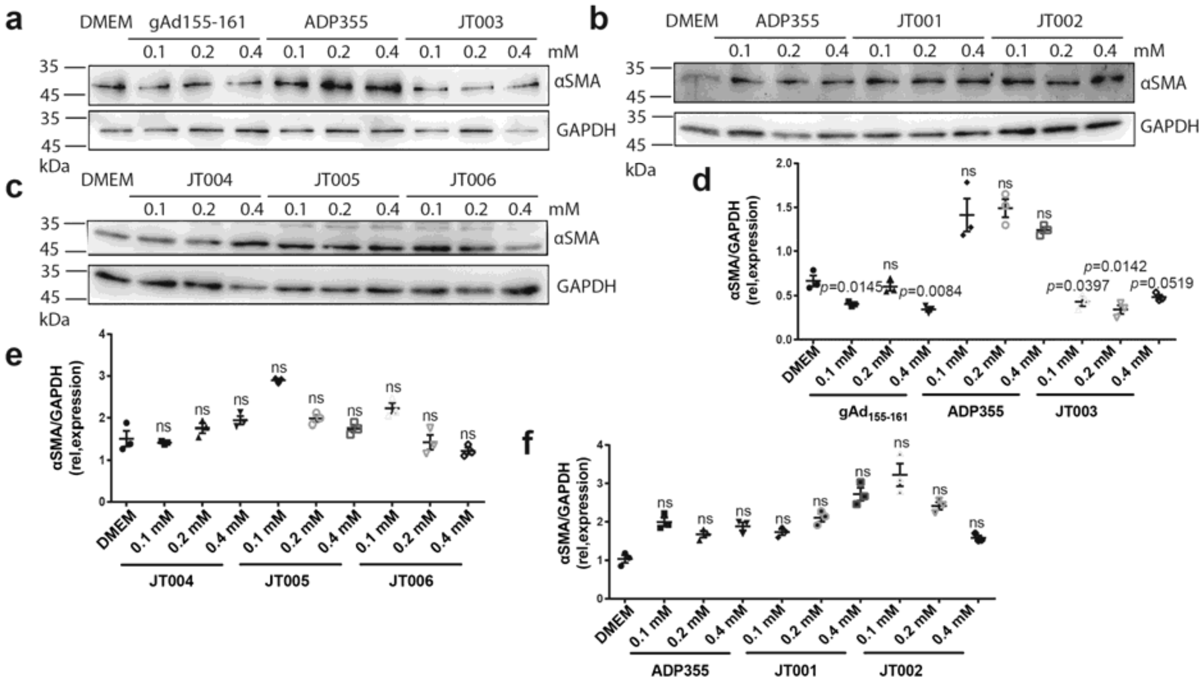

Supplementary Figure 4. Screening of similar peptides. *(a - c) Western blotting for αSMA expression in LX2 after peptides treated. Three separated experiments were performed. (d - f) αSMA expression was normalized to that of GAPDH. All the above data are presented as mean values +/- SEM (n=4, unpaired student's t test). Source data are provided as a Source Data file.*

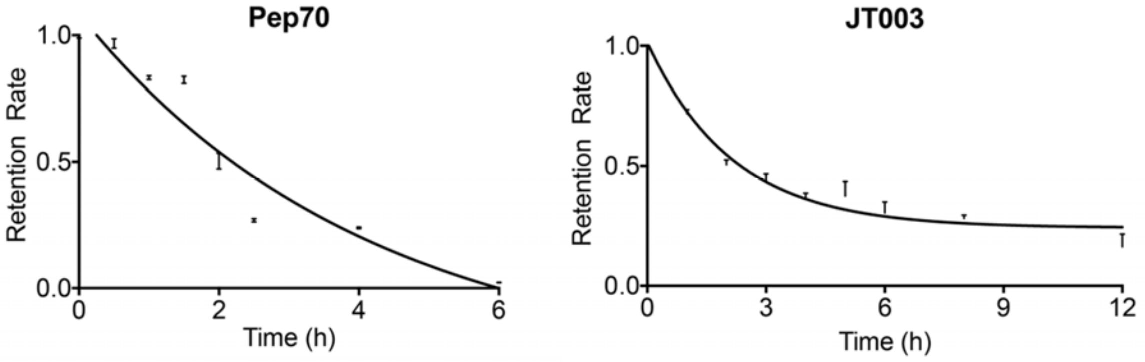

Supplementary Figure 5. Serum stability. *The serum stability of gAd-derived short peptide Pep70 (sequence: PGLYYFD) and JT003, samples were analyzed by RP-HPLC. The experiments were triplicated and the data were shown as the mean ± SEM. Source data are provided as a Source Data file.*

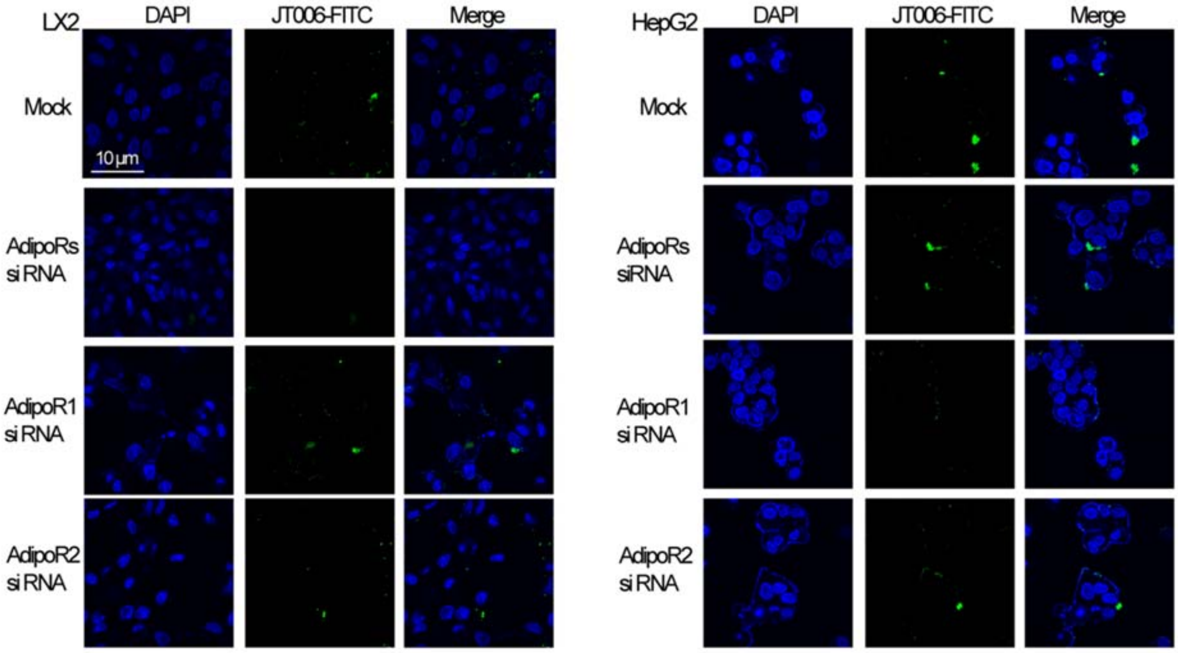

Supplementary Figure 6. Cell confocal imaging. *HepG2 and LX2 cells were incubated with FITC-JT006 at 37 °C for 2 or 4 h. The nucleus was stained with Hochest33342. The green channel represents as FITC. For each experiment, triplicates were performed. For in vitro experiment, the concentration of JT003 is 200 μM.*

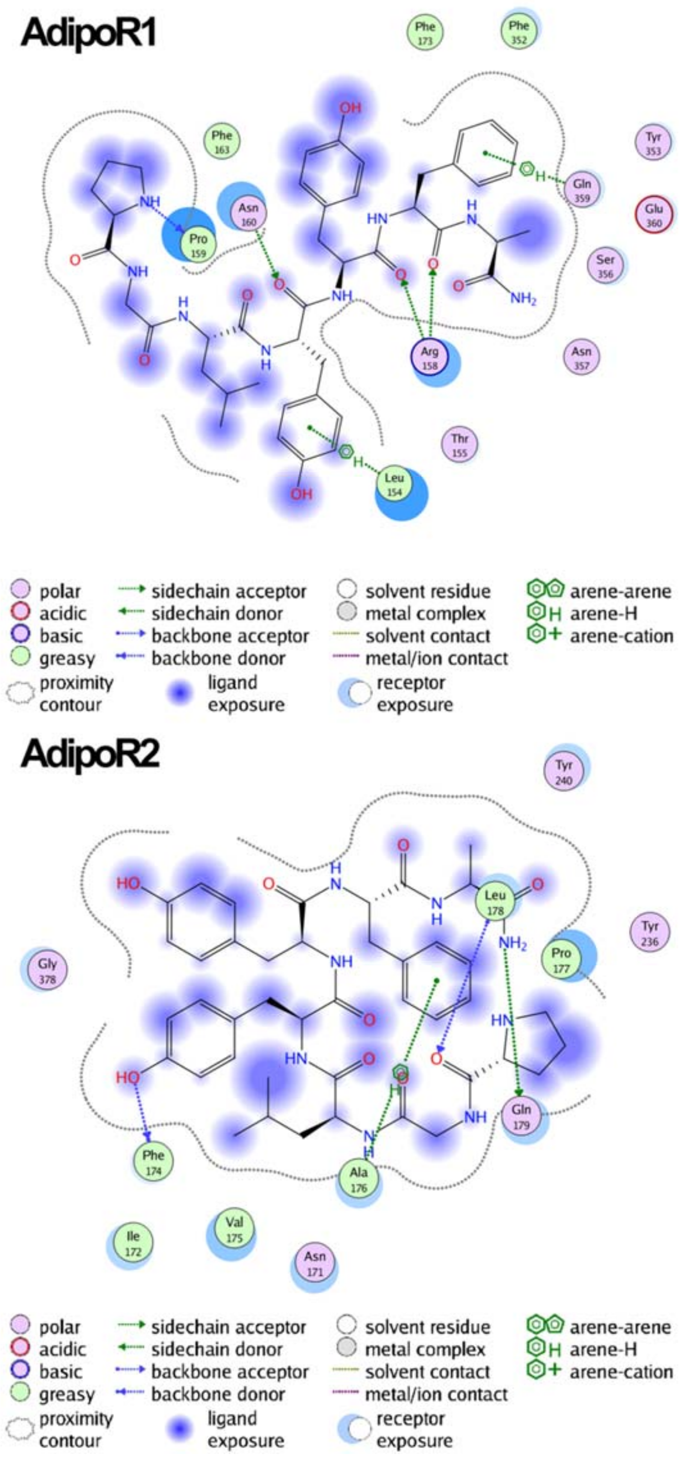

Supplementary Figure 7. Computational docking. *Binding mode of JT003 with AdipoR1 and AdipoR2. Hydrogen-bonding interactions are shown in dash. Peptides docked structure were shown as sticks. Figures were generated from MOE.*

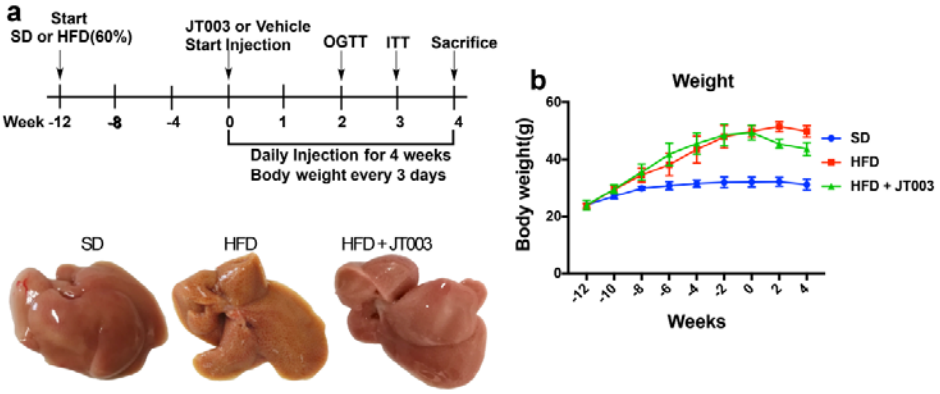

Supplementary Figure 8. HFD mouse data. *(a) Schedule of HFD induced NASH and JT003 therapy. The bottom panel showed the representative photographs of livers. (b) Body weight changes during HFD and JT003 treatment. For the in vivo experiment, the dose of JT003 is 500 μg kg*$^{-1}$*. Data are presented as mean values +/- SEM (n=6/group, unpaired student's t test).*

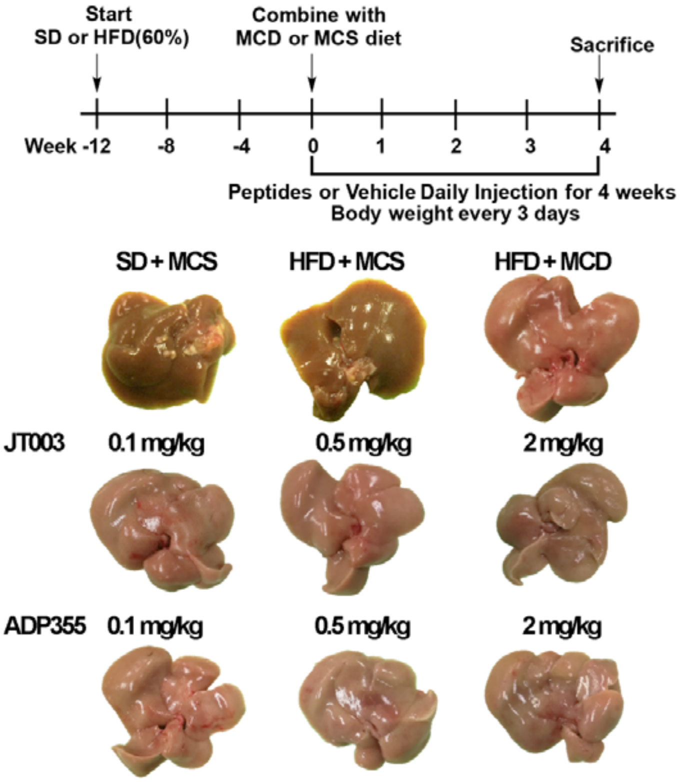

Supplementary Figure 9. MCD mouse data. *Schedule of HFD combine with MCD diet induced NASH and JT003 as well as ADP355 therapy. The bottom panel showed the representative photographs of livers. Here and later, for each assay, three separated experiments were performed. For each experiment, n = 6.*

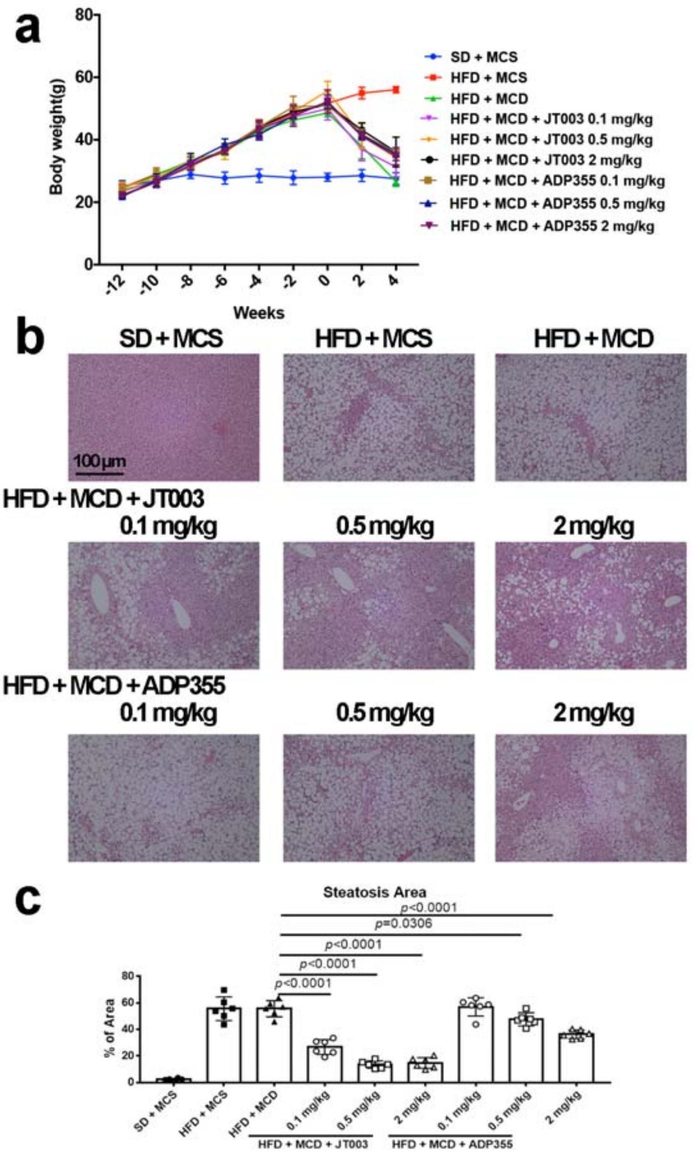

Supplementary Figure 10. HFD+MCD mouse data. *(a) Weight gain of mice treated with SD + MCS, HFD + MCS, HFD + MCD or JT003 as well as ADP355 therapy. Data are presented as mean values +/- SEM (n=6/group, unpaired student's t test). (b, c) Representative images of HE staining of liver sections on mice of indicated groups and steatosis area. Positive areas were analyzed with ImageJ. Data are presented as mean values +/- SEM (n=6/group, unpaired student's t test). Source data are provided as a Source Data file.*

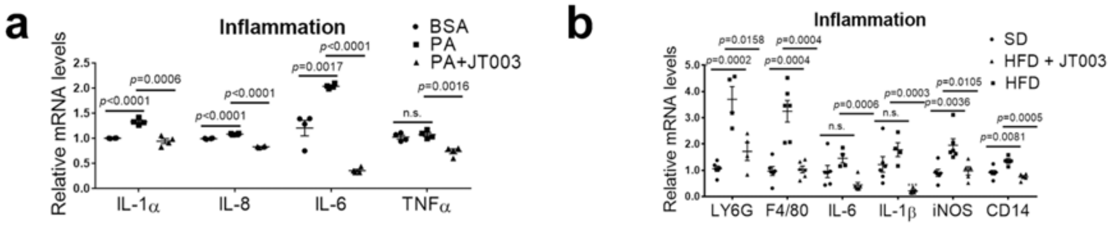

Supplementary Figure 11. Inflammation related data. *The mRNA transcription levels of the genes related to inflammation in NASH cell models (a) and high fat diet induced NASH mice (n=4). (b). Three separated experiments were performed. For the in vivo experiment, the dose of JT003 is 500 μg kg-1 (n=6). All the above data are presented as mean values +/- SEM using unpaired Student's t test. Source data are provided as a Source Data file.*

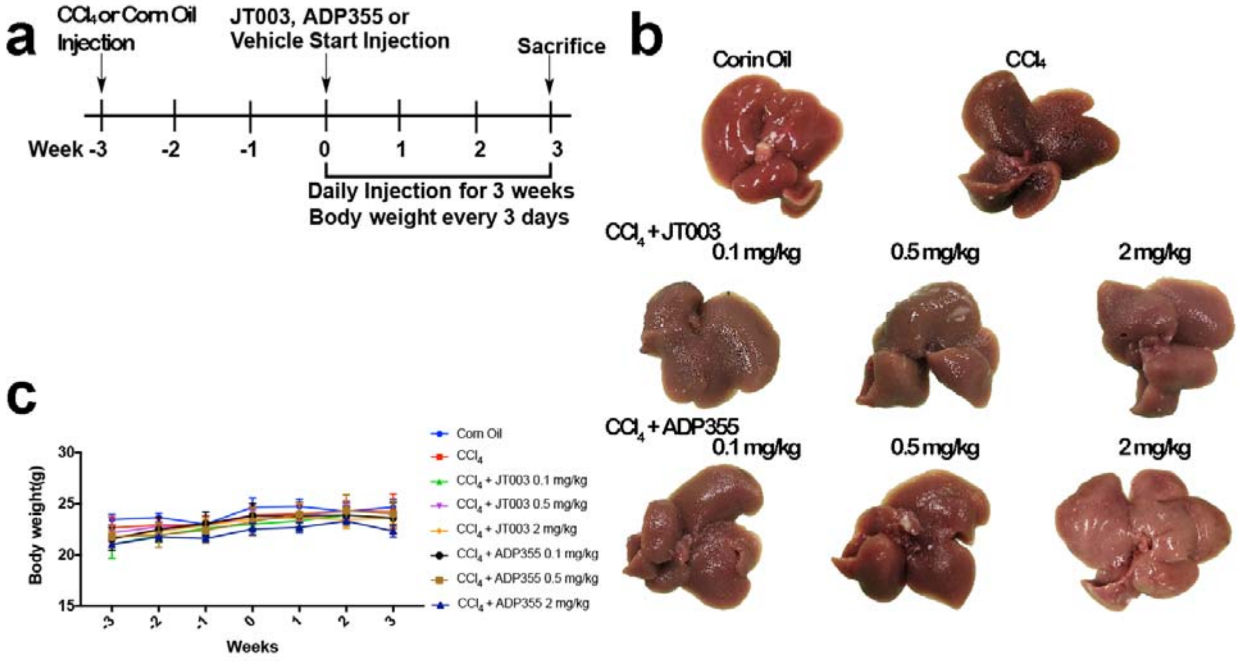

Supplementary Figure 12. CCl_4 mouse data. *(a) Schedule of CCl_4 induced liver fibrosis and JT003 as well as ADP355 therapy. (b) The representative photographs of livers. (c) Body weight changes. Data are presented as mean values +/- SEM (n=6/group).*

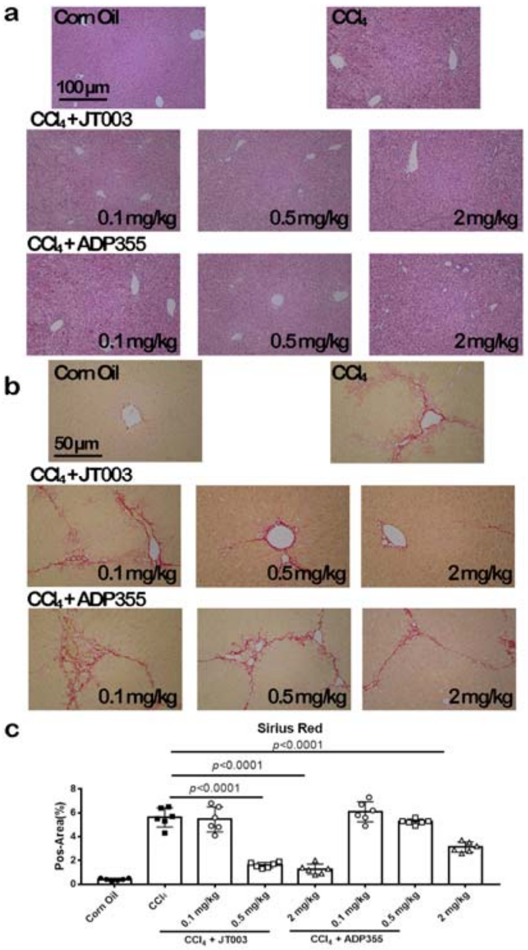

Supplementary Figure 13. HE and Sirius red staining. *Representative images of HE (a) and Sirius red (b) staining of liver sections on mice of indicated groups. (c) Sirius red positive areas were analyzed with ImageJ. Data are presented as mean values +/- SEM (n=6/group, unpaired student's t test). Source data are provided as a Source Data file.*

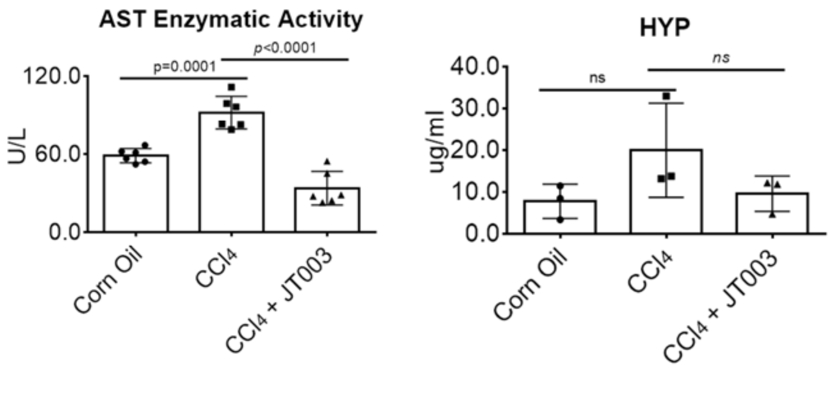

Supplementary Figure 14. Serum biochemical assays. *Serum AST enzyme activities and HYP contents in the liver samples of indicated mice. Three separated experiments were performed. n = 6. The dose of JT003 is 500 μg kg^{-1}. Source data are provided as a Source Data file.*

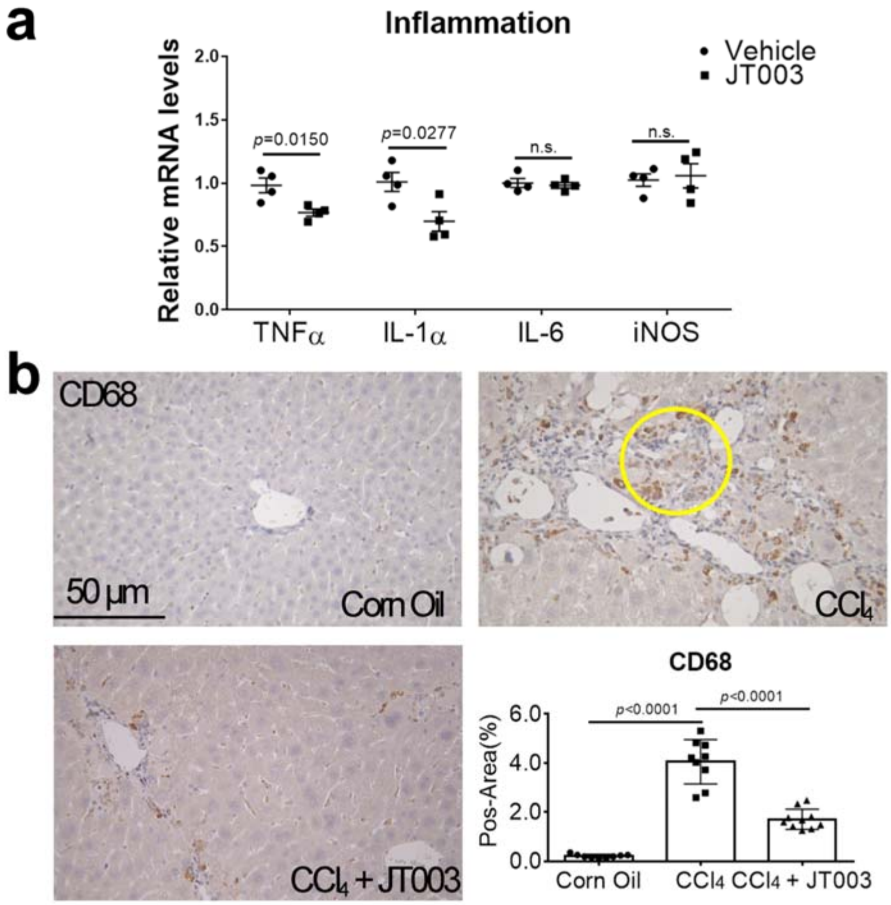

Supplementary Figure 15. Inflammation related data. *(a) The mRNA transcription levels of the genes related to inflammation response in LX2 cells (n = 4, unpaired student's t test). Data are presented as mean values +/- SEM. (b) Representative images of IHC staining for CD68 of liver sections from the mice treated with Corn Oil, CCl_4 or CCl_4 plus JT003 therapy, and positive areas are quantified. Three separated experiments were performed (n = 6, unpaired student's t test). The dose of JT003 is 500 μg kg^{-1}. Data are presented as mean values +/- SEM. Source data are provided as a Source Data file.*

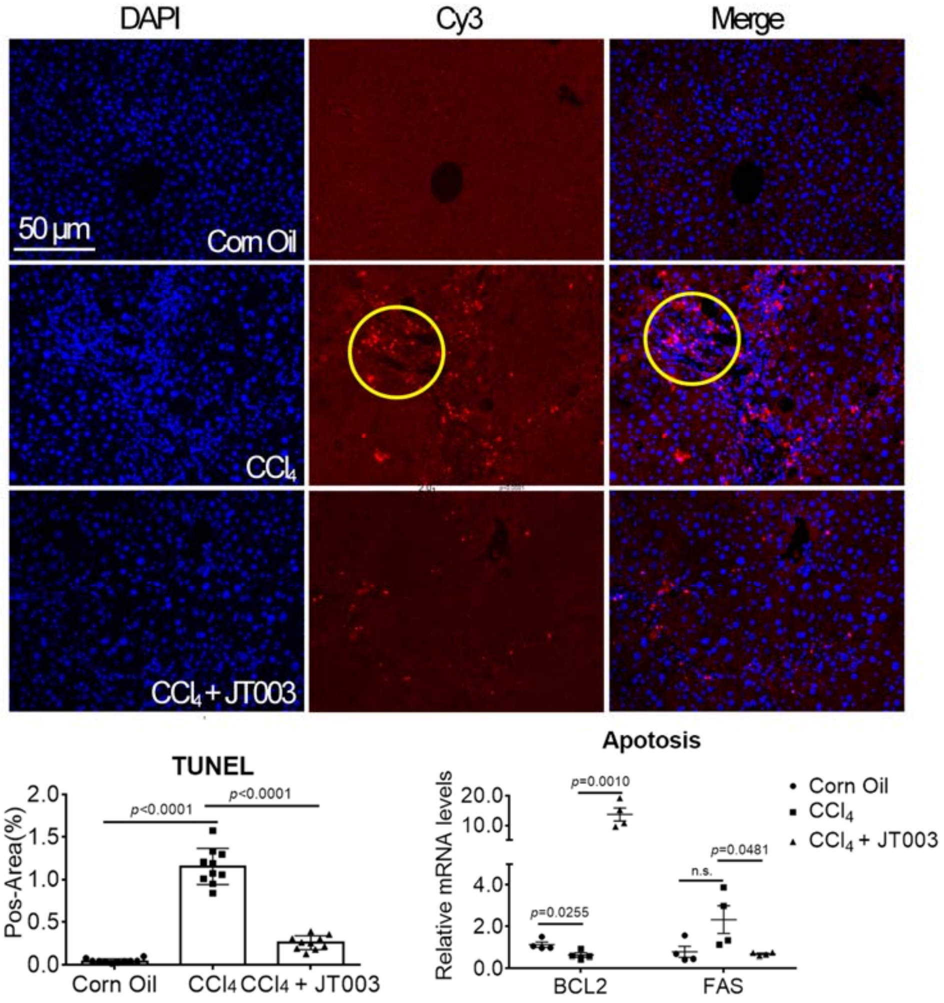

Supplementary Figure 16. Apoptosis data. *Representative images of TUNEL assay in liver sections of the indicated groups (n=6). Positive area was analyzed. The liver regeneration analysis for FAS and BCL2 was performed by Quantitative RT-PCR (n=6). Three separated experiments were performed. The dose of JT003 is 500 µg kg-1. All the above data are presented as mean values +/- SEM using unpaired Student's t test. Source data are provided as a Source Data file.*

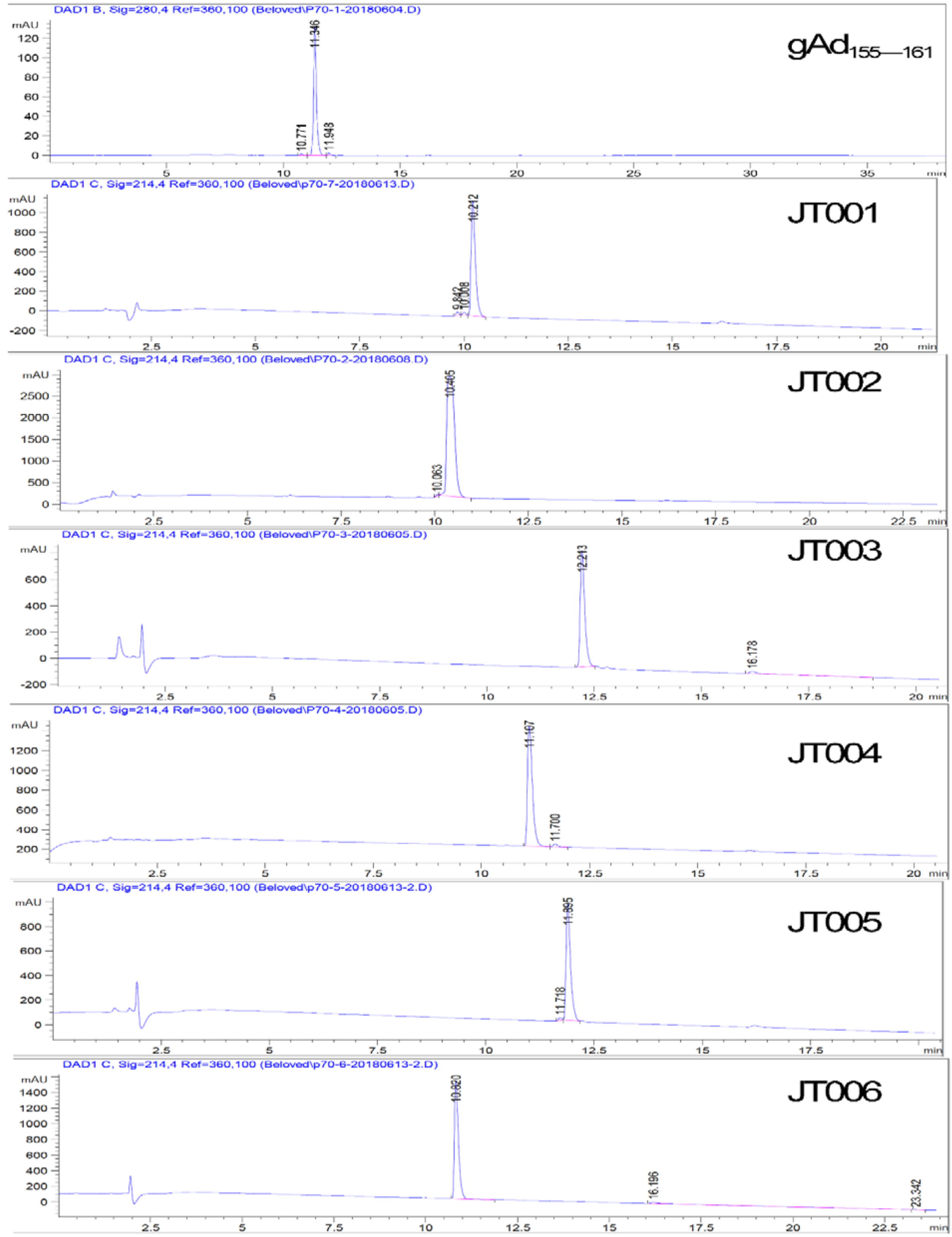

Supplementary Figure 17（continued）:

**$gAd_{155–161}$ Peak**	**Retention time**	**Peak width (min)**	**Peak height (mAU)**	**Area (mAU×s)**	**%Area**
1	10.771	0.1169	1.54647	12.04984	1.0556
2	11.346	0.1241	132.18123	1111.39221	97.361
3	11.948	0.099	2.73766	18.07986	1.5838
**JT001 Peak**	**Retention time**	**Peak width (min)**	**Peak height (mAU)**	**Area (mAU×s)**	**%Area**
1	9.842	0.0754	34.85247	165.6138	1.944
2	10.008	0.0787	28.95564	140.62184	1.6507
3	10.212	0.1057	1173.49158	8212.8125	96.4053
**JT002 Peak**	**Retention time**	**Peak width (min)**	**Peak height (mAU)**	**Area (mAU×s)**	**%Area**
1	10.063	0.0636	30.72016	119.98077	0.3006
2	10.405	0.2335	2771.95947	3.98E+04	99.6994
**JT003 Peak**	**Retention time**	**Peak width (min)**	**Peak height (mAU)**	**Area (mAU×s)**	**%Area**
1	12.213	0.1116	881.73486	6470.67725	95.2872
2	16.178	0.2297	19.27689	320.0307	4.7128
**JT004 Peak**	**Retention time**	**Peak width (min)**	**Peak height (mAU)**	**Area (mAU×s)**	**%Area**
1	11.107	0.1238	1215.7478	9769.6416	97.9969
2	11.7	0.0946	31.22335	199.69106	2.0031
**JT005 Peak**	**Retention time**	**Peak width (min)**	**Peak height (mAU)**	**Area (mAU×s)**	**%Area**
1	11.718	0.0796	21.08563	100.49123	1.4672
2	11.895	0.1093	945.50635	6748.62451	98.5328
**JT006 Peak**	**Retention time**	**Peak width (min)**	**Peak height (mAU)**	**Area (mAU×s)**	**%Area**
1	10.82	0.117	1503.84778	1.15E+04	95.0106
2	16.196	0.4112	18.33101	592.72107	4.9072
3	23.342	0.1336	1.11856	9.93257	0.0822

Supplementary Figure 17. *Purification of candidate peptides with reverse phase high performance liquid chromatography mass spectrometric (RP-HPLC-MS).*

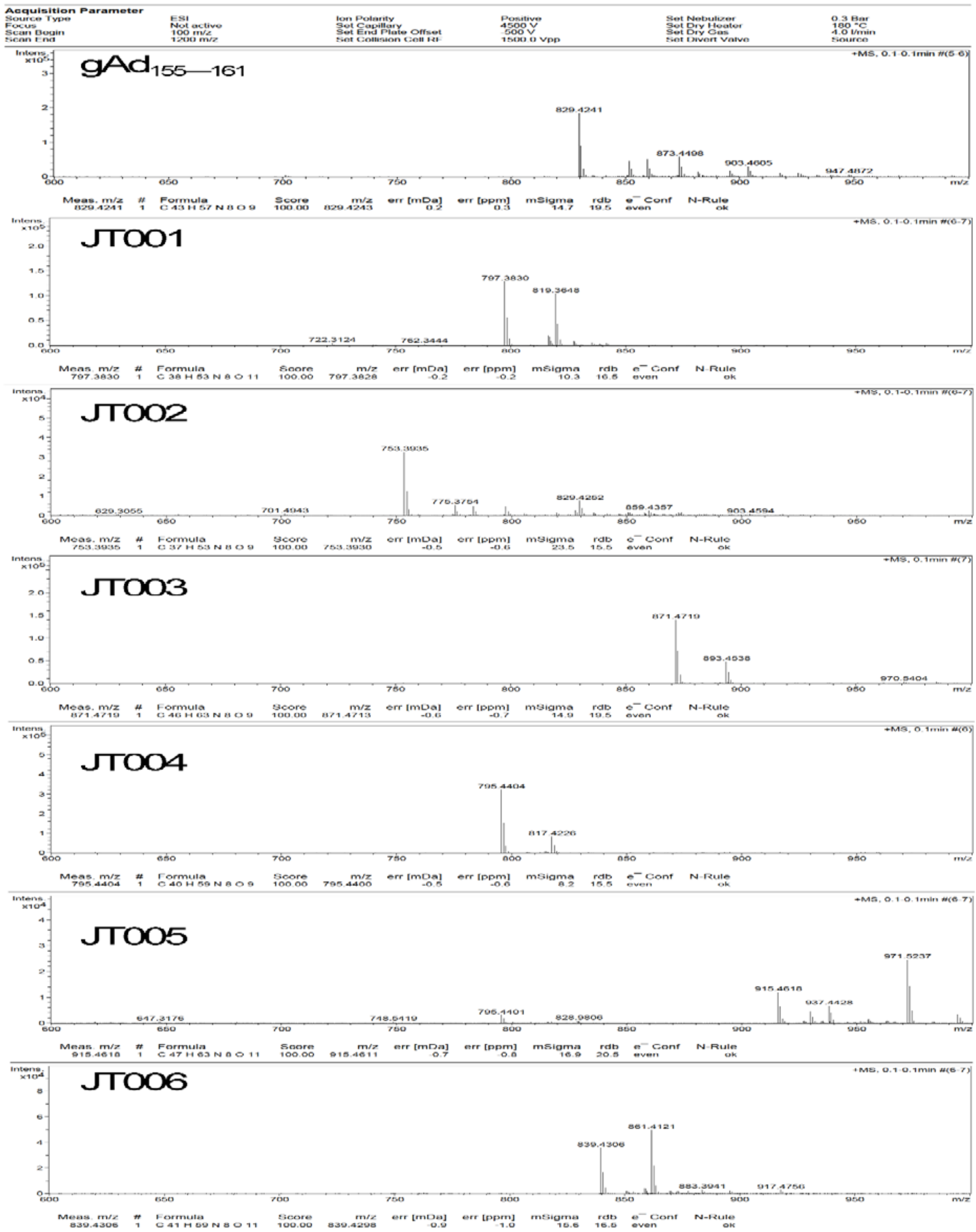

Supplementary Figure 18. *Confirmation of atomic accumulation of candidate peptides with electrospray ionisation-mass spectrometry.*

Supplementary Tables

Parameter	JT003 (iv, 0.5 mg/kg)	ADP355 (iv, 1.0 mg/kg)
$t_{1/2}$ (h)	0.75 ± 0.01	0.10 ± 0.01
AUC_{0-t} (h*ng/mL)	19.36 ± 1.51	134.01 ± 19.87
$AUC_{0\to\infty}$ (h*ng/mL)	20.85 ± 1.63	134.45 ± 19.91
V (L/kg)	25.96 ± 2.29	1.06 ± 0.21
CL (L/h/kg)	24.08 ± 1.92	7.54 ± 1.04
MRT (h)	1.04 ± 0.03	0.09 ± 0.01

Supplementary Table 1. *Pharmacokinetic parameters of JT003 and ADP355 in SD rats. Pharmacokinetic parameters were calculated from plasma concentration-time data using non-compartmental analysis. The results were reported as Mean ± SD for n = 3-6.*

Mouse Gene	Abbreviations	Forward Primer	Reverse Primer
ACC1	Acetyl-CoA carboxylase 1	GATGAACCATCTCCGTTGGC	CCCAATTAT GAATCGGGAGTGC
ACSL3	Long-chain-fatty-acid-CoA ligase 3	GTCAGGGTCCTGAGGAGGT	CCTCACAGCAAGTTCAAGGA
ATF4	Activating transcription factor 4	GGGTTCTGTCTTCCACTCCA	AAGCAGCAGAGTCAGGCTTTC
BAX	Bcl-2-associated X protein	GATCAGCTCGGGCACTTTAG	TTGCTGATGGCAACTTCAAC
BID	BH3 interacting-domain death agonist	GAGATGGAGGCAACCAAACTG	CTGCGCGTTGTACTGATGT
BIM	BCL2 like 11	GCTCCTGTGCAATCCGTATC	GCCCCTACCTCCCTACAGAC
CAT	Catalase	AGCGACCAGATGAAGCAGTG	TCCGCTCTCTGTCAAAGTGTG
CD14	Cluster of differentiation 14	TTTAACTCTGGCGTAGTCACC	GACCCTCAGAAACCAGGAG
CD36	Cluster of differentiation 36	TGGGTTTTGCACATCAAAGA	GATGGACCTGCAAATGTCAGA
CHOP	DNA-damage inducible transcript 3	CCACCACACCTGAAAGCAGAA	AGGTGAAAGGCAGGGACTCA
CPT-1α	Carnitine palmitoyltransferase 1 alpha	AGGACCCTGAGGCATCTATT	ATGACCTCCTGGCATTCTCC
CTGF	Connective tissue growth factor	GCTTGGCGATTTTAGGTGTC	CAGACTGGAGAAGCAGAGCC
CYP2E1	Cytochrome P450 family 2 subfamily E member 1	CTTAGGGAAAACCTCCGCAC	GGGACATTCCTGTGTTCCAG
DGAT1	Diacylglycerol O-acyltransferase 1	ACCTGGCCACAATCATCTG	TGGAGTATGATGCCAGAGCA
F4/80	EGF-like module-containing mucin-like hormone receptor-like 1	CCCCAGTGTCCTTACAGAGTG	GTGCCCAGAGTGGATGTC T
FASN	Fatty acid synthase	TACAGGAGTTCTGGGCCAAC	GACCGCTTGGGTAATCCATA
Gpx1	Glutathione peroxidase 1	AGTCCACCGTGTATGCCTTCT	GAGACGCGACATTCTCAATGA
GRP78	Heat shock protein family A (Hsp70) member 5	TTCAGCCAATTATCAGCAAACTCT	TTTTCTGATGTATCCTCTTCACCAGT
GSTM1	Glutathione S-transferase mu 1	ATACTGGGATACTGGAACGTCC	AGTCAGGGTTGTAACAGAGCAT
HMGCR	3-hydroxy-3-methyl-glutaryl-coenzyme A reductase	GCCCTCAGTTCAAATTCACAG	TTCCACAAGAGCGTCAAGAG
HO-1	Heme oxygenase 1	AAGCCGAGAATGCTGAGTTCA	GCCGTGTAGATATGGTACAAGGA
IL-1β	Interleukin-1 beta	CCGTGGACCTTCCAGGATGA	GGGAACGTCACACACCAGCA
IL-6	Interleukin-6	AGTTGCCTTCTTGGGACTGA	TCCACGATTTCCCAGAGAAC
iNOS	Inducible isoform Nitric oxide synthases	TGCGCCTTTGCTCA TGACA TCGA	ATGGATGCTGCTGAGGGCTCTGTT
LCAD	Long-chain acyl-CoA	GGAGTAAGAACGAACGCCAA	GCCACGACGATCACGAGAT
LY6G	Lymphocyte antigen 6 complex	GGCTCAGAAAAGTGCACCA	CGTACGTGGAAGCGAACAG
MCAD	Medium-chain acyl-CoA	TGGCGTATGGGTGTACAGGG	CCAAATACTTCTTTTTTTGTTGATCA
MMP13	Matrix metallopeptidase 13	GGTCCTTGGAGTGATCCAGA	TGATGAAACCTGGACAAGCA
MMP2	Matrix metallopeptidase 2	CCAGAAGGCGAACAGACTG	TGGGCCGGAGACCTAAAGAG
MMP3	Matrix metallopeptidase 3	AGCCTTGGCTGAGTGGTAGA	CGATGATGAACGATGGACAG
MMP8	Matrix metallopeptidase 8	AGACCGGAATTGATTGCTTG	CCCAGTACCTGAACACCTGGA
MMP9	Matrix metallopeptidase 9	GCGCCACCACAGCCAACTATG	TGGATGCCGTCTATGTCGTCTTTA

NQO1	NAD(P)H dehydrogenase (quinone 1)	AGCGTTCGGTATTACGATCC	AGTACAATCAGGGCTCTTCTCG
PGC-1α	Peroxisome proliferative activated receptor, gamma, coactivator 1 alpha	TATGGAGTGACATAGAGTGTGCT	CCACTTCAATCCACCCAGAAAG
PGC-1β	Peroxisome proliferative activated receptor, gamma, coactivator 1 beta	TCCTGTAAAAGCCCGGAGTAT	GCTCTGGTAGGGGCAGTGA
PPARγ	Peroxisome proliferator activated receptor gamma	ATTCTGGCCCACCAACTTCGG	TGGAAGCCTGATGCTTTATCCCCA
SCD1	Stearoyl-CoA desaturase 1	TCTTCCTTATCATTGCCAACACCA	GCGTTGAGCACCAGAGTGTATCG
SOD1	Superoxide dismutase 1	AACCAGTTGTGTTGTCAGGAC	CCACCATGTTTCTTAGAGTGAGG
SREBP-1c	Sterol regulatory element-binding protein 1C	CAGGCTGAGAAAGGATGCTC	TCAGTGCCAGGTTAGAAGCA
SRXN1	Sulfiredoxin-1	AGGGGCTTCTGCAAACCTA	TGGCATAGCTACCTCACTGCT
TGFβ	Transforming growth factor beta	TCATGAGGCCAATTCCAGTAAA	ATTCCTGGCGTTACCTTGG
TIMP1	Metallopeptidase inhibitor 1	CCTGGTCATAAGGGCTAAATTCA	TTAGTCATCTTGATCTTATAACGCTGC
TNFα	Tumor necrosis factor alpha	AAAGCATGATCCGAGATGTG	AGCAGGAATGAGAAGAGGCT
UCP2	Uncouple protein 2	GCTGGTGGTGGTCGGAGATA	ACTGGCCCAAGGCAGAGTT
XBP1	X-box binding protein 1	TCCGCAGCACTCAGACTAC	CCGTGTATCTCAGCGTCTCC
β-ATCTIN	Actin beta	CTGAGAGGGAAATCGTGCGT	TGTTGGCATAGAGGTCTTTACGG

Human Gene	Abbreviations	Forward Primer	Reverse Primer
FAS	Fas cell surface death receptor	GAAACTGCAGGAGCTGTC	CACGGAGTTGAGGCGGAT
ACC1	Acetyl-CoA carboxyltransferase subunit alpha	CTGCCTGGGTTTGGGGATAA	GCACCCTCTTCACCCCTTAA
ACOX	Peroxisomal acyl-coenzyme A oxidase	GCCATCACGCTCGGCTAATT	TGAGGTGGCTTGTGGTTA
ATF6	Activating transcription factor 6	ATCACCTGCTATTACCAGCTACCAC	TGACCTGACAGTCAATCTGCATC
CTGF	Connective tissue growth factor	CAGCATGGACGTTCGTCTG	AACCACGGTTTGGTCCTTGG
CYP2E1	Cytochrome P450 family 2 subfamily E member 1	ATGTCTGCCCTCGGAGTCA	CGATGATGGGAAGCGGGAAA
EIF2α	Eukaryotic translation initiation factor 2 subunit alpha	TCACGACAGAAGTGCCCAAGA	GAAAGTCCAAGGCCTGCAAGA
GSTM1	Glutathione S-transferase mu 1	TCTGCCCTACTTGATTGATGGG	TCCACACGAATCTTCTCCTCT
IL-1α	Interleukin 1 alpha	GGCTGCATGGATCAATCTGTG	TCTTCAGAACCTTCCCGTTGG
IL-6	Interleukin 6	ACTCACCTCTTCAGAACGAATTG	CCATCTTTGGAAGGTTCAGGTTG
IL-8	Interleukin-8	GAATTCTCTTGGCTGGCTTCCTTAC	GATGTGCTTTTCGTTGGGGAAGATG
iNOS	Inducible nitric oxide synthase	AGGGACAAGCCTACCCCTC	CTCATCTCCCGTCAGTTGGT
LCAD	Long-chain acyl-CoA	TGCAATAGCAATGACAGAGCC	CGCAACTACAATCACAACATCAC
MMP12	Matrix metallopeptidase 12	GATCCAAAGGCCGTAATGTTCC	TGAATGCCACGTATGTCATCAG
MMP13	Matrix metallopeptidase 13	ACTGAGAGGCTCCGAGAAATG	GAACCCCGCATCTTGGCTT
MMP2	Matrix metallopeptidase 2	GATACCCCTTTGACGGTAAGGA	CCTTCTCCCAAGGTCCATAGC
MMP9	Matrix metallopeptidase 9	AGACCTGGGCAGATTCCAAAC	CGGCAAGTCTTCCGAGTAGT
NQO1	NAD(P)H quinone dehydrogenase 1	CCTCTATGCCATGAACTTCAATCC	GAACTGGAATATCACAAGGTCTGCG
OPN	Secreted phosphoprotein 1	AGAGTGCTGAAACCCACAGC	GGAATTCACGGCTGACTTTGG
PDK4	Pyruvate dehydrogenase kinase 4	GGAGCATTTCTCGCGCTACA	ACAGGCAATTCTTGTCGCAAA
PERK	Pancreatic eIF-2alpha kinase	CCAAGCTGTACATGAGCCCAGA	TTTCTGAGTGAACAGTGGTGGAAAC

SOD1	Superoxide dismutase 1	GGTGGGCCAAAGGATGAAGAG	CCACAAGCCAAACGACTTCC
SREBP-1c	Sterol regulatory element-binding protein 1C	CTTTGCCCACCCTGGTGAGT	GGTTCTCCTGCTTGAGTTTCTGG
SREBP-2	Sterol regulatory element binding transcription factor 2	ACAACCCATAATATCATTGAGAAACG	TTGTGCATCTTGGCGTCTGT
TFAM	Transcription factor A, mitochondrial	ATAGGCACAGGAAACCAGTTAG	GCAGAAGTCCATGAGCTGAATA
TIMP1	Metallopeptidase inhibitor 1	AGGTGGTCTCGTTGATTCGT	GTAAGGCCTGTAGCTGTGCC
TNFα	Tumour necrosis factor alpha-like	CTGCCTGCTGCACTTTGGAG	ACATGGGCTACAGGCTTGTCACT
Vimentin	Vimentin-like	AAATGGCTCGTCACCTTCGT	CAGCTTCCTGTAGGTGGCAA
XBP1	X-box binding protein 1	CAGCTCAGACTGCCAGAGATC	CAATACCGCCAGAATCCATGG

Supplementary Table 2. *Primer sequences for real-time RT-PCR.*

Antibody	Abbreviations	Company	Dilution	Catalogue number
Akt	Protein kinase B	Cell Signaling Technology	1:1000	#9272
pAkt	Phosphorylation Protein kinase B	Cell Signaling Technology	1:1000	#9271
αSMA	α-smooth muscle actin	Abcam	1:1000	Ab7817
Col1α1	Collagen type I alpha 1	Boster	1:400	BA0325
NFκB p65	Nuclear factor kappa-light-chain-enhancer of activated B	Cell Signaling Technology	1:1000	#8242
pNFκB p65	Phosphorylation nuclear factor kappa-light-chain-enhancer of activated B	Abcam	1:5000	ab86299
JNK	Phosphorylation c-Jun N-terminal kinases	Proteintech	1:2000	51151-1-AP
pJNK	c-Jun N-terminal kinases	Cell Signaling Technology	1:500	#9255
PPARα	Peroxisome proliferator activated receptor alpha	Boster	1:400	BA1691
PPARγ	Peroxisome proliferator activated receptor gamma	Cell Signaling Technology	1:1000	#2435
PI3K	Phosphatidylinositol-3-kinases	Abcam	1:1000	ab191606
pPI3K	Phosphorylation phosphatidylinositol-3-kinases	Cell Signaling Technology	1:1000	#4228
PERK	Protein kinase R-like endoplasmic reticulum kinase	Cell Signaling Technology	1:1000	#3192S
pPERK	Phosphorylation protein kinase R-like endoplasmic reticulum kinase	Cell Signaling Technology	1:1000	#3179

PGC1-α	Peroxisome proliferator-activated receptor gamma coactivator-1 alpha	Abcam	1:1000	ab54481
CYP2E1	Cytochrome P450 family 2 subfamily E member 1	Abcam	1:5000	ab28146
eIF2α	Eukaryotic translation initiation factor 2 subunit alpha	Cell Signaling Technology	1:1000	#9722
p-eIF2α	Phosphorylation Eukaryotic translation initiation factor 2 subunit alpha	Cell Signaling Technology	1:1000	#9721
AMPKα	protein kinase AMP-activated catalytic subunit alpha 1	Cell Signaling Technology	1:1000	#2532
pAMPKα	Phosphorylation protein kinase AMP-activated catalytic subunit alpha 1	Cell Signaling Technology	1:1000	#2535
GAPDH	Glyceraldehyde-3-phosphate	Cell Signaling Technology	1:1000	#2118
His-Tb	Tb-anti-Histone	ThemoFisher Scientific	1:200	PV5863
Goat anti-Rabbit IgG	Goat anti-Rabbit IgG	Zsbio	1:5000	ZDR-5118
Goat anti-Mouse IgG	Goat anti-Mouse IgG	Zsbio	1:5000	ZDR-5307

Supplementary Table 3. *Antibodies used for western blotting.*

Abbres	Full Name
Fmoc	Fluorenylmethyloxycarbonyl protecting group
HPLC	High-performance liquid chromatography
CCK8	Cell Counting Kit-8
OGTT	Oral glucose tolerance test
ITT	Insulin tolerance test
ALT	Aanine aminotransferase
AST	Asparate aminotransferase
TC	Total cholesterol
TG	Triglycerides
HDL	High-density lipoprotein cholesterol
LDL	Low-density lipoprotein cholesterol
FFA	Nonesterified Free fatty acids
HYP	Hydroxyproline
HSCs	Hepatic stellate cells
ECM	Extracellular matrix
CCl_4	Carbon tetrachloride
CD68	Cluster of differentiation 68
TUNEL	Terminal deoxynucleotidyl transferase-mediated dUTP-biotin nick end labeling

DAPI	4,6-diamidino-2-phenylindole
HRP	horseradish peroxidase
DAB	3,3'-diaminobenzidine
PA	Palmitic acid
BSA	Bovine serum albumin

Supplementary Table 4. *Abbreviations.*
