# Supplementary material for: AdipoR1/AdipoR2 dual agonist recovers nonalcoholic steatohepatitis and related fibrosis via endoplasmic reticulum-mitochondria axis
Source: Nat Commun. 2020 Nov 16;11:5807. doi: 10.1038/s41467-020-19668-y (PMC7669869; doi:10.1038/s41467-020-19668-y)
Supplement: Supplementary file 3 — Reporting Summary [file 41467_2020_19668_MOESM3_ESM.pdf]

nature research

Corresponding author(s): Xianxing Jiang; Rui Wang; Huihao Zhou

Last updated by author(s): Sep.5,2020

Reporting Summary

Nature Research wishes to improve the reproducibility of the work that we publish. This form provides structure for consistency and transparency in reporting. For further information on Nature Research policies, see our Editorial Policies and the Editorial Policy Checklist.

Statistics

For all statistical analyses, confirm that the following items are present in the figure legend, table legend, main text, or Methods section.

n/a	Confirmed	
☐	☒	The exact sample size (n) for each experimental group/condition, given as a discrete number and unit of measurement
☐	☒	A statement on whether measurements were taken from distinct samples or whether the same sample was measured repeatedly
☐	☒	The statistical test(s) used AND whether they are one- or two-sided<br>*Only common tests should be described solely by name; describe more complex techniques in the Methods section.*
☐	☒	A description of all covariates tested
☐	☒	A description of any assumptions or corrections, such as tests of normality and adjustment for multiple comparisons
☐	☒	A full description of the statistical parameters including central tendency (e.g. means) or other basic estimates (e.g. regression coefficient) AND variation (e.g. standard deviation) or associated estimates of uncertainty (e.g. confidence intervals)
☐	☒	For null hypothesis testing, the test statistic (e.g. F, t, r) with confidence intervals, effect sizes, degrees of freedom and P value noted<br>*Give P values as exact values whenever suitable.*
☒	☐	For Bayesian analysis, information on the choice of priors and Markov chain Monte Carlo settings
☐	☒	For hierarchical and complex designs, identification of the appropriate level for tests and full reporting of outcomes
☒	☐	Estimates of effect sizes (e.g. Cohen's d, Pearson's r), indicating how they were calculated

Our web collection on statistics for biologists contains articles on many of the points above.

Software and code

Policy information about availability of computer code

Data collection	Data were collected with various laboratory instruments and equipments. such as: MBHA amide resin (GL Biochem, Shanghai, China), Reverse phase high performance liquid chromatography mass spectrometric (RP-HPLC-MS, Agilent Technologies, USA), Flex Station 3 plate reader (Molecular Devices, USA), Laser confocal microscope (Olympus FV3000, Japan), Molecular Operating Environment (MOE) version 2013.8, Discovery Studio (DS) version 3.5, OneTouch UltraVue Blood Glucose Meter and test strips (Johnson&Johnson, USA), Nanodrop 2000 (ThermoFisher Scientific, USA), Tanon-Image Software (Shanghai, China), Confocal microscopy (Olympus, Japan), LC-MS/MS (ThermoFisher Scientific, USA), Phoenix WinNonlin (version 8.0, Pharsight, USA), Light Cycler 480 Real-Time PCR System (Roche, Swiss)
Data analysis	The positive area of all stained liver slices was quantified by ImageJ (https://imagej.nih.gov/ij/)(V1.8.0.112). All data in this study were shown as the mean ± SEM (Standard Error). Student's two-tailed t test was used to compare the mean of two groups of samples. All statistical analyses were performed with GraphPad Prism software (https://www.graphpad.com/)(V7.0).

For manuscripts utilizing custom algorithms or software that are central to the research but not yet described in published literature, software must be made available to editors and reviewers. We strongly encourage code deposition in a community repository (e.g. GitHub). See the Nature Research guidelines for submitting code & software for further information.

Data

Policy information about availability of data

All manuscripts must include a data availability statement. This statement should provide the following information, where applicable:

- Accession codes, unique identifiers, or web links for publicly available datasets
- A list of figures that have associated raw data
- A description of any restrictions on data availability

Source data are provided with this paper. Source data including the information about the plasmid construction of adiponectin receptor 1/2, the related sequencing results of animal samples, and the other involved data in the manuscript, DOI: 10.6084/m9.figshare.12922505.

Field-specific reporting

Please select the one below that is the best fit for your research. If you are not sure, read the appropriate sections before making your selection.

- [x] Life sciences
- [ ] Behavioural & social sciences
- [ ] Ecological, evolutionary & environmental sciences

For a reference copy of the document with all sections, see nature.com/documents/nr-reporting-summary-flat.pdf

Life sciences study design

All studies must disclose on these points even when the disclosure is negative.

Sample size	No statistical methods were used to predetermine sample size. Experiments were performed three times independently unless indicated. In previous studies using related experiments, the sample size has been determined to be sufficient to ensure reproducibility.
Data exclusions	According to the samples, no data was excluded.
Replication	For each experiment, triplicates were performed. All attempts of replication are successful.
Randomization	All the samples/organisms were divided randomly into experimental groups in these experiments
Blinding	For all the experiments , the investigators were blinded to group allocation during data collection and/or analysis.

Reporting for specific materials, systems and methods

We require information from authors about some types of materials, experimental systems and methods used in many studies. Here, indicate whether each material, system or method listed is relevant to your study. If you are not sure if a list item applies to your research, read the appropriate section before selecting a response.

Materials & experimental systems

n/a	Involved in the study	
☐	☒	Antibodies
☐	☒	Eukaryotic cell lines
☒	☐	Palaeontology and archaeology
☐	☒	Animals and other organisms
☒	☐	Human research participants
☒	☐	Clinical data
☒	☐	Dual use research of concern

Methods

n/a	Involved in the study	
☒	☐	ChIP-seq
☒	☐	Flow cytometry
☒	☐	MRI-based neuroimaging

Antibodies

Antibodies used	Akt (Cell Signaling Technology; 1:1000; #9272), pAkt (Cell Signaling Technology ; 1:1000; #9271),αSMA (Abcam;1:1000; ab7817), Col1α1 (Boster;1:400; BA0325), NFκB p65 (Cell Signaling Technology; 1:1000; #8242), pNFκB p65 (Abcam;1:5000; ab86299), JNK (Proteintech; 1:2000; 51151-1-AP), pJNK (Cell Signaling Technology ; 1:500; #9255), PPARα (Boster; 1:400;BA1691), PPARγ (Cell Signaling Technology; 1:1000; #2435), PI3K (Abcam; 1:1000; ab191606), pPI3K (Cell Signaling Technology; 1:1000; #4228), PERK (Cell Signaling Technology ; 1:1000; #3192S), pPERK (Cell Signaling Technology ; 1:1000; #3179), PGC1-α (Abcam;1:1000;ab54481), CYP2E1 (Abcam;1:5000; ab28146), eIF2α (Cell Signaling Technology ;1:1000;#9722), p-eIF2α (Cell Signaling Technology ; 1:1000; #9721), AMPKα (Cell Signaling Technology; 1:1000; #2532), pAMPKα (Cell Signaling Technology ; 1:1000; #2535), GAPDH (Cell Signaling Technology; 1:1000; #2118), His-Tb (ThermoFisher Scientific; 1:200; PV5863), Goat anti-Rabbit lgG (Zsbio; 1:5000; ZDR-5118); Goat anti-Mouse lgG (Zsbio; 1:5000; ZDR-5307)
Validation	Akt (source: Rabbit; reactivity:H M R Hm Mk C Dm B Dg Pg GP; Filter:WB IP IF F), pAkt (source:Rabbit;reactivity: H M R Hm Dm B Dg Pg; Filter:WB IP IF F), αSMA (source:Mouse; reactivity:M Rat R H Pig; Filter: ICC IF IHC-P WB F), Col1α1(source:Rabbit; reactivity:H M

Rat; Filter:WB IHC-P IHC-F ICC IF), NFκB p65 (source:Rabbit; reactivity:H M R Hm Mk Dg; Filter:WB IP IHC IF F ChIP C&R), pNFκB p65 (source:Rabbit; reactivity:H M Rat; Filter:IHC-P WB), JNK (source:Rabbit; reactivity:H M; Filter:WB IP IF), pJNK(source:Mouse; reactivity:H M R Hm Sc; Filter:WB IP IF F), PPARα (source:Rabbit; reactivity:H M Rat; Filter:WB IHC-P IHC-F ICC), PPARγ (source:Rabbit; reactivity:H M; Filter:WB IHC IF ChIP), PI3K (source:Rabbit; reactivity:M Rat H; Filter:WB ICC IF F IP), pPI3K(source:Rabbit; reactivity:M; Filter:WB IP), PERK (source:Rabbit; reactivity:H M R Mk; Filter:WB), PGC1α(source:Rabbit; reactivity:H M; Filter:WB), CYP2E1 (source:Rabbit; reactivity:M Rat R Pg Dg H Mk; Filter:WB ICC IF), eIF2α (source:Rabbit; reactivity:H M R Mk; Filter:WB),p-eIF2α (source:Rabbit; reactivity:H M R Mk Dm; Filter:WB), AMPKα(source:Rabbit; reactivity:H M R Hm Mk; Filter:WB IP),pAMPKα (source:Rabbit; reactivity: H M R Hm Mk Dm Sc; Filter:WB IP IHC), GAPDH (source:Rabbit; reactivity:H M R Mk B Pg; Filter:WB IHC IF F),His-Tb (application:Biochemical Kinase Assays; form:Primary; Label Type:Lanthanide Chelates; Label or Dye:Tb), Goat anti-Rabbit lgG (source: Goat; Conjugate: HRP), Goat anti-Mouse lgG (source: Goat;Conjugate: HRP)

Eukaryotic cell lines

Policy information about cell lines

Cell line source(s)	HepG2, LX2, HEK293 cells line were purchased from Type Culture Collection of the Chinese Academy of Sciences (Shanghai, China).
Authentication	Cells were identified by frequent morphology check under microscopy.
Mycoplasma contamination	All cell lines tested negative for mycoplasma contamination.
Commonly misidentified lines (See ICLAC register)	No commonly misidentified cell lines were used in the study.

Animals and other organisms

Policy information about studies involving animals; ARRIVE guidelines recommended for reporting animal research

Laboratory animals	C57BL/6J mice (male, 6-8 weeks); SpragueDawley (SD) (male, 6-8 weeks)
Wild animals	No.
Field-collected samples	The study did not involve samples collected from the field.
Ethics oversight	All animal procedures were approved by Animal Care and Use Committee of the Sun Yat-sen University. The experimental mice received humane care according to the criteria outlined in the Guide for the Care and Use of Laboratory Animals.

Note that full information on the approval of the study protocol must also be provided in the manuscript.
