# Supplementary material for: AdipoR1/AdipoR2 dual agonist recovers nonalcoholic steatohepatitis and related fibrosis via endoplasmic reticulum-mitochondria axis
Source: Nat Commun. 2020 Nov 16;11:5807. doi: 10.1038/s41467-020-19668-y (PMC7669869; doi:10.1038/s41467-020-19668-y)
Supplement: Supplementary file 2 — Peer Review File [file 41467_2020_19668_MOESM2_ESM.pdf]

Reviewers' Comments:

Reviewer #1:
Remarks to the Author:
This manuscript by Xu et al deals with the topic of activation of adipoR1 and R2 to improve the pathophysiology of NASH with the hope of improving fibrosis. The study is well performed, and the results are convincing. My issue is in relation to the originality of the work. It has been well established that NASH is associated with low levels of serum adiponectin and that in animal models blocking the adiponectin axis results in worse steatohepatitis and liver fibrosis. As such I believe that there is little new information on the biology of NASH. There is of-course new and convincing information on the agonist JT003 which improved histological features of NASH and even CCL4 induced liver fibrosis. It is an editorial decision if this is sufficient for publication. Also, I did not see any acknowledgements. Was this work supported by a company, and on a related issue do any of the authors have any conflicts such as intellectual property or other commercial interests? This is not an issue but should just be stated. Finally suggest that the writing be reviewed as it can be improved.

For example:
It should be "anti-LOXL2 antibodies" instead of "LOXL2 antibodies".

Incorrect use of the word "affirm" many times.

Claiming that adiponectin has a "curative effect". It is of-course a potential therapy not a cure.

Also claimed that mitochondrial biogenesis was improved, but no real biogenesis data, only mitochondrial gene expression data.

Reviewer #2:
Remarks to the Author:
In the manuscript by Xu, Jiang, et al., the authors describe the effects of a newly developed peptide derived from adiponectin, which is an analog of the previously studied ADP355 peptide but with some improved pharmaceutical properties and suggested dual activity at both adiponectin receptors, to reduce the damaging effects of high-fat diet or chemical injury (CCl_4) to liver health in mice. As this is a pharmacology-focused manuscript, the experiments are lacking important controls and dose/concentration responses. Furthermore, the authors fail to definitively show that this new analog (JT003) is indeed a dual agonist through specific binding or potency assays selective for the two receptor isoforms. Oligonucleotide-mediated knockdown of both receptor isoforms appears to blunt hepatocyte cellular binding of a labeled analog, and also altered transcriptional signatures in vitro. However, these experiments were not conducted with the individual knockdown of the receptor nor controls were not included. Thus, it is an incorrect statement to call this a dual agonist due to these deficiencies. Without that dual agonism, other than an improved proteolytic stability by use of norvaline substitution, the new analog JT003 fails to distinguish itself from ADP355. Furthermore, a head-to-head comparison of JT003 to ADP355 at a few dose levels should have been completed, because ADP355 has previously been shown to improve fibrosis in CCl_4-treated mice (Kumar et al. PLOS One 2014) and other models of liver injury (TAA; Wang et al. Scientific Reports 2016) and fibrosis (Marangoni et al. Scientific Reports 2017). Furthermore, the small molecule adiponectin receptor agonist (AdipoRon; Okada-Iwabu et al. Nature 2013) has also been shown to improve liver injury in mice (Wang et al. Eur J Pharm Sci 2016). By all accounts, this is the first test of these class of compounds in high-fat fed mice, which provides some novelty, but effects on body weight need to be reported differently and it is inaccurate to call this a "NASH model." This is a model of fatty liver disease with signs of inflammation but typically without a fibrotic liver. Thus, it is more accurate to call this a model of NAFLD, not NASH. High fat diets combined with amino acid deficient diets (i.e. choline) have

proven to be another model that manifests the human NASH condition of obesity and liver fibrosis thus should be considered here for study. Even many of the cellular mechanisms studied within (AMPK, PPARa, PPARy, etc.) have already been attributed to the mechanism of action for activating the adiponectin receptors. Perhaps there is a degree of novelty in the proposed mechanism of inflammasome modulation and relation to ER stress, but this is just an aspect of the manuscript. Therefore, to move the scientific community forward, a better understanding of how this molecule differs from predecessor molecules is necessary. However, without standard pharmacological measures, comparisons, and controls in the current data set, combined with a restricted novelty and deficiencies in the presentation of the material (figures and written text), it is recommended to not publish the article in its current state.

Major and Minor Specific Comments

Head-to-head in vivo profiling of JT003 versus ADP355 (at multiple dose levels) is vital for understanding if JT003 is indeed a superior molecule and should be done in the HFD and CCl4 model herein, but also considered in amino acid deprived mouse models of NASH.

For the in vitro data confirming activity of the analogs, a rank order was generated based on single concentration experiments like in Figure 1B. As this is a pharmacological study, a concentration dependent association is important. Can the authors include concentrations higher and lower than 200 uM? Also, a comparison to native adiponectin is warranted.

In Figure 1D, the authors show that fluorescently labeled JT003 binds to HepG2 and LX2 cells, and then fluorescent signal is attenuated with knockdown of AdipoR1 and R2. Can the authors provide activity data for the fluorescently labeled JT003 analog? Can the authors provide data showing FITC-labeling of a molecule with weak affinity for R1/R2 shows less signal? This could be JT006, which showed the least aSMA signal (Figure 1C) and little prevention of palmitic acid induced lipid droplets (Figure 1B). The sequence scrambled peptide from Ma. et al. 2017 (peptide 56) could also be used as a control. Can the authors test for biological effects or cellular staining in cells that inherently do not express the receptors? These are important controls as there is a lack of pure binding/affinity data for the peptides to the two receptor isoforms, nor are there robust potency assays utilized.

Can the authors plot the body weight change from baseline/start of treatment (in grams or percentage) during the 4 weeks treatment of DIO mice shown in Figure 2A? Body weight was measured 3 times per week according to the methods. The statistical significance indicators need to be defined in the figure legend. Without the longitudinal body weight data, it cannot be determined if there is significant difference in the body weight following treatment, which the authors claim to be different from vehicle treated HFD controls. Please indicate the dose of JT003 (500 ug/kg) used either in the figure legend or depicted on the graphs themselves. How was this dose selected? Again, as this is a pharmacology study, a dose titration in the HFD mice should be included. Furthermore, it is not correct to call this a NASH model. This is a model of fatty liver or NAFLD since fibrosis is not typically evident in these animals. Sirius Red staining should be assessed in the livers to determine amount of fibrosis.

The ALT data in Figure 2A should be a separate, independent panel within the figure. Similar for the adiponectin and insulin measures depicted on the right in Figure 2B.

The authors state in the results "the increase in circulating adiponectin in the HFD group was reverted remarkably after JT003 treating." However, the data in Figure 2B shows that adiponectin levels are increased following JT003 treatment relative to vehicle HFD controls. Either there is a typo in the written results or there is a misunderstanding of the data. Please comment.

It is unclear what concentration of JT003 was used in Figure 3 as it is not in the legend nor depicted on the graphs. It is assumed it was 200 uM like in Figure 1. Please clarify.

The individual graphs in Figure 3D should be separated into individual panels.

The authors claim that phosphorylated AMPK is increased following JT003 treatment in Figure 3F. However, the western blots do not support this claim nor does that ratio. Furthermore, the western blots for PPARa also do not support the claims, or at least suggest that a single lane/mouse is skewing the results to suggest PPARa protein is increased. Please comment. Overall, the western blots, also in Figure 4, are variable and thus an accurate assessment of the quantification requires an examination of other blots. Particularly worrisome are the western blots for phosphorylated targets where the protein itself seems to by modulated by diet and or

treatment.
In the CCl4 mouse model (Figure 5), can the authors report body weight curves throughout treatment. Can the authors have the ALT measures as a separate panel within the figure? Again, the effects of different doses of JT003 should be studied.
The authors choose words such as "ameliorated" and "alleviation" which suggest that the pathological effects were completely reversed with JT003 treatment. However, that is not the case so the authors should use words such as "reduced" or "improved." Furthermore, the statement of curative effects in the discussion should be tempered as the disease was not cured in these mice, nor is this the first report of adiponectin peptides demonstrating effects on liver metabolism.
Overall, the figures are hardly legible. Please increase the size of the symbols and decrease the size of the error bars for the graphed data.
The text requires significant proof-reading and revision as there are numerous grammatical mistakes.

Reviewer #3:
Remarks to the Author:
Xu et al. evaluated positive effects of a peptide JT003 on NASH/fibrosis model. Overall effects of it coincide with adiponectin function on NASH model studied by using adiponectin KO mice. AMPK and PPARα signaling may also well coincide with globular adiponectin-AdipoRs signaling. However, there is no experiment indicating the binding of JT003 to AdipoRs, nor competitive binding of JT003 to adiponectin, ideally native one in serum/plasma, or at least globular form of adiponectin, which was initially shown to bind AdipoRs. Such experimental evidences to show that this peptide truly function through binding to AdipoRs are necessary.

Response to the Comments of Reviewers

Reviewer: 1

This manuscript by Xu et al deals with the topic of activation of adipoR1 and R2 to improve the pathophysiology of NASH with the hope of improving fibrosis. The study is well performed, and the results are convincing. My issue is in relation to the originality of the work. It has been well established that NASH is associated with low levels of serum adiponectin and that in animal models blocking the adiponectin axis results in worse steatohepatitis and liver fibrosis. As such I believe that there is new information on the biology of NASH. There is of-course new and convincing information on the agonist JT003 which improved histological features of NASH and even CCL4 induced liver fibrosis. It is an editorial decision if this is sufficient for publication. Also, I did not see any acknowledgements. Was this work supported by a company, and on a related issue do any of the authors have any conflicts such as intellectual property or other commercial interests? This is not an issue but should just be stated. Finally suggest that the writing be reviewed as it can be improved.

Answer: Thank you very much. We designed and optimized the dual agonist of AdipoR1 and AdipoR2 based on the natural ligand of these two receptors. We believed that JT003 was first reported as the peptide agonist for both AdipoR1 and AdipoR2.

The 'conflicts of interest', 'author contributions' and 'acknowledgements' sections are located at page 14 and 15 of the manuscript.

Many thanks to your reminding. We also have our writing revised.

For example:

It should be "anti-LOXL2 antibodies" instead of "LOXL2 antibodies".

Answer: Thank you very much. In page 3, paragraph 3, line 9, the "LOXL2 antibodies" has been revised with "anti-LOXL2 antibodies".

Incorrect use of the word "affirm" many times.

Answer: Thank you for your comments. Page 4, paragraph 2, line9, the word "affirmed" was revised with "confirmed". Line 13, "affirmed" was replaced by "demonstrated".

Claiming that adiponectin has a “curative effect”. It is of-course a potential therapy not a cure.

Answer: Thank you for your suggestion. In page 12, paragraph 1, line 9 of the manuscript, the word “curative” has been changed into “potential therapeutical”.

Also claimed that mitochondrial biogenesis was improved, but no real biogenesis data, only mitochondrial gene expression data.

Answer: Thank you very much. Firstly, Previous studies had implied that AMPK directly mediates the mitochondrial biogenesis (see reference 10). Besides, we performed the mitochondrial content assay which is also one aspect to demonstrate the mitochondrial biogenesis (Fig. 4b). Thirdly, the mitochondrial biogenesis regulators PGC1α and PGC1β expression were both accelerated with the dual agonist treatment (Fig. 4f, g). In summary, we could draw the conclusion that the mitochondrial biogenesis was improved.

Reviewer: 2

In the manuscript by Xu, Jiang, et al., the authors describe the effects of a newly developed peptide derived from adiponectin, which is an analog of the previously studied ADP355 peptide but with some improved pharmaceutical properties and suggested dual activity at both adiponectin receptors, to reduce the damaging effects of high-fat diet or chemical injury (CCl_4) to liver health in mice. As this is a pharmacology-focused manuscript, the experiments are lacking important controls and dose/concentration responses. Furthermore, the authors fail to definitively show that this new analog (JT003) is indeed a dual agonist through specific binding or potency assays selective for the two receptor isoforms. Oligonucleotide-mediated knockdown of both receptor isoforms appears to blunt hepatocyte cellular binding of a labeled analog, and also altered transcriptional signatures in vitro. However, these experiments were not conducted with the individual knockdown of the receptor nor controls were not included. Thus, it is an incorrect statement to call this a dual agonist due to these deficiencies. Without that dual agonism, other than an improved proteolytic stability by use of norvaline substitution, the new analog JT003 fails to distinguish itself from ADP355. Furthermore, a head-to-head comparison of JT003 to ADP355 at a few dose levels should have been completed, because ADP355

has previously been shown to improve fibrosis in CCl4-treated mice (Kumar et al. PLOS One 2014) and other models of liver injury (TAA; Wang et al. Scientific Reports 2016) and fibrosis (Marangoni et al. Scientific Reports 2017). Furthermore, the small molecule adiponectin receptor agonist (AdipoRon; Okada-Iwabu et al. Nature 2013) has also been shown to improve liver injury in mice (Wang et al. Eur J Pharm Sci 2016). By all accounts, this is the first test of these class of compounds in high-fat fed mice, which provides some novelty, but effects on body weight need to be reported differently and it is inaccurate to call this a "NASH model." This is a model of fatty liver disease with signs of inflammation but typically without a fibrotic liver. Thus, it is more accurate to call this a model of NAFLD, not NASH. High fat diets combined with amino acid deficient diets (i.e. choline) have proven to be another model that manifests the human NASH condition of obesity and liver fibrosis thus should be considered here for study. Even many of the cellular mechanisms studied within (AMPK, PPARa, PPARγ, etc.) have already been attributed to the mechanism of action for activating the adiponectin receptors. Perhaps there is a degree of novelty in the proposed mechanism of inflammasome modulation and relation to ER stress, but this is just an aspect of the manuscript. Therefore, to move the scientific community forward, a better understanding of how this molecule differs from predecessor molecules is necessary. However, without standard pharmacological measures, comparisons, and controls in the current data set, combined with a restricted novelty and deficiencies in the presentation of the material (figures and written text), it is recommended to not publish the article in its current state.

Answer: Thank you for your detailed comments.

We do think about the controls and dose/concentration responses, however, we only choose the optimum concentration of JT003 to further conduct the mechanism studies. Thanks to your valuable suggestions, we ran a series of follow-up experiments which had ADP355 peptide as the controls and different concentrations of both ADP355 and JT003 are performed to illustrate the dose/concentration responses.

We proposed that JT003 is the dual agonist of both AdipoR1 and AdipoR2. Page 6, paragraph 2, line 5 and Figure 1 d, the individual knockdown of the two receptors experiments were performed to support the proposal. Our results indicated indeed that JT003 could bind to the two receptor isoforms specifically.

Page 5, paragraph 3, line 5 and line 6 as well as supplementary Figure 3 and 4, a

head-to-head comparison of JT003 to ADP355 at a few dose levels have been completed *in vitro*. The sentence "Lipid droplets accumulation caused by PA could be reduced by the native $gAd_{155\text{-}161}$ peptide and the analogues we designed[29], of which JT003 exhibited the best potential (Fig. 1b)." was revised with "Lipid droplets accumulation caused by PA could be reduced by the native $gAd_{155\text{-}161}$ peptide and the analogues we designed as well as previously reported peptide ADP355[28, 29], of which JT003 exhibited the best potential (Fig.1b, Supplementary Fig. 3)." Page 6, paragraph 1, line 1, "Supplementary Fig. 4" was added.

Page 6, paragraph 3, line 6 and supplementary Figure 7, the body weight has been reported. "Treatment with JT003 significantly smoothed the liver and decreased the liver sizes (Supplementary Fig. 5)." was replaced with "Treatment with JT003 significantly reduced the weight gain, smoothed the liver and decreased the liver sizes (Fig. 2a, Supplementary Fig. 7)."

Nonalcoholic fatty liver disease occurs in the context of over-nutrition, particularly when associated with central obesity, adipose dysfunction and inflammation and insulin resistance. Its pathological spectrum encompasses simple steatosis, nonalcoholic steatohepatitis (NASH) and pericellular fibrosis that can slowly lead to cirrhosis. High fat diet induced fatty liver disease animal models are widely used in the drug development researches. Thus, high fat diet induced NASH mouse model was used in the current study. However, according to the reviewer's suggestion, we also conducted high fat diet combine with methionine-choline deficient (MCD) diet to manifest the human NASH condition of obesity and liver fibrosis, further histological studies were performed as well. Besides, we also performed the comparison experiment to declare JT003 differs from predecessor molecules. Page 6, paragraph 3, line 7, the description "The ALT level were also reduced in HFD fed mice after JT003 treatment (Fig. 2b). Besides, in HFD combine with methionine and choline deficient (MCD) diet NASH mouse model, compare to the previous reported peptide ADP355, JT003 exhibited excellent ability of reducing body weight and inhibiting lipid accumulation." was added.

Major and Minor Specific Comments

Head-to-head in vivo profiling of JT003 versus ADP355 (at multiple dose levels) is vital for understanding if JT003 is indeed a superior molecule and should be done in the HFD and CCl4 model herein, but also considered in amino acid deprived mouse

models of NASH.

Answer: Thank you very much. We performed the head-to-head in vivo analysis to compare JT003 with ADP355 at multiple dose levels both *in vitro* and *in vivo*, in HFD and CCl_4 models, as well as HFD combine with MCD diets. Supplementary figure 3, 4, 8, 9, 10 and 12 were revised.

For the in vitro data confirming activity of the analogs, a rank order was generated based on single concentration experiments like in Figure 1B. As this is a pharmacological study, a concentration dependent association is important. Can the authors include concentrations higher and lower than 200 uM? Also, a comparison to native adiponectin is warranted.

Answer: Thank you very much. In Supplementary figure 3 and 4, various doses were conduct to illustrate the concentration dependent association. Instead of native adiponectin, a comparison to ADP355 has also been performed.

In Figure 1D, the authors show that fluorescently labeled JT003 binds to HepG2 and LX2 cells, and then fluorescent signal is attenuated with knockdown of AdipoR1 and R2. Can the authors provide activity data for the fluorescently labeled JT003 analog? Can the authors provide data showing FITC-labeling of a molecule with weak affinity for R1/R2 shows less signal? This could be JT006, which showed the least aSMA signal (Figure 1C) and little prevention of palmitic acid induced lipid droplets (Figure 1B). The sequence scrambled peptide from Ma. et al. 2017 (peptide 56) could also be used as a control. Can the authors test for biological effects or cellular staining in cells that inherently do not express the receptors? These are important controls as there is a lack of pure binding/affinity data for the peptides to the two receptor isoforms, nor are there robust potency assays utilized.

Answer: Thank you for your valuable advice very much. The previous screening studied have showed us already JT003 was the most potential peptide which could ameliorate NASH and related fibrosis. We thought maybe it is not necessary to synthesise all the fluorescent labeled analogues. However, we did provide the activity data for the fluorescently labeled JT006, which showed the least αSMA signal and little prevention of palmitic acid induced lipid droplets, in supplementary figure 5.

Can the authors plot the body weight change from baseline/start of treatment (in

grams or percentage) during the 4 weeks treatment of DIO mice shown in Figure 2A? Body weight was measured 3 times per week according to the methods. The statistical significance indicators need to be defined in the figure legend. Without the longitudinal body weight data, it cannot be determined if there is significant difference in the body weight following treatment, which the authors claim to be different from vehicle treated HFD controls. Please indicate the dose of JT003 (500 ug/kg) used either in the figure legend or depicted on the graphs themselves. How was this dose selected? Again, as this is a pharmacology study, a dose titration in the HFD mice should be included. Furthermore, it is not correct to call this a NASH model. This is a model of fatty liver or NAFLD since fibrosis is not typically evident in these animals. Sirius Red staining should be assessed in the livers to determine amount of fibrosis.

Answer: Thank you very much for your suggestions. The body weight change from the start of the treatment during the 4 weeks treatment of DIO mice has been showed in Figure2a. And the body weight change from baseline was presented in supplementary figure 8. The dose of JT003 was described in the figure legend. We chose this dose due to some preliminary experiment which we didn't mention in the article. Anyway, various doses were conducted in the supplementary figure 9 and 10 of this revised study. Sirius Red staining has been assessed in the livers to determine amount of fibrosis, which are presented in CCl_4 induced fibrosis mouse model.

The ALT data in Figure 2A should be a separate, independent panel within the figure. Similar for the adiponectin and insulin measures depicted on the right in Figure 2B.

Answer: Thank you very much for your detailed advices. The ALT data and adiponectin and insulin measures have been separated within the figure2.

The authors state in the results "the increase in circulating adiponectin in the HFD group was reverted remarkably after JT003 treating." However, the data in Figure 2B shows that adiponectin levels are increased following JT003 treatment relative to vehicle HFD controls. Either there is a typo in the written results or there is a misunderstanding of the data. Please comment.

Answer: Thank you very much. Really sorry about that typo in the written results. In page 6, paragraph 2, line 9, the word "increase" has been revised as "decrease".

It is unclear what concentration of JT003 was used in Figure 3 as it is not in the legend nor depicted on the graphs. It is assumed it was 200 uM like in Figure 1. Please clarify.

Answer: Thank you very much. The concentration of JT003 used in Figure 3 is 200 μM as it is in Figure 1. It has been added in the figure legend. Please see page 29, line 14, the sentence "For in vitro experiment, the concentration of JT003 is 200 μM. For the in vivo experiment, the dose of JT003 is 500 μg/kg." was added.

The individual graphs in Figure 3D should be separated into individual panels.

Answer: Thank you very much. The figures in figure 3D have been separated into individual panels.

The authors claim that phosphorylated AMPK is increased following JT003 treatment in Figure 3F. However, the western blots do not support this claim nor does that ratio. Furthermore, the western blots for PPARa also do not support the claims, or at least suggest that a single lane/mouse is skewing the results to suggest PPARa protein is increased. Please comment. Overall, the western blots, also in Figure 4, are variable and thus an accurate assessment of the quantification requires an examination of other blots. Particularly worrisome are the western blots for phosphorylated targets where the protein itself seems to by modulated by diet and or treatment.

Answer: Thank you very much. At first we do not avoid the variation during the experiment. However, we had 6 mice for each group. In the revised manuscript, we examined them all and give out the most appropriate results. Page 28, figure 3f was revised.

In the CCl4 mouse model (Figure 5), can the authors report body weight curves throughout treatment. Can the authors have the ALT measures as a separate panel within the figure? Again, the effects of different doses of JT003 should be studied.

Answer: Thank you very much. The body weight curves are reported in supplementary figure 12. The ALT measures are presented as a separate panel in figure. Also, the effects of different doses of JT003 are studied. Please see supplementary figure 12.

The authors choose words such as "ameliorated" and "alleviation" which suggest

that the pathological effects were completely reversed with JT003 treatment. However, that is not the case so the authors should use words such as “reduced” or “improved.” Furthermore, the statement of curative effects in the discussion should be tempered as the disease was not cured in these mice, nor is this the first report of adiponectin peptides demonstrating effects on liver metabolism.

Answer: Thank you very much. Page 2, paragraph 1, line 5, and page 4, paragraph1, line 14, “ameliorated” were revised with “improved”. Page 9, paragraph 3, line 5, “ameliorated” was replaced by “reduced”. Page 10, paragraph 2, line 7, “ameliorated” were altered with “improved”. Page 11, paragraph 1, line 4, “ameliorated” were revised with “decreased”. Page 11, paragraph 1, line 13, “ameliorated” were replaced with “lessened”. Page 14, paragraph 1, line 4, “ameliorated” was swapped with “reduced”. Page 9, paragraph 3, line 8, “alleviation” was changed with “improvement”. Page 14, paragraph 1, line 5, “alleviation” was revised by “decreasing”.

Overall, the figures are hardly legible. Please increase the size of the symbols and decrease the size of the error bars for the graphed data.

Answer: Thank you very much. The size of the symbols and the error bars are all increased. Please see the figures.

The text requires significant proof-reading and revision as there are numerous grammatical mistakes.

Answer: Thank you very much. According to your comments, we carefully proof-read our manuscript and had these mistakes revised.

Reviewer: 3

Xu et al. evaluated positive effects of a peptide JT003 on NASH/fibrosis model. Overall effects of it coincide with adiponectin function on NASH model studied by using adiponectin KO mice. AMPK and PPARα signaling may also well coincide with globular adiponectin-AdipoRs signaling. However, there is no experiment indicating the binding of JT003 to AdipoRs, nor competitive binding of JT003 to adiponectin, ideally native one in serum/plasma, or at least globular form of adiponectin, which was initially shown to bind AdipoRs. Such experimental evidences to show that this

peptide truly function through binding to AdipoRs are necessary.

Answer: Thank you very much for your valuable comments. The competitive binding of JT003 to globular form of adiponectin are performed. Besides, we conducted the HTRF experiment to calculate the EC_{50} of JT003. Please see Figure 8.

It would be highly appreciated if you are so kind to give a favorable consideration on it.

We are looking forward to your reply.

Sincerely,

Xianxing Jiang Ph.D
Professor
Medicinal Chemistry and Biochemistry
School of Pharmaceutical Sciences
Sun Yat-sen University
Waihuan East Road 132, Guangzhou University City
Guangzhou 510006, China
Mobile: +86 18819810352
E-mail: jiangxx5@mail.sysu.edu.cn

Response to the Comments of Reviewers in Detail

Reviewer: 1

This manuscript by Xu et al deals with the topic of activation of adipoR1 and R2 to improve the pathophysiology of NASH with the hope of improving fibrosis. The study is well performed, and the results are convincing. My issue is in relation to the originality of the work. It has been well established that NASH is associated with low levels of serum adiponectin and that in animal models blocking the adiponectin axis results in worse steatohepatitis and liver fibrosis. As such I believe that there is new information on the biology of NASH. There is of-course new and convincing information on the agonist JT003 which improved histological features of NASH and even CCL4 induced liver fibrosis. It is an editorial decision if this is sufficient for publication. Also, I did not see any acknowledgements. **(1)** Was this work supported by a company, and on a related issue do any of the authors have any conflicts such as intellectual property or other commercial interests? This is not an issue but should just be stated. **(2)** Finally suggest that the writing be reviewed as it can be improved.

---

(1). The Core issue and comment 1:

"Was this work supported by a company, and on a related issue do any of the authors have any conflicts such as intellectual property or other commercial interests? This is not an issue but should just be stated."

Answer: Thank you very much. We believed that JT003 was first reported as the peptide agonist for both AdipoR1 and AdipoR2.

The 'conflicts of interest', 'author contributions' and 'acknowledgements' sections have added in page 15 in the revised manuscript.

The Core issue and comment 2：

"Finally suggest that the writing be reviewed as it can be improved."

Answer: Thank you very much. We have revised the manuscript, and carefully proof-read the manuscript to minimize typographical and bibliographical errors.

Other comments:

(2). The Core issue and comment 3：

"For example:

It should be anti-LOXL2 antibodies" instead of "LOXL2 antibodies."

Answer: Thank you very much. The "LOXL2 antibodies" has been revised with "anti-LOXL2 antibodies" in page 3, paragraph 3, line 9 in the revised manuscript.

(3). The Core issue and comment 4：

"Incorrect use of the word "affirm" many times."

Answer: Thank you very much. In page 4, paragraph 2, line 9, the word "affirmed" was revised with "confirmed". The line 13, "affirmed" was replaced by "demonstrated" in the revised manuscript.

(4). The Core issue and comment 5：

"Claiming that adiponectin has a "curative effect". It is of-course a potential therapy not a cure."

Answer: Thank you for your suggestion. In order to describe it more accurately, the word "curative" has been changed into "potential therapeutical" in page 12, paragraph 2, line 9 in the revised manuscript.

(5). The Core issue and comment 6：

"Also claimed that mitochondrial biogenesis was improved, but no real biogenesis data, only mitochondrial gene expression data."

Answer: Thank you very much. Herein, we have performed the mitochondrial content assay which is also one aspect to demonstrate the mitochondrial biogenesis (see **Fig. 4b** in the revised manuscript). On the other hand, the mitochondrial

biogenesis regulators PGC 1a and PG C1r3 expression were both accelerated with the dual agonist treatment (see **Fig. 4f, g** in the revised manuscript). Besides, the previous studies had implied that AMPK directly mediates the mitochondrial biogenesis (see reference 10). In summary, we could draw the conclusion that the mito chondrial biogenesis was improved.

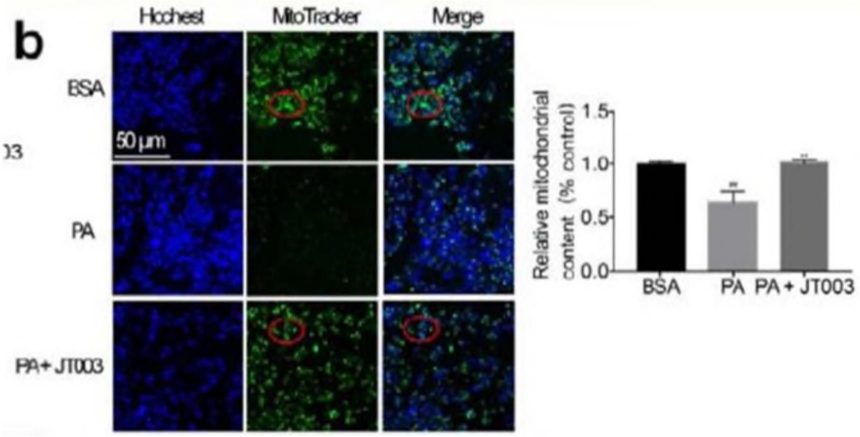

Fig. 4b in the revised manuscript: HepG2 cells of the indicated groups were stained with Mito tracker followed by assessment with m itochondrial content assay.

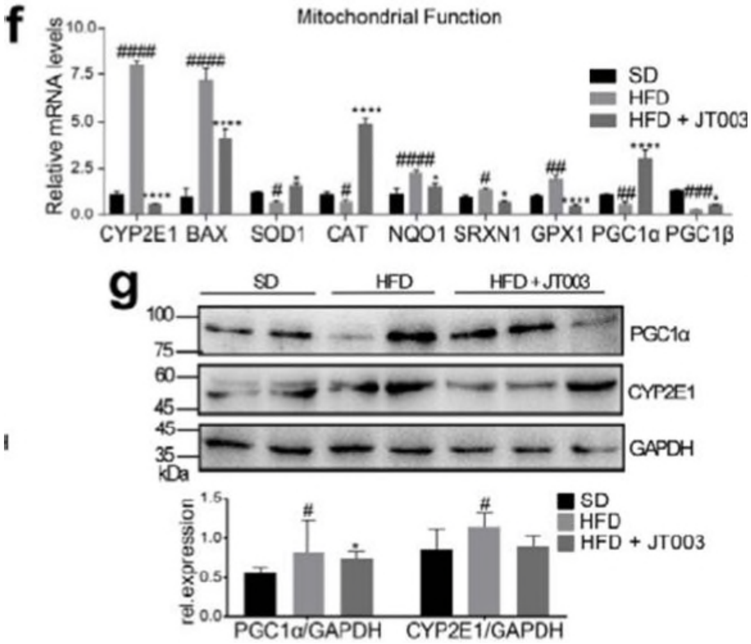

Fig. 4f, g in the revised manuscript: (f) mRNA transcription of the genes related t o ER stress and mito chondrial function in the liver samples of mice treated with SD, HFD or HFD plus JT 003 therapy. (g) The expression level of P GC1a and CYP2E1 in the indicated groups.

Reviewer: 2

In the manuscript by Xu, Jiang, et al., the authors describe the effects of a newly

developed peptide derived from adiponectin, which is an analog of the previously studied ADP355 peptide but with some improved pharmaceutical properties and suggested dual activity at both adiponectin receptors, to reduce the damaging effects of high-fat diet or chemical injury (CCl4) to liver health in mice. **(7)** As this is a pharmacology-focused manuscript, the experiments are lacking important controls and dose/concentration responses. **(8)**Furthermore, the authors fail to definitively show that this new analog (JT003) is indeed a dual agonist through specific binding or potency assays selective for the two receptor isoforms. Oligonucleotide-mediated knockdown of both receptor isoforms appears to blunt hepatocyte cellular binding of a labeled analog, and also altered transcriptional signatures in vitro. However, these experiments were not conducted with the individual knockdown of the receptor nor controls were not included. Thus, it is an incorrect statement to call this a dual agonist due to these deficiencies. **(9)**Without that dual agonism, other than an improved proteolytic stability by use of norvaline substitution, the new analog JT003 fails to distinguish itself from ADP355. Furthermore, a head-to-head comparison of JT003 to ADP355 at a few dose levels should have been completed, because ADP355 has previously been shown to improve fibrosis in CCl4-treated mice (Kumar et al. PLOS One 2014) and other models of liver injury (TAA; Wang et al. Scientific Reports 2016) and fibrosis (Marangoni et al. Scientific Reports 2017). Furthermore, the small molecule adiponectin receptor agonist (AdipoRon; Okada-Iwabu et al. Nature 2013) has also been shown to improve liver injury in mice (Wang et al. Eur J Pharm Sci 2016). By all accounts, this is the first test of these class of compounds in high-fat fed mice, which provides some novelty, but effects on body weight need to be reported differently and it is inaccurate to call this a "NASH model." This is a model of fatty liver disease with signs of inflammation but typically without a fibrotic liver. Thus, it is more accurate to call this a model of NAFLD, not NASH. **(10)**High fat diets combined with amino acid deficient diets (i.e. choline) have proven to be another model that manifests the human NASH condition of obesity and liver fibrosis thus should be considered here for study. Even many of the cellular mechanisms studied within (AMPK, PPARa, PPARγ, etc.) have already been attributed to the mechanism of action for activating the adiponectin receptors. Perhaps there is a degree of novelty in the proposed mechanism of inflammasome modulation and relation to ER stress, but this is just an aspect of the manuscript. **(11)**Therefore, to move the scientific community forward, a better understanding of how this molecule differs from predecessor

molecules is necessary. However, without standard pharmacological measures, comparisons, and controls in the current data set, combined with a restricted novelty and deficiencies in the presentation of the material (figures and written text), it is recommended to not publish the article in its current state.

(7). The Core issue and comment 7 :

"As this is a pharmacology-focused manuscript, the experiments are lacking important controls and dose/concentration resp onses."

Answer: Thank you very much. Firstly, We did think about the controls and dose/concentration responses, however, we only chose the optimum concentration of JT003 to further conduct the mechanism studies. Thanks to your valuable suggestions, we then ran a series of follow-up experiments which had ADP355 peptide as the controls and different concentrations of both ADP355 and JT003 are performed to illustrate the dose/concentration responses. Please see figure S3 and figure S4 in the revised supplementary information.

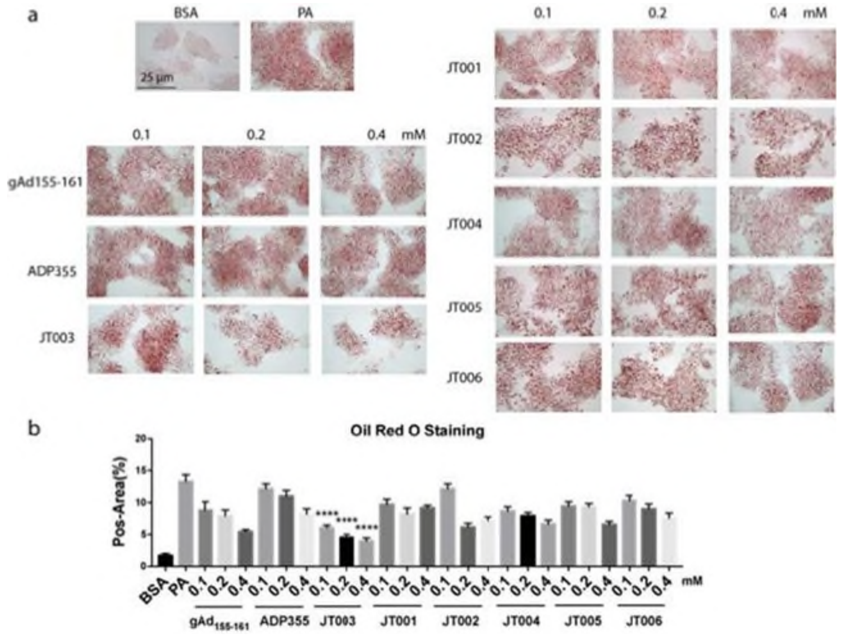

Figure S3. in the revised supplementary information: (a) Oil red o staining of PA induced lipid accumulation in He pG2 cell line after treated with different concentration of peptides. Triplicates were performed. (b) Positive areas were analyzed with I mageJ.

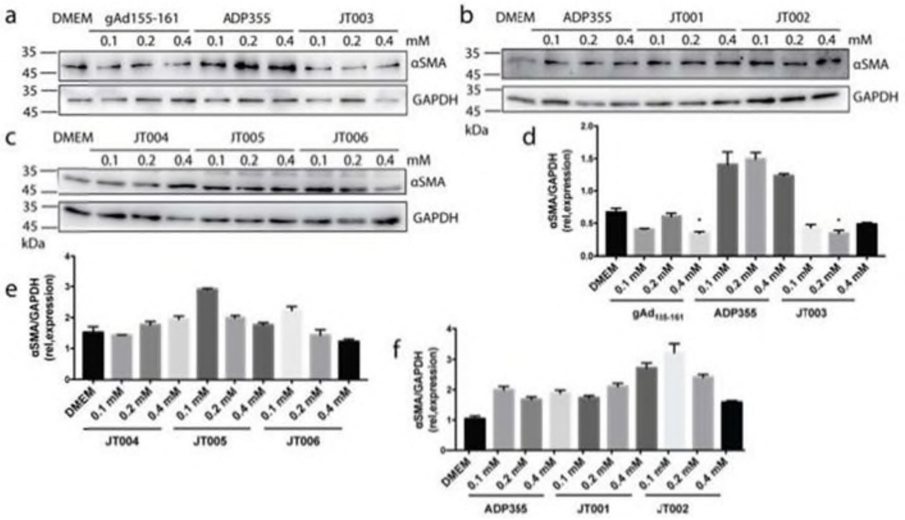

Figure S4. in the revised supplementary information: (a - c) Western blotting for aSMA expression in LX2 after peptides treated. Three separated experiments were performed. (d - f) aSMA expression was normalized to that of GAPDH.

(8). The Core issue and comment 8:

"Furthermore, the authors fail to definitively show that this new analog (JT00 3) is indeed a dual agonist through specific binding or potency assays selective for the two receptor isoforms. Oligonucleotide-mediated knockdown of both receptor isoforms appears to blunt hepatocyte cellular binding of a labeled analog, and also altered transcriptional signatures in vitro. However, these experiments were not conducted with the individual knockdown of the receptor nor controls were not ncluded. "

Answer: Thank you very much for your comments. We proposed that this new analog JT003 is a dual agonist of both AdipoR1 and AdipoR2. Because JT003 could indeed specifically bind to the two receptor isoforms. Oligonucleo tide-medi ated knockdown of both receptor isoforms blunt h epatocyte and hepatic stellate cells binding of a labeled analog, and also altered transcriptional signatures in vitro. Thanks to your valuable comments, we herein have conducted with the individual knockdown of the receptor and the controls were also included (Page 6, paragraph 2, line 5 and figure 1 d in the revised manuscript as well as figure S6 in the revised supplementary information). The individual knockdown of the two receptors experiments and JT006 as the control were performed to support the proposal. Our results indicated indeed that JT003 could bind to the two receptor isofo rms

specifically.

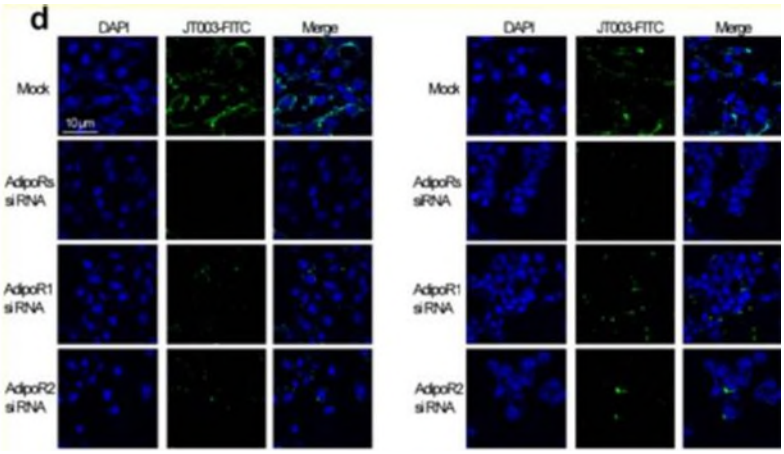

Fig. 1d in the revised manuscript: (d) HepG**2** and LX2 **c**ells were incubated with FIT**C**-JT003 at **3**7 $_{o}$C for 2 or 4 h. Th**e** nucleus **w**as staine**d** with Hoc**h**est33342. The gre**e**n channel represents as FITC. Fo**r** each exp**e**riment, tri**p**licates we**r**e perform**e**d.

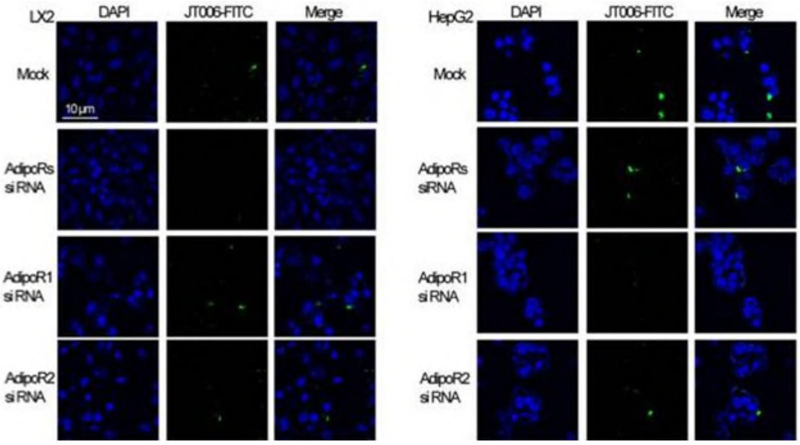

Figure S6. in the revised supplementary information: HepG2 and LX2 cells **w**ere incu**b**ated wit**h** FITC-JT0**0** 6 at 37 ^{o}**C** for 2 or 4 h. The **n**ucleus wa**s** stained with Ho**c**hest33342**.** The gre**e**n channe**l** represe**n**ts as FIT**C**. For eac**h** experi**m**ent, triplicates wer**e** performe**d**.

(9). The Core issue and comment 9:

"Wi thout that dual agonism, other than an improved pro teolytic stability by use of norv aline substitution, the new analog JT003 fails to distinguish itself from ADP355. Furthermore, a head-to-head comparison of JT003 to ADP355 at a few dose levels should have comple ted."

Answer: Man**y** thanks to your valuable sugges**t**ions, we have condu**c**ted a seri**e**s of foll**o**w-up exp**e**riments **w**hich had **A**DP355 pe**p**tide as the control**s** and different concentration**s** of both ADP355 and JT00**3** are per**f**ormed to illustrate the dose/concentr**a**tion respo**n**ses. Please see fig**u**re S3 and figure S4 in the re**v**ised

supplementary information, which are also presented in the former comments.

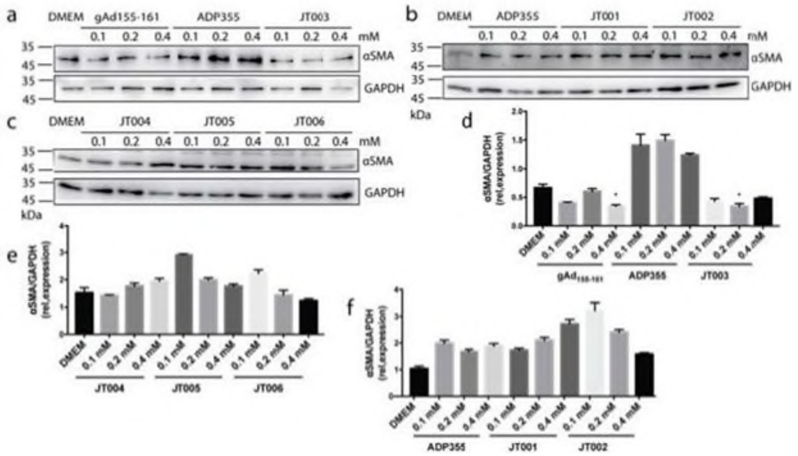

Figure S3. in the revised supplementary information: (a) Oil red **o** staining **of** PA induced lipid accumulation in He pG2 cell line after treated with different concentration of peptides. Triplicates were performed. (b) Positive areas were analyzed with I mageJ.

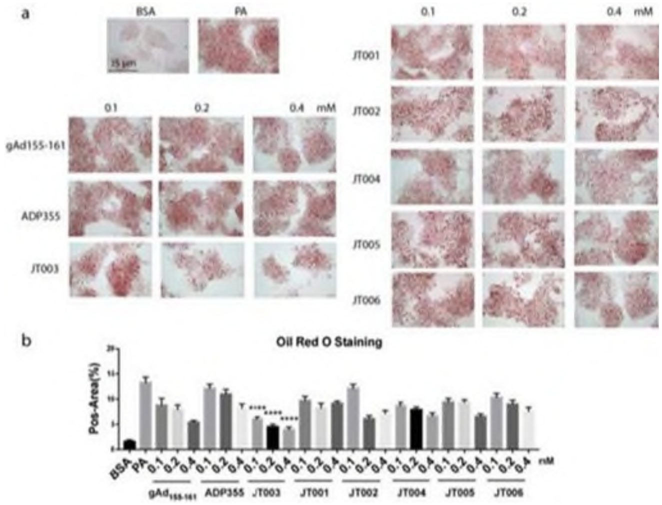

Figure S4. in the revised supplementary information: (a - c) Western blotting for aSMA expression in LX2 after peptides treated. Three separated experiments were performed. (d - f) aSMA expression was normalized to that of GAPDH.

(10). The Core issue and comment 10:

"High fat diets combined with amino acid deficient diets (i.e. choline) have proven to be another model that manifests the human NASH condition of obesity and liver fibrosis thus should be considered here for study."

Answer: Thank you very much for your comments. Nonalcoholic fatty liver disease occurs in the context of over-nutrition, particularly when associated with

central obesity, adipose dysfunction and inflammation and insulin resistance. Its pathological spectrum encompasses simple steatosis, nonalcoholic steatohepatitis (NASH) and pericellular fibrosis that can slowly lead to cirrhosis. High fat diet induced fatty liver disease animal models are widely used in the drug development researches. Thus, high fat diet induced NASH mouse model was used in the current study. However, according to the reviewer's suggestion, we also conducted high fat diet combine with methionine-choline deficient (MCD) diet to manifest the human NASH condition of obesity and liver fibrosis, further histological studies were performed as well. Besides, we also performed the comparison experiment to declare JT003 differs from predecessor molecules. In the revised manuscript, page 22, paragraph 3, line one, "HFD" was replaced with "NAFLD". Line 5, "The NASH model was conducted with feeding a HFD for 16 weeks combine with a MCD diet (Methionine/Choline Deficient Diet, Medicience, China) in the last 4 weeks. Mice administered a MCS diet (Methionine/Choline Sufficient Diet, Medicience, China) serves as control group. NAFLD or NASH mice" was added. Also. please see figure S9 and figure S10 in the revised supplementary information.

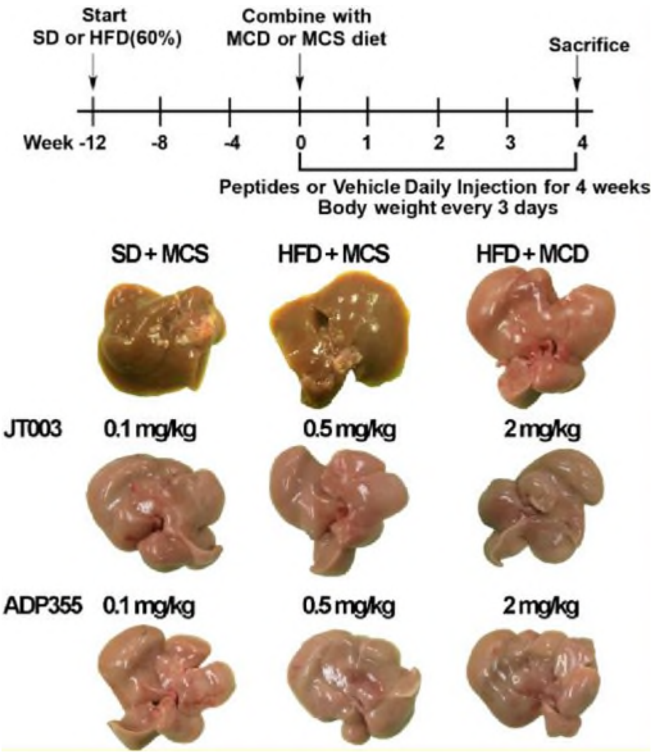

Figure S9. in the revised supplementary information: Schedule of HFD combine with MCD diet induced NASH and JT003 as well as ADP355 therapy. The bottom panel

showed the representative photographs of livers. Here and later, for each assay, three separated experiments were performed. For each experiment, n = 6.

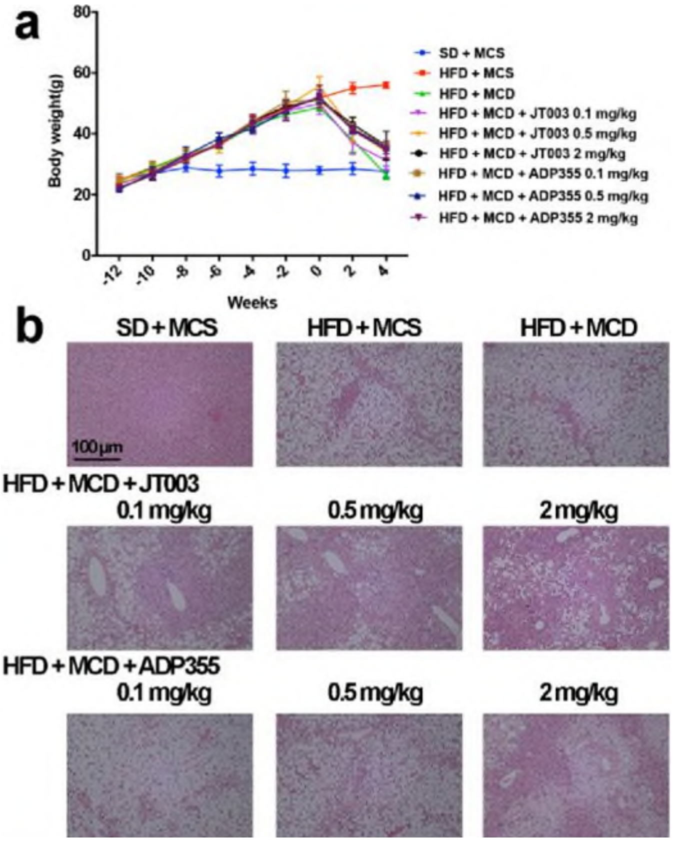

Figure S10. in the revised supplementary information: (a) Weight gain of mice treated with SD + MCS, HFD + MCS, HFD + MCD or JT003 as well as ADP355 therapy. (b) Representative images of HE staining of liver sections on mice of indicated groups.

(11). The Core issue and comment 11：

"Therefore, to move the scientific community forward, a better understanding of how this molecule differs from predecessor molecules is necessary. However, without standard pharmacological measures, comparisons, and controls in the current data set, combined with a restricted novelty and deficiencies in the presentation of the material (figures and written text)."

Answer: Thank you very much for your comments. The new analog JT003 was a target designed peptide which could bind specifically to AdipoR1 and AdipoR2, which is certainly different from any other predecessor peptides we discussed before. Besides, with a series of verification experiments both *in vitro* and *in vivo*, we could draw the conclusion that JT003 is a promising molecule that could be an effective

therapeutic on treating NA SH and related fibrosis. In addition, we also conducted the experiment of comparing the competitive binding between JT003 and globular adip onectin, which also demonstrate that JT003 could bind to Adipo Rs specifically. Please see figure 8g, h in the revised manuscript. Additionally, Kd value was determined via homogeneous time resolved fluorescence (HTRF) experiment. These results suggested that JT003 could bind to both Adipo R1 and A dipoR2, which indicated that JT003 is a newly designed dual agonist of Ad ipoRs, also in accordance with our *in vivo* results. Please see figure 8i in the revised manuscript.

g

h

i

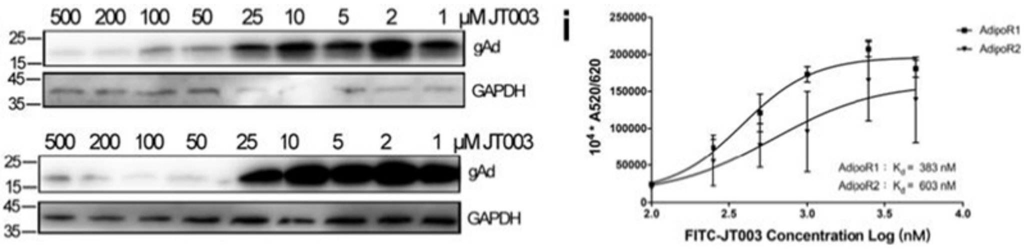

Figure 8. in the revised manuscript: Competitive binding of JT003 to globular adip onectin (gA d) AdipoR 1 overexpre ssion cells (g) and AdipoR2 overe xpression cells (h). (i) The Kd value of JT003 bind to A dipoR1 and AdipoR2.

Also, the presentation of the materials (figures and texts) are also strictly revised. The sentence " Lipid droplets accumulation caused by PA could be reduced by the native $gAd_{155-161}$ peptide and the analogues we designed[29], of which JT003 exhibited the best potential (Fig. 1b)." was revised with "L ipid droplets accumulation caused by PA could be reduced by the native $gA_{d155-161}$ peptide and the analogues we designed as well as previously reported peptide ADP355 [28, 29], of which JT003 exhibited the best potential (Fig.1 b, Supplementary Fig. 3)." Page 6, paragraph 1, line 1, " Supplemen tary Fig. 4" was added in the revised manuscript.

Page 6, paragraph 3, line 6 and supplementary Figure 7, the body weight has been reported. "Treatm ent with JT003 significantly smoothed the liver and decreased the liver sizes (Supplementary Fig. 5)." was replaced with "T reatment with JT003 significantly reduced the weight gain, smoothed the liver and decreased the liver sizes (Fig. 2a, Supplementary Fig. 7) in the revised man uscript."

Major and Minor Specific Comments

(12). The Core issue and comment 12:

"He ad-to-head in vivo profiling of JT003 versus ADP355 (at multiple dose levels) is vital for understanding if JT003 is indeed a superior molecule and should be done in

the HFD and CCl4 model herein, but also considered in amino acid deprived mouse models of NASH."

Answer: Thank you very much. We performed the head-to-head in vivo analysis to compare JT003 with ADP355 at multiple dose levels both *in vitro* and *in vivo*, in HFD and CCl_4 models, as well as HFD combine with MCD diets. Supplementary figure 3, 4, 8, 9, 10 and 12 were revised accordingly.

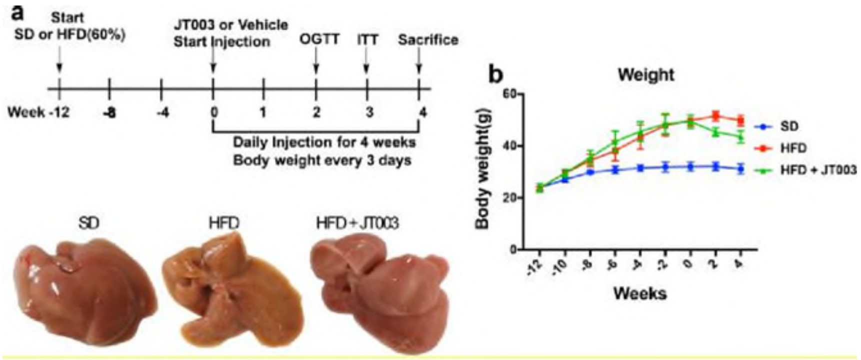

Figure S8. in the revised supplementary information: (a) Schedule of HFD induced NASH and JT003 therapy. The bottom panel showed the representative photographs of livers. (b) body weight changes during HFD and HT003 treatment.

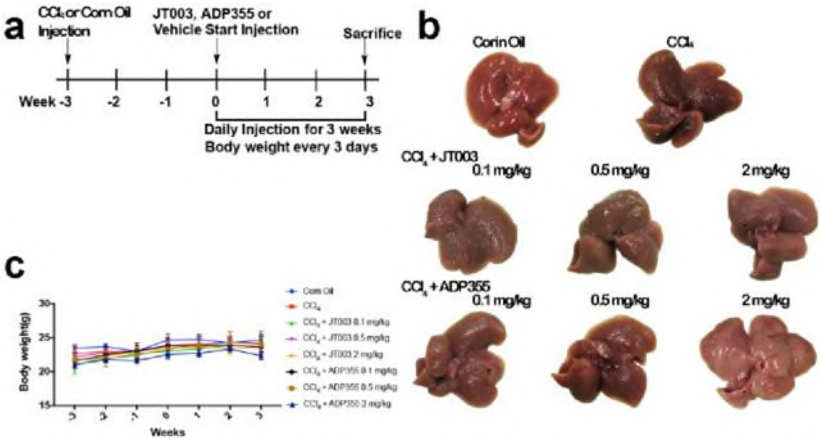

Figure S12. in the revised supplementary information: (a) Schedule of CCl_4 induced liver fibrosis and JT003 as well as ADP355 therapy. (b) The representative photographs of livers. (c) Body weight changes.

(13). The Core issue and comment 13:

"For the in vitro data confirming activity of the analogs, a rank order was generated based on single concentration experiments like in Figure 1B. As this is a pharmacological study, a concentration dependent association is important. Can the authors include concentrations higher and lower than 200 uM? Also, a comparison to native adiponectin is warranted."

Answer: Thank you very much. In Supplementary figure 3 and 4, various doses were conduct to illustrate the concentration dependent association. Instead of native adip onectin, a comparison to ADP355 has also been performed.

(14). The Core issue and comment 14:

"In Figure 1D, the authors show that fluorescently labeled JT003 binds to HepG2 and LX2 cells, and then fluorescent signal is attenuated with knockdown of Adip oR1 and R2. Can the authors provide activity data for the flu orescently labeled JT003 analog? Can the authors provide data showing FITC-lab eling of a molecule with weak affinity for R1/R2 shows less signal? This could be JT006, which showed the least aSMA signal (Figure 1C) and little prevention of palmitic acid induced lipid droplets (Figure 1B). The sequence scrambled peptide from Ma. et al. 2017 (peptide 56) could also be used as a control. Can the authors test for biological effects or cellular staining in cells that inherently do not express the receptors? These are important controls as there is a lack of pure binding/affinity data for the peptides to the two receptor isoforms, nor are there robust potency assays utilized."

Answer: Thank you for your valuable advice very much. The previous screening studied have showed us already JT003 was the most potential peptide which could ameliorate NA SH and related fibrosis. We thought maybe it is not necessary to synthesise all the fluorescent labeled analogues. However, we did provide the activity data for the fluorescently labeled JT006, which showed the least aSMA signal and little prevention of palmitic acid induced lipid droplets, in supplementary figure 6.

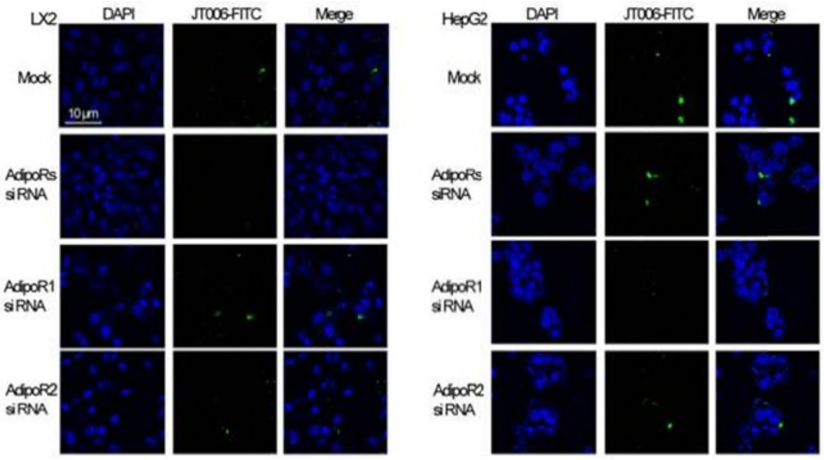

Figure S6. in the revised supplementary information: HepG2 and LX2 cells were incubated with FITC-JT00 6 at 37 °C for 2 or 4 h. The nucleus was stained with Hoc hest33342. The green channel represents as FITC . For each experiment, triplicates were performed.

(15). The Core issue and comment 15：

"Can the authors plot the body weight change from baseline/start of treatment (in grams or percentage) during the 4 weeks treatment of DIO mice shown in Figure 2A? Body weight was measured 3 times per week according to the methods. The statistical significance indicators need to be defined in the figure legend. Without the longitudinal body weight data, it cannot be determined if there is significant difference in the body weight following treatment, which the authors claim to be different from vehicle treated HFD controls. Please indicate the dose of JT003 (500 ug/kg) used either in the figure legend or depicted on the graphs themselves. How was this dose selected? Again, as this is a pharmacology study, a dose titration in the HFD mice should be included. Furthermore, it is not correct to call this a NASH model. This is a model of fatty liver or NAFLD since fibrosis is not typically evident in these animals. Sirius Red staining should be assessed in the livers to determine amount of fibrosis."

Answer: Thank you very much for your suggestions. The body weight change from the start of the treatment during the 4 weeks treatment of DIO mice has been showed in Figure 2a. And the body weight change from baseline was presented in supplementary figure 8. The dose of JT003 was described in the figure legend. We chose this dose due to some preliminary experiment which we didn't mention in the article. Anyway, various doses were conducted in the supplementary figure 9 and 10 of this revised study. Sirius Red staining has been assessed in the livers to determine amount of fibrosis, which are presented in CCl_4 induced fibrosis mouse model.

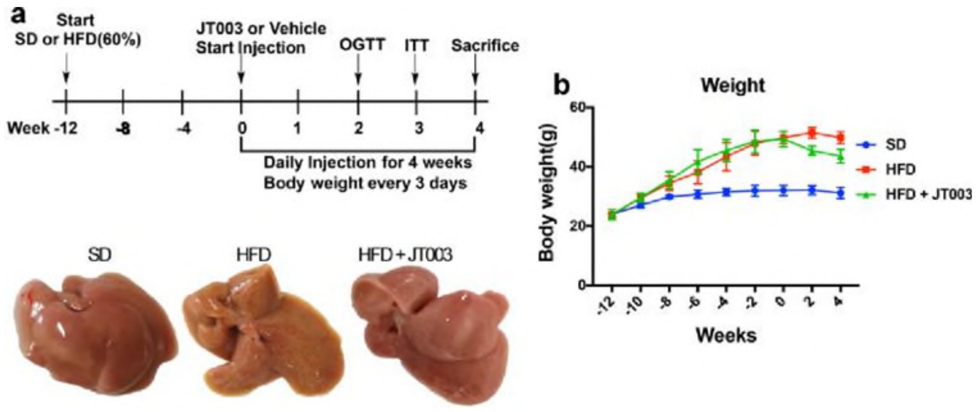

Figure S8. in the revised supplementary information: (a) Schedule of HFD induced NASH and JT003 therapy. The bottom panel showed the representative photographs of livers. (b) body weight changes during HFD and HT003 treatment.

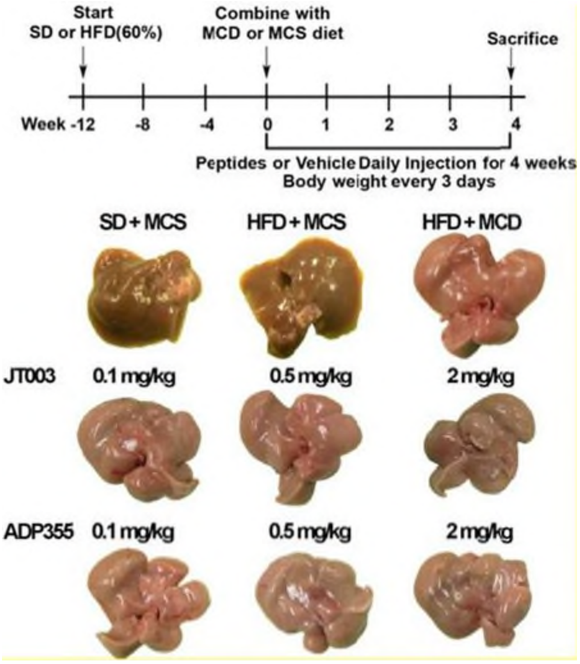

Figure S9. in the revised supplementary information: Schedule of HFD combine with MCD diet induced NASH and JT003 as well as ADP355 therapy. The bottom panel showed the representative photographs of livers. Here and later, for each assay, three separated experiments were performed. For each experiment, n = 6.

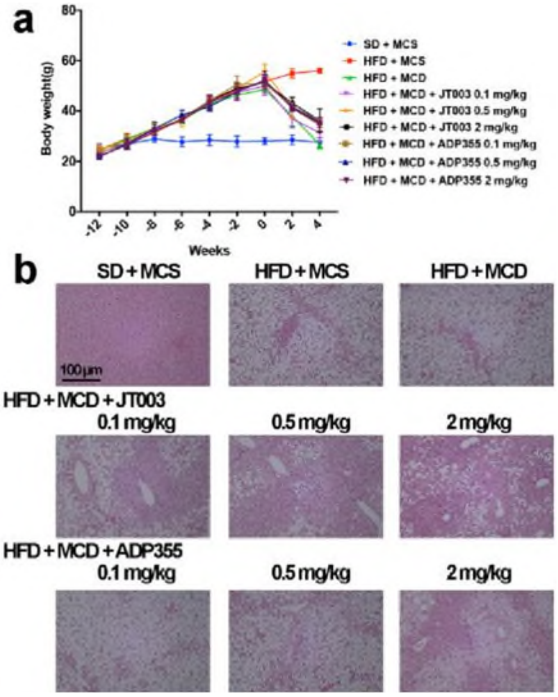

Figure S10. in the revised supplementary information: (a) Weight gain of mice treated with SD + MCS, HFD + MCS, HFD + MCD or JT003 as well as ADP355 therapy. (b) Representative images of HE staining of liver sections on mice of indicated groups.

(16). The Core issue and comment 16：

"The ALT data in Figure 2A should be a separate, independent panel within the figure. Similar for the adiponectin and insulin measures depicted on the right in Figure 28."

Answer: Thank you very much for your detailed advices. The ALT data and adiponectin and insulin measures have been separated within the figure 2 in the revised manuscript.

(17). The Core issue and comment 17：

"The authors state in the results "the increase in circulating adiponectin in the HFD group was reverted remarkably after JT003 treating." However, the data in Figure 28 shows that adiponectin levels are increased following JT003 treatment relative to vehicle HFD controls. Either there is a typo in the written results or there is a misunderstanding of the data. Please comment."

Answer: Thank you very much. Really sorry about that typo in the written results. In page 6, paragraph 2, line 9, the word "increase" has been revised as "decrease".

(18). The Core issue and comment 18：

"It is unclear what concentration of JT003 was used in Figure 3 as it is not in the legend nor depicted on the graphs. It is assumed it was 200 uM like in Figure 1. Please clarify."

Answer: Thank you very much. The concentration of JT003 used in Figure 3 is 200 μM as it is in Figure 1. It has been added in the figure legend. Please see page 29, line 14, the sentence "For in vitro experiment, the concentration of JT003 is 200 μM. For the in vivo experiment, the dose of JT003 is 500 μg/kg." was added in the revised manuscript.

(19). The Core issue and comment 19：

"The individual graphs in Figure 3D should be separated into individual panels."

Answer: Thank you very much. The figures in figure 3D have been separated into individual panels.

(20). The Core issue and comment 20：

"The authors claim that phosphorylated AMPK is increased following JT003 treatment in Figure 3F. However, the western blots do not support this claim nor does that ratio. Furthermore, the western blots for PPARa also do not support the claims, or at least suggest that a single lane/mouse is skewing the results to suggest PPARa protein is increased. Please comment. Overall, the western blots,

also in Figure 4, are variable and thus an accurate assessment of the quantification requires an examination of other blots. Particularly worrisome are the western blots for phosphorylated targets where the protein itself seems to by modulated by diet and or treatment."

Answer: Thank you very much. At first we do not avoid the variation during the experiment. However, we had 6 mice for each group. In the revised manuscript, we examined them all and give out the most appropriate results. Page 28, figure 3 was revised in the revised manuscript.

(21). The Core issue and comment 21：

"In the CCl4 mouse model (Figure 5), can the authors report body weight curves throughout treatment. Can the authors have the ALT measures as a separate panel within the figure? Again, the effects of different doses of JT003 should be studied."

Answer: Thank you very much. The body weight curves have been reported in supplementary figure 12 and 13. The ALT measures was presented as a separate panel in figure. Also, the effects of different doses of JT003 have been studied in the revised manuscript. Please see supplementary figure 12 and 13.

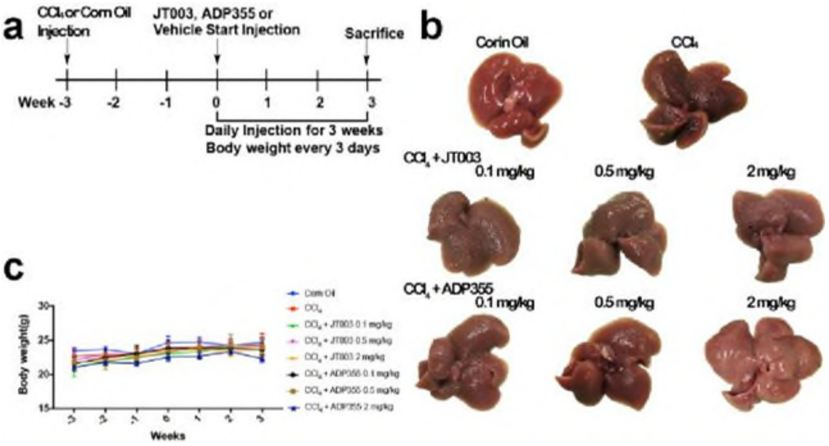

Figure S12. in the revised supplementary information: (a) Schedule of CCl_4 induced liver fibrosis and JT003 as well as ADP355 therapy. (b) The representative photographs of livers. (c) Body weight changes.

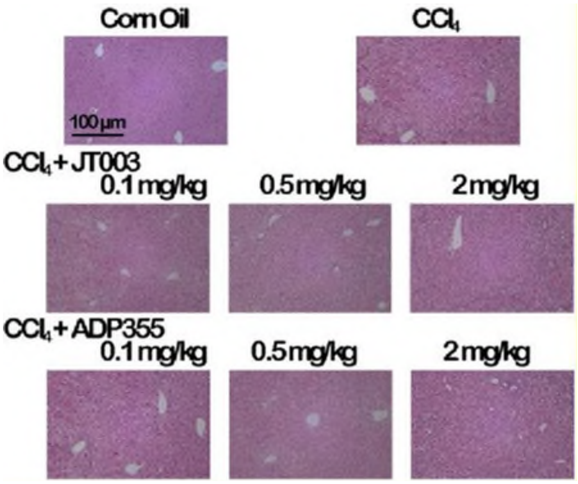

Figure S13. in the revised supplementary information: Representative images of HE staining of liver sections on mice of indicated groups.

(22). The Core issue and comment 22:

"The authors choose words such as "ameliorated" and "alleviation" which suggest that the pathological effects were completely reversed with JT003 treatment. However, that is not the case so the authors should use words such as "reduced" or "improved." Furthermore, the statement of curative effects in the discussion should be tempered as the disease was not cured in these mice, nor is this the first report of adiponectin peptides demonstrating effects on liver metabolism."

Answer: Thank you very much. Page 2, paragraph 1, line 5, and page 4, paragraph1, line 14, "ameliorated" were revised with "improved". Page 9, paragraph 3, line 5, "ameliorated" was replaced by "reduced". Page 10, paragraph 2, line 7, "ameliorated" were altered with "improved". Page 11, paragraph 1, line 4, "ameliorated" were revised with "decreased". Page 11, paragraph 1, line 13, "ameliorated" were replaced with "lessened". Page 14, paragraph 1, line 4, "ameliorated" was swapped with "reduced". Page 9, paragraph 3, line 8, "alleviation" was changed with "improvement". Page 14, paragraph 1, line 5, "alleviation" was revised by "decreasing".

(23). The Core issue and comment 23:

"Overall, the figures are hardly legible. Please increase the size of the symbols and decrease the size of the error bars for the graphed data."

Answer: Thank you very much. The size of the symbols and the error bars are all increased. Please see the figures.

(24). The Core issue and comment 24:

“The text requires significant proof-reading and revision as there are numerous grammatical m istakes.”

Answer: Thank you very much. According to your comments, we carefully proof-read our manuscript and had these mistakes revised in the revised manuscript.

In page 15, paragraph 2, line 4, “ N.S., S.W. and S.C. performed the animal experiments and the acquisition of d ata.” was revised with “N.S. and S.W. performed the animal experiments and the acquisition of d ata.” Line 5, “R. L and L.J. performed the binding affinity assay.” was added.

Reviewer: 3

Xu et al. evaluated positive effects of a peptide JT003 on NASH/ fibrosis model. Overall effects of it coincide with ad iponectin function on NASH model studied by using adiponec tin KO mice. AMPK and PPARa signaling may also well coincide with globular adipo nectin-AdipoRs signaling. **(25)** However, there is no experiment indicating the binding of JT003 to AdipoRs, nor competitive binding of JT003 to adiponectin, ideally native one in serum/plasma, or at least globular form of adiponectin, which was initially shown to bind AdipoRs. Such experimental evidences to show that this peptide truly function through binding to AdipoRs are necessary.

(25). The Core issue and comment 25：

“Ho wever, there is no experiment indicating the binding of JT003 to AdipoRs, nor competitive binding of JT003 to adiponectin, ideally native one in serum/plasma, or at least globular form of adiponectin, which was initially shown to bind AdipoRs. Such experimental evidences to show that this peptide truly function through binding to AdipoRs are ne cessary.”

Answer: Thank you very much for your valuable comments. The competitive binding of JT003 to globular form of adip onectin has been performed in the revised manuscript. Furthermore, we have conducted the HTRF experiment to calculate the EC_{50} of JT003. Please see Figure 8.

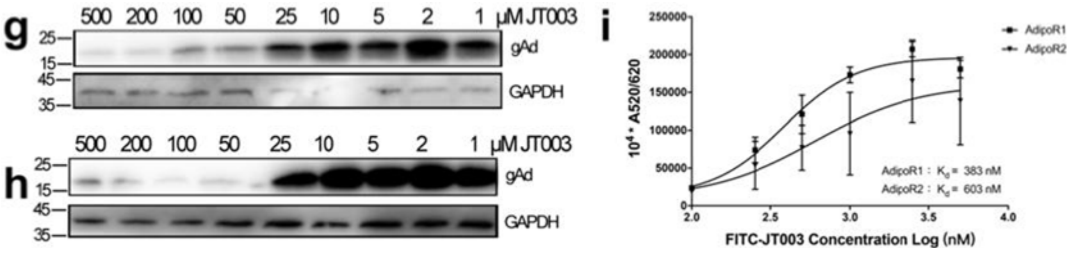

Figure 8. in the revised manuscript: Competitive binding of JT003 to globular

adiponectin (gAd) AdipoR1 overexpression cells (g) and AdipoR2 overexpression cells (h). (i) The Kd value of JT003 bind to AdipoR1 and AdipoR2.

For mechanism comment from Reviewer: 2

Furthermore, the authors fail to definitively show that this new analog (JT003) is indeed a dual agonist through specific binding or potency assays selective for the two receptor isoforms. Oligonucleotide-mediated knockdown of both receptor isoforms appears to blunt hepatocyte cellular binding of a labeled analog, and also altered transcriptional signatures in vitro. However, these experiments were not conducted with the individual knockdown of the receptor nor controls were not included.

Answer point by point: In the revised manuscript, we have fully addressed this comment raised by the reviewer-2 through performing the required experiments. The details are as follows:

We proposed that this new analog JT003 is a dual agonist of both AdipoR1 and AdipoR2. We herein have conducted with the individual knockdown of the receptor and the controls were also included (Page 6, paragraph 2, line 5 and figure 1 d in the revised manuscript as well as figure S6 in the revised supplementary information). The individual knockdown of the two receptors experiments and JT006 as the control were performed to support the proposal. Our results indicated indeed that JT003 could bind to the two receptor isoforms specifically.

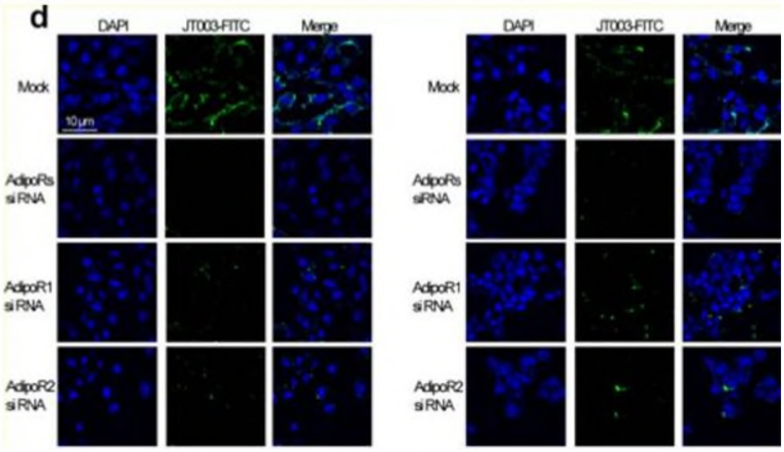

Fig. 1d in the revised manuscript: (d) HepG2 and LX2 cells were incubated with FITC-JT003 at 37 oC for 2 or 4 h. The nucleus was stained with Hochest33342. The green channel represents as FITC. For each experiment, triplicates were performed.

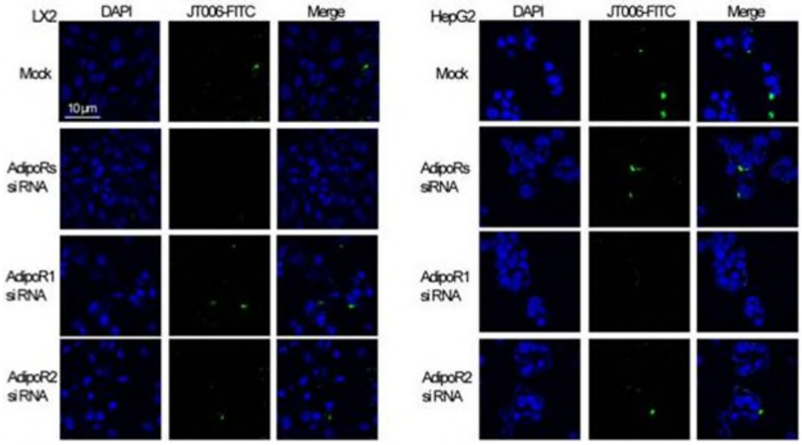

Figure S6. in the revised supplementary information: HepG2 and LX2 cells were incubated with FITC-JT00 6 at 37 ^{o}C for 2 or 4 h. The nucleus was stained with Hochest33342. The green channel represents as FITC. For each experiment, triplicates were performed.

For mechanism comment from Reviewer: 3

However, there is no experiment indicating the binding of JT003 to AdipoRs, nor competitive binding of JT003 to adip onectin, ideally native one in serum/plasma, or at least globular form of a diponectin, which was initially shown to bind AdipoRs. Such experimental evidences to show that this peptide truly function through binding to Adip oRs are necessary.

Answer point by point: In the revised manuscript, we have fully addressed this comment raised by the reviewer-3 through performing the required experiments. The details are as follows:

The competitive binding of JT003 to globular form of adipone ctin has been

per**f**ormed in **t**he revise**d** manuscri**p**t. Furthermore, we **h**ave cond**u**cted the **H**TRF experiment to calculate t**h**e $_{EC50}$ of J**T** 003. Pleas**e** see Figur**e** 8.

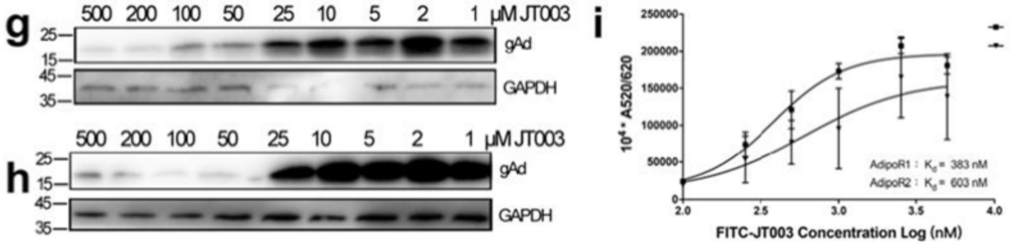

Figure 8. in the revised manuscript: Comp**e**titive binding of JT0**0** 3 to glo**b**ular adi**p** onectin (g**A** d) AdipoR 1 overexpr**e** ssion cells (g) and AdipoR2 over**e** xpression cells (h). (i) The Kd **v**alue of JT0 03 bind to **A** dipoR1 and AdipoR2.

Reviewers' Comments:

Reviewer #1:
Remarks to the Author:
The authors have addressed the issues and the manuscript is improved but I still have concerns about the originality of the work as it is reporting on the activity of a new bi-valent agonist for a pathway that is quite well worked out.

Reviewer #2:
Remarks to the Author:

The authors have done a commendable job in addressing many of limitations of the previous version of the manuscript. However, some limitations remain that prevent a conclusive interpretation of the results particularly in relation to if and why JT003 provides a step-change over predecessor analogs (ADP355) and the therapeutic potential of this strategy.

Most importantly, the authors have now included concentration-response results for JT003 vs ADP355 in the HepG2 lipid accumulation assay (Figure S3) and LX2 aSMA assay (Figure S4). Furthermore, the authors include a dose-response of JT003 vs ADP355 in the HFD+MCD (Figure S9) and CCl4 models (Figure S12) but stop short in a dose response in HFD mice, of which many subsequent conclusions are drawn. (1) Can the authors provide similar dose-response data in HFD mice (JT003 vs ADP355 at same dose levels in other models)? However, the current presentation of the dose-response data in the MCD and CCl4 studies do not allow for an accurate comparison because quantification was not included. (2) Can the authors generate a steatosis score, quantify steatosis (Oil Red O) and fibrosis staining (Sirius Red) in both models for a more accurate comparison can be gained? (3) Furthermore, the authors should articulate the results in the written text and emphasize the potency/efficacy difference of JT003 vs ADP355

(4) Can the authors perform competition binding experiments and the HTRF assay for ADP355 to determine whether the better in vivo performance due to altered receptor engagement or due to improved pharmacokinetics. (5) Further, can the authors perform HTRF assay for the FITC-labeled JT003 and determine if JT006 (used as negative control) has altered binding affinity. (6) Lastly, can globular adiponectin be used as a positive control in your HTRF assay.

(7) Can the authors perform a formal PK measure of JT003 vs ADP355 in mice?

Concentration ranges of 100 to 400 uM were used in the HepG2 and LX2 cells, which is 100-1000-fold higher than the reported Ka from the HTRF experiments. (8) Did the authors perform HepG2 and LX2 assays using concentrations that are closer to the reported Ka of JT003 at both receptors (~300-600 nM).

(9) Additionally, the values reported in Figure S3 should be normalized by cell number because the representative images shown do suggest a lower number of cells present in the JT003 treated group relative to the other groups. Alternatively, can the authors comment on the images indicating a potential decrease in cell number?

(10) What concentration of the FITC labeled peptides (JT003 and JT006) were used in the cell imaging experiments? In general, it should be more evident to the reader what concentrations of compounds were used in each experiment by either indicating it on the figure itself or place it in the figure legend.

Minor
The authors state that in the MCD model, JT003 demonstrated an "excellent ability of reducing

body weight and inhibiting lipid accumulation.” First, the control group of HFD + MCD causes body weight loss, so the weight loss cannot be attributed to the therapy. Second, claiming an “excellent ability” is not a quantifiable or scientific statement. The effects on lipid accumulation seem differential from control groups, and there does not seem to be a difference between ADP355 and JT003 in a dose-dependent manner. Lastly, the MCD model utilized here is in question whether a fibrotic state is present in only 4 weeks of amino acid deficiency as other data suggest many months of amino-acid deprivation is necessary to induce low-level fibrosis in C57Bl/6 mice (i.e. Wolf & Heikenwalder et al. Cancer Cell 2016).

The authors have added a statement in the introduction claiming “GLP-1R agonists did not show significant response to liver fibrosis.” There is no citation for this comment nor is there clinical data signifying a lack (or benefit) of GLP-1R agonists to alleviate liver fibrosis.

Reviewer #3:
Remarks to the Author:
Xu et al. evaluated the positive effects of a peptide JT003 on the NASH/fibrosis model. Overall effects of the compound coincide with adiponectin function on the NASH model studied by using adiponectin KO mice. AMPK and PPARalpha signaling may also well coincide with adiponectin-AdipoRs signaling previously supposed.
However, during the long revise period, it was reported that AdipoRs regulate membrane fluidity independent on adiponectin and thereby loss of them affects cellular signaling and metabolism (J Lipid Res. 2019 May;60(5):995-1004, Elife. 2019 Nov 26;8. pii: e47733). Therefore, it may be important to show the relationship between the modulation of membrane fluidity by the peptide agonist and AMPK/PPARalpha signaling with substantial experimental evidence. The interaction of native adiponectin existing in serum to AdipoRs was also reported not to exist (Elife. 2019 Oct 24;8. pii: e48675.). Therefore, it may be also important now to show the difference between native adiponectin existing in serum and bacterially produced gAd on the binding to AdipoRs overexpressed in cells. Such evaluation may leave pharmacological significance of this compound.
The manuscript contains somewhat strange presentations in bar-graphs with representing western blots as pointed out by reviewer#2 (The Core issue and comment 20). And such points were little revised by authors. Indeed, in Figure 8C, there can be seen a very intense band of PPARgamma in lane 7. However, in the corresponding graph, such high PPARgamma expression was not observed, with a very small error bar. The triplicate examination must have a large error bar in the bar-graph, if it contains such a highly different point.

Response to the Comments of Reviewers in Detail:

Reviewer: 1

The authors have addressed the issues and the manuscript is improved but I still have concerns about the originality of the work as **(1)** it is reporting on the activity of a new bi-valent agonist for a pathway that is quite well worked out.

----

(1). The Core issue and comment 1：

Answer: Thank you very much. Although AdipoR1 agonist such as ADP355 has been addressed, to date, the study of AdipoR1/AdipoR2 dual agonist has not been reported. Firstly, we believed that JT003 was first reported as the peptide agonist for both AdipoR1 and AdipoR2. Secondly, except for the classic adiponectin receptor pathways (based AdipoR1 agonist), we also presented that the JT003 treatment significantly improved ER-mitochondrial axis function, which contributed to reduce HSCs activation. We believe our work fully respect the originality of science research.

Reviewer: 2

The authors have done a commendable job in addressing many of limitations of the previous version of the manuscript. However, some limitations remain that prevent a conclusive interpretation of the results particularly in relation to if and why JT003 provides a step-change over predecessor analogs (ADP355) and the therapeutic potential of this strategy.

Most importantly, the authors have now included concentration-response results for JT003 vs ADP355 in the HepG2 lipid accumulation assay (Figure S3) and LX2 aSMA assay (Figure S4). Furthermore, the authors include a dose-response of JT003 vs ADP355 in the HFD+MCD (Figure S9) and CCl_4 models (Figure S12) but stop short in a dose response in HFD mice, of which many subsequent conclusions are drawn. **(2)** (1) Can the authors provide similar dose-response data in HFD mice (JT003 vs ADP355 at same dose levels in other models)? However, the current presentation of the dose-response data in the MCD and CCl4 studies do not allow for an accurate comparison because quantification was not included. **(3)**(2) Can the authors generate a steatosis score, quantify steatosis (Oil Red O) and fibrosis staining (Sirius Red) in both models for a more accurate comparison can be gained? **(4)**(3) Furthermore, the

authors should articulate the results in the written text and emphasize the potency/efficacy difference of JT003 vs ADP355.

(5)(4) Can the authors perform competition binding experiments and the HTRF assay for ADP355 to determine whether the better in vivo performance due to altered receptor engagement or due to improved pharmacokinetics. (5) Further, can the authors perform HTRF assay for the FITC-labeled JT003 and determine if JT006 (used as negative control) has altered binding affinity. (6) Lastly, can globular adiponectin be used as a positive control in your HTRF assay.

(6)(7) Can the authors perform a formal PK measure of JT003 vs ADP355 in mice? Concentration ranges of 100 to 400 uM were used in the HepG2 and LX2 cells, which is 100-1000-fold higher than the reported Ka from the HTRF experiments. (8) Did the authors perform HepG2 and LX2 assays using concentrations that are closer to the reported Ka of JT003 at both receptors (~300-600 nM).

(7)(9) Additionally, the values reported in Figure S3 should be normalized by cell number because the representative images shown do suggest a lower number of cells present in the JT003 treated group relative to the other groups. Alternatively, can the authors comment on the images indicating a potential decrease in cell number?

(8)(10) What concentration of the FITC labeled peptides (JT003 and JT006) were used in the cell imaging experiments? In general, it should be more evident to the reader what concentrations of compounds were used in each experiment by either indicating it on the figure itself or place it in the figure legend.

Minor

(9) The authors state that in the MCD model, JT003 demonstrated an "excellent ability of reducing body weight and inhibiting lipid accumulation." First, the control group of HFD + MCD causes body weight loss, so the weight loss cannot be attributed to the therapy. Second, claiming an "excellent ability" is not a quantifiable or scientific statement. **(10)** The effects on lipid accumulation seem differential from control groups, and there does not seem to be a difference between ADP355 and JT003 in a dose-dependent manner. **(11)** Lastly, the MCD model utilized here is in question whether a fibrotic state is present in only 4 weeks of amino acid deficiency as other data suggest many months of amino-acid deprivation is necessary to induce low-level fibrosis in C57Bl/6 mice (i.e. Wolf & Heikenwalder et al. Cancer Cell 2016).

(12) The authors have added a statement in the introduction claiming "GLP-1R

agonists did not show significant response to liver fibrosis." There is no citation for this comment nor is there clinical data signifying a lack (or benefit) of GLP-1R agonists to alleviate liver fibrosis.

---

(2). The Core issue and comment 2：

Answer: Thank you very much for your comments. We already included the dose-response of JT003 and ADP355 in both NASH (HFD + MCD) and fibrosis (CCl_4) models, it is adequate to evaluate the pharmacological effects of these peptides. For the single HFD mouse model, we emphasize on pathways and mechanism study, which is much more efficiency to use the single dose. Please see the steatosis area quantification for HFD+MCD mouse model in figure S10 and representative images of sirius red staining and indicated quantification for CCl_4 mouse model in figure S13 in the revised supplementary information, which is also showed below.

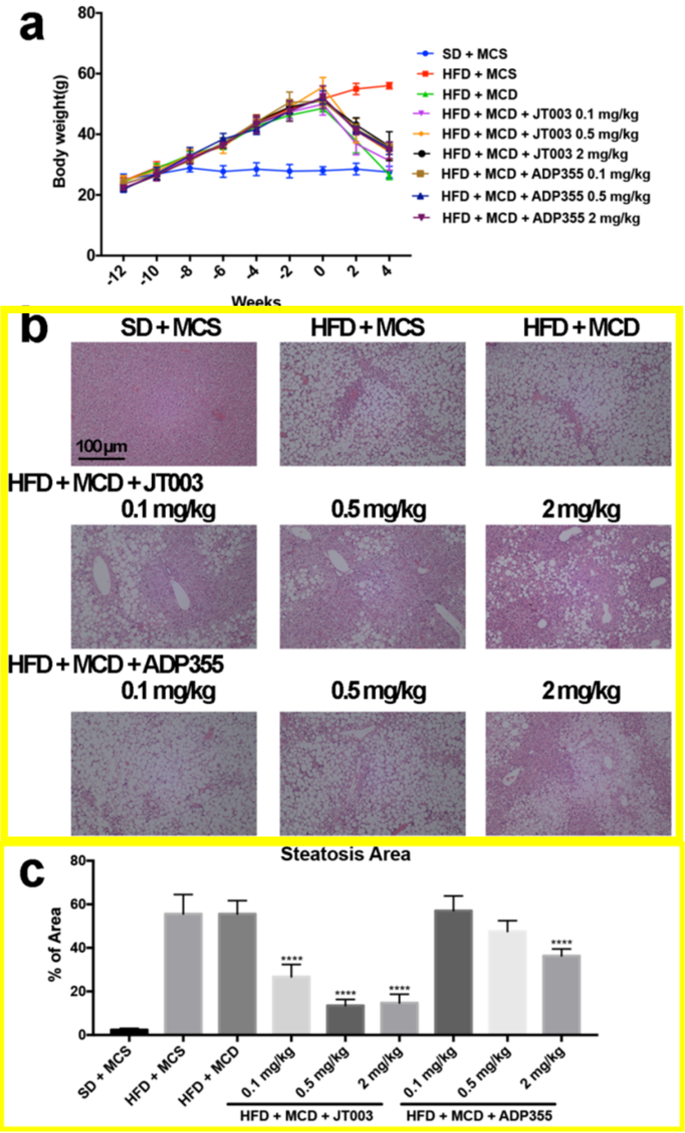

Figure S10. (a) Weight gain of mice treated with SD + MCS, HFD + MCS, HFD + MCD or JT003 as well as ADP355 therapy. (b, c) Representative images of HE staining of liver sections on mice of indicated groups and steatosis area. Positive areas were analyzed with ImageJ.

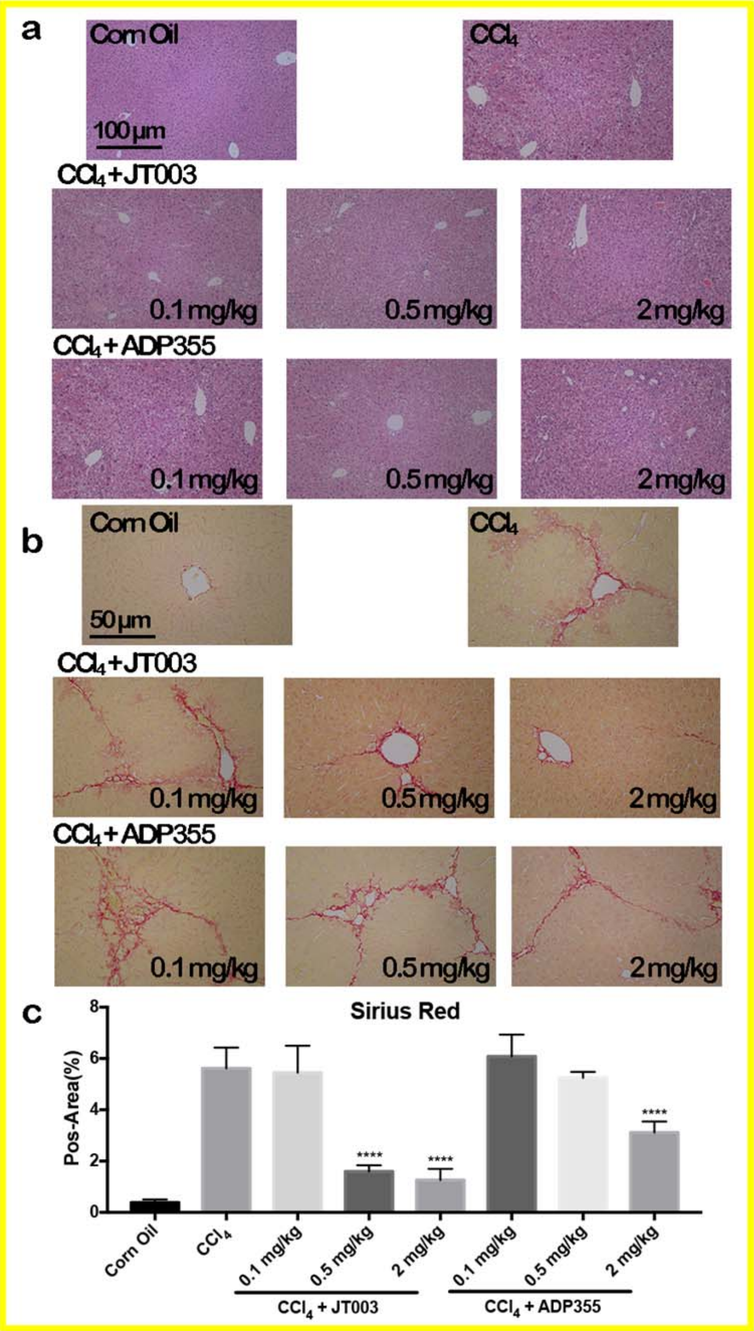

Figure S13. Representative images of HE (a) and Sirius red (b) staining of liver sections on mice of indicated groups. (c) Sirius red positive areas were analyzed with Image J.

(3). The Core issue and comment 3：

Answer: Thank you very much for your comments. We have responded to this comment in figure S10 and S13. The quantify steatosis (Oil Red O) and fibrosis staining in both models for a more accurate comparison was gained by Image J.

(4). The Core issue and comment 4:

Answer: Many thanks to your valuable suggestions. The description of the results in the written text has been further explained in the revised manuscript accordingly. In page 6, paragraph 2, line 8, the sentence "Besides, in HFD combine with methionine and choline deficient (MCD) diet NASH mouse model, compare to the previous reported peptide ADP355, JT003 exhibited excellent ability of reducing body weight and inhibiting lipid accumulation (Supplementary Fig. 9, 10)." has been revised to "Besides, in HFD combine with methionine and choline deficient (MCD) diet NASH mouse model, JT003 inhibited lipid accumulation significantly with a 20 times lower dosage than the previous reported peptide ADP355 (Supplementary Fig. 9, 10)."

In page 9, paragraph 2, line 6, "HE and sirius red staining assays revealed that the inflammation, necrosis and fibrosis area in CCl_4 induced liver fibrosis were decreased by JT003 treatment, and there is no significant improvement with ADP355 treatment (Supplementary Fig. 13, Fig. 5a)." was replaced by "HE and sirius red staining assays revealed that the inflammation, necrosis and fibrosis area in CCl4 induced liver fibrosis were significantly decreased by JT003 with the administration dosages of 0.5 mg/kg and 2 mg/kg. In contrast, these effects were not notably raised employing the same dosages of ADP355 (Supplementary Fig. 13 and Fig. 5a).".

(5). The Core issue and comment 5:

Answer: Thank you for your comments. The further HTRF assay experiments of FITC-labeled JT003, JT006 and ADP355 have been performed in the revised manuscript, please see the page 36, Fig. 8i. In HTRF assay, we detected the FITC fluorescence intensity, so we successfully labeled the JT003, JT006 and ADP355 with one FITC molecule with the method of solid-phase synthesis, respectively.

However, it is worth noting that as a protein, globular adiponectin could not be specifically labeled by FITC (It is a very difficult chemical and biological engineering to achieve specific labeling. Maybe it's an impossible task.). Even so, we have made great efforts and attempts to do it. We used the E.Coli to produce globular adiponectin, the fluorescence intensity would be much higher when we labeled this protein with FITC (because more FITC molecules non-specifically labeled it), it would

be not comparable to use multi-labeled globular adiponectin as a positive control.

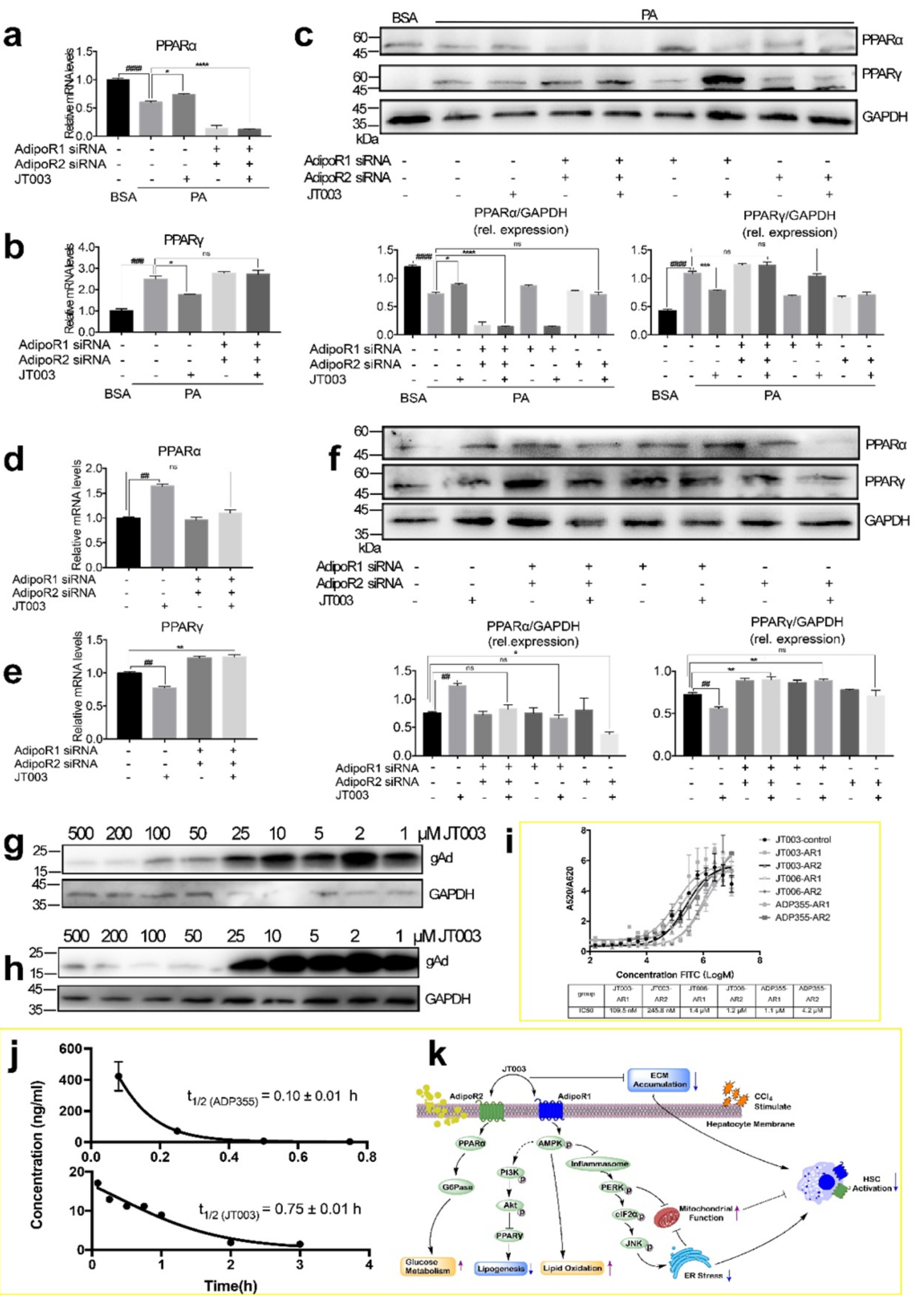

Fig.8 | Suppression of AdipoR1 and AdipoR2 blocks the downstream pathways activated by JT003. The mRNA and protein expression level of PPARα and PPARγ in HepG2 (a, b, c) and LX2 cells (d, e, f) of the indicated groups with AdipoR1 and/or AdipoR2 siRNA

treatment. Competitive binding of JT003 to globular adiponectin (gAd) AdipoR1 overexpression cells (g) and AdipoR2 overexpression cells (h). (i) The K_d value of JT003, JT006 and ADP355 bind to AdipoR1 and AdipoR2. (j) Plasma concentration versus time profiles of ADP355 and JT003 in SD rats following a single intravenous dose of 0.5 mg/kg and 1.0 mg/kg, respectively. (k) Scheme of AdipoR1/AdipoR2 dual agonist JT003 in recovering NASH and related fibrosis. JT003 binds to AdipoR1 and AdipoR2, improves glucose and lipid metabolism, suppresses HSCs activation and modulates the ER-mitochondria axis function in the development of NASH and related fibrosis.

The results indicated that compare to JT006 and ADP355, JT003 can bind to AdipoR1 and AdipoR2 in a significantly lower K_d value, determined that the better in vivo performance due to altered receptor engagement. At the same time, the pharmacokinetic study demonstrated that JT003 has more favorable pharmacokinetic properties than ADP355. After intravenous administration, both JT003 and ADP355 underwent biphasic elimination from the circulation, while the latter concentrations were measurable only for 45 minutes (Fig. 8k). Compared with ADP355 (6 minutes), JT003 possesses longer half-life, larger volume of distribution and higher clearance in SD rats (Supplementary Table. 4). This is also reflected by longer MRT value of JT003.

In summary, the above results indicated that the better *in vivo* performance of JT003 is comprehensive due to the receptor binding of lower K_d value, altered receptor engagement, and the longer half-life than ADP355.

(6). The Core issue and comment 6：

Answer: Thank you for your comments.

The formal PK measure of JT003 and ADP355 in rat was performed. Data were showed in Fig. 8j and Table S4. The pharmacokinetic study demonstrated that JT003 ($t_{1/2}$ = 0.75 h) has more favorable pharmacokinetic properties than ADP355 ($t_{1/2}$ = 0.1 h). Table S4 is showed below:

Parameter	JT003 (iv, 0.5 mg/kg)	ADP355 (iv, 1.0 mg/kg)

$t_{1/2}$ (h)	0.75 ± 0.01	0.10 ± 0.01
$AUC_{0\text{-}t}$ (h*ng/mL)	19.36 ± 1.51	134.01 ± 19.87
$AUC_{0\rightarrow\infty}$ (h*ng/mL)	20.85 ± 1.63	134.45 ± 19.91
V (L/kg)	25.96 ± 2.29	1.06 ± 0.21
CL (L/h/kg)	24.08 ± 1.92	7.54 ± 1.04
MRT (h)	1.04 ± 0.03	0.09 ± 0.01

Table S4. Pharmacokinetic parameters of JT003 and ADP355 in SD rats. Pharmacokinetic parameters were calculated from plasma concentration-time data using non-compartmental analysis. The results were reported as Mean ± SD for n = 3-6.

The results were described in Page 12, paragraph 2, “Besides, the pharmacokinetic study demonstrated that JT003 has more favorable pharmacokinetic properties than ADP355. After intravenous administration, both JT003 and ADP355 underwent biphasic elimination from the circulation, while the latter concentrations were measurable only for 45 minutes (Fig. 8k). Compared with ADP355, JT003 possesses longer half-life, larger volume of distribution and higher clearance in SD rats (Supplementary Table. 4), which may be attributed to its lipophilic nature. This is also reflected by longer MRT value of JT003. These results suggested that JT003 could bind to both AdipoR1 and AdipoR2 with longer half-life, which indicated that JT003 is a newly designed, more efficiency dual agonist of AdipoRs, also in accordance with our *in vivo* results.” was added.

Method section, page 26, paragraph 1, “Pharmacokinetic assay Male SpragueDawley (SD) rats were randomly divided into two groups (n = 3 per group) and received a single intravenous dose of JT003 (0.5 mg/kg) or ADP355 (1.0 mg/kg) respectively. Blood samples were collected at 5, 15, 30, 45 min and 1, 2, 3 h following the injection, and then were immediately centrifuged (4000 rpm, 10 min, 4°C) to obtain plasma samples. Plasma concentrations of the two peptides were measured by LC-MS/MS (ThermoFisher Scientific, USA). Pharmacokinetic parameters of JT003 and ADP355 were calculated using non-compartmental analysis in Phoenix WinNonlin (version 8.0, Pharsight, USA). The concentration values less than the LLOQ were set to zero for the analysis.” was added.

In the early concentration screening study of JT003, we did the lower nM level

doses. However, no good pharmacological activity was observed in vitro assay. The K_d from the HTRF experiments indicates the ability of receptor binding, and The activities evaluation at the cell assays indicates drug activities in vitro. The trend of the two is the same, but the order of magnitude for data is uncertain the same. This phenomenon is widespread in the field of drug research. For example: the K_d of Liraglutide for GLP-1 receptor binding is 40-50 pM, but for in vitro experiment, the concentration is 1-5 μM, which is 10^5-folds higher than the reported K_d (The Lancet 387, 679–690 (2016).). As can be seen from Supplementary Figure 3, Concentration ranges of 100 to 400 uM for JT003 indicated that the pharmacological activity is not good at 100 uM (it still doesn't reach a pharmacological saturation threshold), which it means higher concentrations could lead to better pharmacological activity. Thus, we think it is make more sense to use higher concentration to evaluate the pharmaceutical effects in vitro assay.

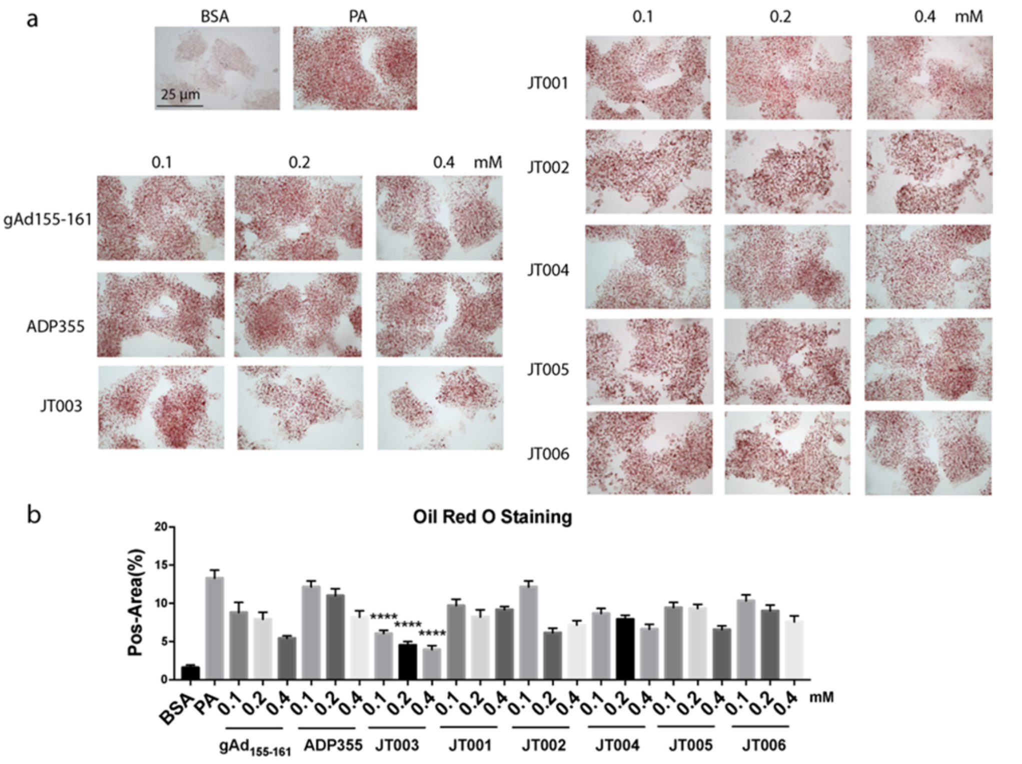

Figure S3. (a) Oil red o staining of PA induced lipid accumulation in HepG2 cell line after treated with different concentration of peptides. Triplicates were performed. (b) Positive areas were analyzed with ImageJ. Here and later, unless otherwise indicated, the. #p < 0.05, ##p <

0.01, ###p < 0.001, ####p < 0.0001 vs. the negative control; *p < 0.05, **p < 0.01, ***p < 0.001, ****p < 0.0001 vs. the model group. Data were shown as the mean ± SEM, statistical significance of the differences between each group was determined by Student's two-tailed t-test.

(7). The Core issue and comment 7：

Answer: Thank you for your suggestion. Although we did not include the nuclei staining in the ORO staining, the positive area quantification is according to the region with cells, which can easily tell from the cell adherence pattern. The flank area was not included in our quantification. Thus, we believe our analysis procedure is accurate. Based on the representative image we randomly picked for each group, we think it is hardly to say the cell number is reduced due to the JT003 treatment. Especially, it is comparable between the BSA group and JT003 group.

(8). The Core issue and comment 8：

Answer: Thank you for your suggestion. All the concentrations of compounds were used in each experiment were showed in the figure legend of the revised manuscript.

(9). The Core issue and comment 9：

Answer: Thank you for your comments. In page 6, paragraph 2, line 8, the sentence "Besides, in HFD combine with methionine and choline deficient (MCD) diet NASH mouse model, compare to the previous reported peptide ADP355, JT003 exhibited excellent ability of reducing body weight and inhibiting lipid accumulation (Supplementary Fig. 9, 10)." was revised with "Besides, in HFD combine with methionine and choline deficient (MCD) diet NASH mouse model, JT003 inhibited lipid accumulation significantly with a 20 times lower dosage than the previous reported peptide ADP355 (Supplementary Fig. 9, 10)."

(10). The Core issue and comment 10：

Answer: Thank you for your comments. We carefully quantified the steatosis area in different groups from HFD+MCD mouse model in figure S10. The effects in both groups are dose-dependent manners, although in JT003 there was a non-significant

increasing of steatosis area within high concentration.

(11). The Core issue and comment 11:

Answer: Thank you for your comments. Herein, we didn't discuss the fibrotic state in MCD model. In fact, this model was used to evaluate the fatty vacuolation induced by NASH. The CCl_4 induced liver fibrosis is the model which we utilized for studying JT003 on fibrotic state effects.

(12). The Core issue and comment 12:

Answer: Thank you very much for your comments. In page 3, paragraph 3, line 8, the sentence "GLP-1R agonists did not show significant response to liver fibrosis." was revised with "Instead of fibrosis, GLP-1R agonists were being actively pursued for the treatment of NASH." And the citation was added.

Reviewer: 3

Xu et al. evaluated positive effects of a peptide JT003 on NASH/fibrosis model. Overall effects of it coincide with adiponectin function on NASH model studied by using adiponectin KO mice. AMPK and PPARα signaling may also well coincide with globular adiponectin-AdipoRs signaling. **(13)** However, during the long revise period, it was reported that AdipoRs regulate membrane fluidity independent on adiponectin and thereby loss of them affects cellular signaling and metabolism (J Lipid Res. 2019 May;60(5):995-1004, Elife. 2019 Nov 26;8. pii: e47733). Therefore, it may be important to show the relationship between the modulation of membrane fluidity by the peptide agonist and AMPK/PPARalpha signaling with substantial experimental evidence. The interaction of native adiponectin existing in serum to AdipoRs was also reported not to exist (Elife. 2019 Oct 24;8. pii: e48675.). **(14)** Therefore, it may be also important now to show the difference between native adiponectin existing in serum and bacterially produced gAd on the binding to AdipoRs overexpressed in cells. Such evaluation may leave pharmacological significance of this compound.

The manuscript contains somewhat strange presentations in bar-graphs with representing western blots as pointed out by reviewer (The Core issue and comment 20). And such points were little revised by authors. **(15)** Indeed, in Figure 8C, there

can be seen a very intense band of PPARgamma in lane 7. However, in the corresponding graph, such high PPARgamma expression was not observed, with a very small error bar. The triplicate examination must have a large error bar in the bar-graph, if it contains such a highly different point.

----

(13). The Core issue and comment 13:

Answer: Thank you very much for your good suggestion. We considered that in the perspective of therapeutic aspects, it is more adequate to evaluate JT003 on AMPK/PPARalpha signaling here. We herein presented that the JT003 treatment significantly improved ER-mitochondrial axis function, which contributed to reduce HSCs activation. We believe our work fully respect the originality of science research in the perspective of therapeutic aspects. As for the study of the relationship between the modulation of membrane fluidity by the peptide agonist and AMPK/PPARalpha signaling, we will continue to study in the future

(14). The Core issue and comment 14:

Answer: Thank you very much for your comments. The C-terminal globular region of adiponectin (gAd) is the receptors binding region, there is no doubt to use gAd in our competitive binding assay. Besides, the native adiponectin existing in serum as the form of multimer, due to the disulfide bond formation mediated by Cystine in the protein, we think there is no ideal and efficiency method to purify the native adiponectin from serum. Also the commercial available adiponectin is obtained from bacterial.

(15). The Core issue and comment 15:

Answer: Thank you very much for your comments. For each experiment in our manuscript, three independent experiments were performed. We draw our conclusion from reproducible results. In Figure 8c, the PPARgamma was normalized to GAPDH expression level, bar-graph is based on the statistics of the intensity of the band. *The higher PPARgamma of lane 7 in the bar-graph was showed correctly.*

It would be highly appreciated if you are so kind to give a favorable consideration on it.

Sincerely,

Xianxing Jiang Ph.D
Professor
Medicinal Chemistry and Biochemistry
School of Pharmaceutical Sciences
Sun Yat-sen University
Waihuan East Road 132, Guangzhou University City
Guangzhou 510006, China
Mobile: +86 18819810352
E-mail: jiangxx5@mail.sysu.edu.cn

Reviewers' Comments:

Reviewer #2:
Remarks to the Author:
The authors have addressed many of the limitations through these two revision rounds. The HTRF experiments show that JT003 is improved in R1 & R2 engagement by ~10x vs the predecessor compound. Furthermore, the PK experiment also shows an improved circulating half-life (~7-8 fold). These are likely both contributing to the better PD performance vs the ADP compound.

The language used to describe the results and the subsequent discussion can use refinement, but I suggest to emphasize that the enhanced PK profile is a contributing factor to the improved in vivo efficacy of JT003 vs ADP, and it is not solely due to dual receptor activity & increased relative potency.

I am supportive of publication with carefully revised text.

Response to the Comments of Reviewers in Detail

Reviewer: 2

The authors have addressed many of the limitations through these two revision rounds. The HTRF experiments show that JT003 is improved in R1 & R2 engagement by ~10x vs the predecessor compound. Furthermore, the PK experiment also shows an improved circulating half-life (~7-8 fold). These are likely both contributing to the better PD performance vs the ADP compound.

The language used to describe the results and the subsequent discussion can use refinement, but I suggest to emphasize that the enhanced PK profile is a contributing factor to the improved in vivo efficacy of JT003 vs ADP, and it is not solely due to dual receptor activity & increased relative potency.

I am supportive of publication with carefully revised text.

Answer: Thank you very much. Also, many thanks for your recognition. According to your revision request, we have carefully proof-read the manuscript to minimize typographical and bibliographical errors. Some lengthy or inappropriate language used to describe the results and the subsequent discussion have been refined. In addition, we have emphasized that the enhanced PK profile is a contributing factor to the improved *in vivo* efficacy of JT003 vs ADP in the revised manuscript.

All authors participated in this study declare no potential, perceived, or real conflict of interest. We affirm that all authors have approved this submission and that the reported work has not received prior publication nor any portion of this work is under consideration of publication elsewhere. We highly appreciate your consideration of our manuscript. We are looking forward to your reply.
